# Supplementary material for: Exploring the photosensitizing potential of Nanoliposome Loaded Improved Toluidine Blue O (NLITBO) Against Streptococcus mutans: An in-vitro feasibility study
Source: PLoS One. 2024 Oct 30;19(10):e0312521. doi: 10.1371/journal.pone.0312521 (PMC11524459; doi:10.1371/journal.pone.0312521)

PLOS ONE

Exploring the photosensitizing potential of nanoliposome loaded improved Toluidine Blue O (NLITBO) against Streptococcus mutans: An in-vitro feasibility study

--Manuscript Draft--

**Manuscript Number:**	PONE-D-24-19897
**Article Type:**	Research Article
**Full Title:**	Exploring the photosensitizing potential of nanoliposome loaded improved Toluidine Blue O (NLITBO) against Streptococcus mutans: An in-vitro feasibility study
**Short Title:**	nanoliposome loaded improved Toluidine Blue O (NLITBO) against Streptococcus mutans
**Corresponding Author:**	Divya Gopinath<br>Ajman University<br>Ajman, UNITED ARAB EMIRATES
**Keywords:**	Streptococcus mutans, Toluidine Blue O, nanoliposomes, antibacterial efficacy, biofilm, dental caries
**Abstract:**	Background: Streptococcus mutans is a major contributor to dental caries due to its ability to produce acid and survive in biofilms. Microbial resistance towards common antimicrobial agents like chlorhexidine and triclosan has shifted the research towards antimicrobial Photodynamic therapy (PDT). In this context, Toluidine Blue O (TBO) is being explored for its photosensitizing properties against S. mutans. There is a huge variation in the effective concentration of TBO among the current studies owing to the differences in source of and delivery system TBO as well as the time, power and energy densities of light.<br>Objective: Hypothesizing the better antibacterial effectiveness, we have synthesised and characterised the improved TBO (ITBO) encapsulated in nanoliposomes. Further, we evaluated the zone of inhibition and Minimum inhibitory concentration (MIC) of nanoliposome-loaded improved TBO (NLITBO) against S. mutans suspensions in vitro.<br>Method: ITBO was synthesised per patent application number (202131053258 A dtd 26.05.2023). NLITBO was prepared using the thin-film hydration method, and a dynamic light scattering experiment determined the vesicle size, polydispersity index, and zeta potential. Surface features were characterized by Scanning and Transmission Electron microscopy. ITBO release from NLITBO was assessed using the extrapolation method. The antibacterial activity of the NLITBO was determined by evaluating the zone of inhibition (ZOI) in the S. mutans culture. The minimum inhibitory concentration (MIC) of NLITBO as a photosensitizer with red light (wavelength 650nm, power density 0.1 W/cm2, energy density 9-9.1 J/ cm2, 90seconds time) was evaluated against S. mutans cells by colourimetric method.<br>Results: Percentage drug loading, loading efficiency, yield percentage, vesicle size, polydispersity index (PDI) value, Zeta potential of NLTBO was reported as 9.3±0.4 %, 84.4±7.6%, 73.5%, 123.52 nm, 0.57, -39.54mV respectively. Clusters of unilamellar nanovesicles with smooth surfaces with no perforations were observed in SEM and TEM. The size of the vesicle was within 100 nm. At 24 hours, a cumulative 79.81 % of ITBO was released from NLITBO. ZOI of NLITBO (1 µg /ml) was 0.7 mm, and MIC was 0.6µg/ml.<br>Conclusion : For the first time, we have evaluated the antibacterial efficacy of NLITBO against S. mutans with a special focus on improvising the purity of TBO (ITBO) by a unique synthesis method and the delivery of ITBO by encapsulating in the nanoliposome. We have determined the MIC of NLITBO and optimized the light parameters. This would facilitate consistencies in further similar research and ease the clinical translation of aPDT using NLITBO to prevent and treat dental caries.
**Order of Authors:**	Swagatika Panda
	Lipsa Rout
	Neeta Mohanty
	Anurag Satpathy
	Bhabani sankar satapathy

Powered by Editorial Manager® and ProduXion Manager® from Aries Systems Corporation

	Shakti Rath
	Divya Gopinath
**Additional Information:**	
**Question**	**Response**
**Financial Disclosure**<br><br>Enter a financial disclosure statement that describes the sources of funding for the work included in this submission. Review the submission guidelines for detailed requirements. View published research articles from *PLOS ONE* for specific examples.<br><br>This statement is required for submission and **will appear in the published article** if the submission is accepted. Please make sure it is accurate.<br><br>**Funded studies**<br>Enter a statement with the following details:<br>• Initials of the authors who received each award<br>• Grant numbers awarded to each author<br>• The full name of each funder<br>• URL of each funder website<br>• Did the sponsors or funders play any role in the study design, data collection and analysis, decision to publish, or preparation of the manuscript?<br><br>Did you receive funding for this work?	The author(s) received no specific funding for this work.
**Competing Interests**<br><br>Use the instructions below to enter a competing interest statement for this submission. On behalf of all authors, disclose any competing interests that could be perceived to bias this work—acknowledging all financial support and any other relevant financial or non-financial competing interests.<br><br>This statement is required for submission and **will appear in the published article** if	The authors have declared that no competing interests exist.

the submission is accepted. Please make sure it is accurate and that any funding sources listed in your Funding Information later in the submission form are also declared in your Financial Disclosure statement.<br><br>View published research articles from *PLOS ONE* for specific examples.<br><br>**NO authors have competing interests**<br><br>Enter: *The authors have declared that no competing interests exist.*<br><br>**Authors with competing interests**<br><br>Enter competing interest details beginning with this statement:<br><br>*I have read the journal's policy and the authors of this manuscript have the following competing interests: [insert competing interests here]*<br><br>* typeset	
**Ethics Statement**<br><br>Enter an ethics statement for this submission. This statement is required if the study involved:<br><br>• Human participants<br>• Human specimens or tissue<br>• Vertebrate animals or cephalopods<br>• Vertebrate embryos or tissues<br>• Field research<br><br>Write "N/A" if the submission does not require an ethics statement.<br><br>General guidance is provided below. Consult the submission guidelines for detailed instructions. **Make sure that all information entered here is included in the Methods section of the manuscript.**	N/A

**Format for specific study types**<br><br>**Human Subject Research (involving human participants and/or tissue)**<br>• Give the name of the institutional review board or ethics committee that approved the study<br>• Include the approval number and/or a statement indicating approval of this research<br>• Indicate the form of consent obtained (written/oral) or the reason that consent was not obtained (e.g. the data were analyzed anonymously)<br><br>**Animal Research (involving vertebrate animals, embryos or tissues)**<br>• Provide the name of the Institutional Animal Care and Use Committee (IACUC) or other relevant ethics board that reviewed the study protocol, and indicate whether they approved this research or granted a formal waiver of ethical approval<br>• Include an approval number if one was obtained<br>• If the study involved *non-human primates*, add *additional details* about animal welfare and steps taken to ameliorate suffering<br>• If anesthesia, euthanasia, or any kind of animal sacrifice is part of the study, include briefly which substances and/or methods were applied<br><br>**Field Research**<br><br>Include the following details if this study involves the collection of plant, animal, or other materials from a natural setting:<br>• Field permit number<br>• Name of the institution or relevant body that granted permission	
**Data Availability**<br><br>Authors are required to make all data underlying the findings described fully available, without restriction, and from the time of publication. PLOS allows rare exceptions to address legal and ethical concerns. See the PLOS Data Policy and FAQ for detailed information.	Yes - all data are fully available without restriction

Powered by Editorial Manager® and ProduXion Manager® from Aries Systems Corporation

A Data Availability Statement describing where the data can be found is required at submission. Your answers to this question constitute the Data Availability Statement and **will be published in the article**, if accepted.<br><br>**Important:** Stating 'data available on request from the author' is not sufficient. If your data are only available upon request, select 'No' for the first question and explain your exceptional situation in the text box.<br><br>Do the authors confirm that all data underlying the findings described in their manuscript are fully available without restriction?	
**Describe where the data may be found in full sentences. If you are copying our sample text, replace any instances of XXX with the appropriate details.**<br><br>• If the data are **held or will be held in a public repository**, include URLs, accession numbers or DOIs. If this information will only be available after acceptance, indicate this by ticking the box below. For example: *All XXX files are available from the XXX database (accession number(s) XXX, XXX.)*.<br>• If the data are all contained **within the manuscript and/or Supporting Information files**, enter the following: *All relevant data are within the manuscript and its Supporting Information files.*<br>• If neither of these applies but you are able to provide **details of access elsewhere**, with or without limitations, please do so. For example:<br><br>*Data cannot be shared publicly because of [XXX]. Data are available from the XXX Institutional Data Access / Ethics Committee (contact via XXX) for researchers who meet the criteria for access to confidential data.*<br><br>*The data underlying the results presented in the study are available from (include the name of the third party*	All relevant data are within the manuscript and its Supporting Information files.

and contact information or URL).<br>• This text is appropriate if the data are owned by a third party and authors do not have permission to share the data.<br><br>* typeset	
Additional data availability information:	

Powered by Editorial Manager® and ProduXion Manager® from Aries Systems Corporation

Manuscript

Click here to access/download;Manuscript;manuscript Final.docx

Exploring the photosensitizing potential of nanoliposome loaded improved Toluidine Blue O (NLITBO) against Streptococcus mu[illegible]s: An in-vitro feasibility study

Swagatika Panda[1]*, Lipsa Rout[2], Neeta Mohanty[3], Anurag Satpathy[4], Bhabani sankar satapathy[5], Shakti Rath[6], Divya Gopinath[7,8]*,

1. Department of Oral Pathology and Microbiology, Institute of Dental Sciences, Siksha'O'Anusandhan deemed to be University. Bhubaneswar. Odisha. India. swagatikapanda@soa.ac.in
2. Institute of Dental Sciences, Siksha'O'Anusandhan deemed to be University. Bhubaneswar. Odisha. India. rout.lipsa21@gmail.com
3. Department of Oral Pathology and Microbiology, Institute of Dental Sciences, Siksha'O'Anusandhan deemed to be University. Bhubaneswar. Odisha. India. dr.neetamohanty@gmail.com
4. Department of Periodontics and Implantology, Institute of Dental Sciences, Siksha'O'Anusandhan deemed to be University. Bhubaneswar. Odisha. India. anuragsatpathy@soa.ac.in
5. Professor. School of Pharmaceutical Sciences (SPS), Siksha O Anusandhan Deemed to be University, Kalinga Nagar, Ghatikia, Bhubaneswar. Odisha. India. bhabanisatapathy@soa.ac.in
6. Department of Microbiology & Research, Institute of Dental Sciences, Siksha'O'Anusandhan deemed to be University. India. shaktirathmb@gmail.com
7. Basic medical and Dental Sciences Dept, College of Dentistry. Ajman University, UAE. d.gopinath@ajman.ac.ae
8. Centre of Medical and Bio-Allied Health Sciences and Research, Ajman University, Ajman, UAE

Corresponding author: Swagatika Panda and Divya Gopinath

E-mail: swagatikapanda@soa.ac.in and d.gopinath@ajman.ac.ae

Exploring the photosensitizing potential of nanoliposome loaded improved Toluidine Blue O (NLITBO) against *Streptococcus mutans*: An *in-vitro* feasibility study

Abstract:

Background: *Streptococcus mutans* is a major contributor to dental caries due to its ability to produce acid and survive in bi[illegible]ns. Microbial resistance towards common antimicrobial agents like chlorhexidine and triclosan has shifted the research towards antimicrobial Photodynamic therapy (PDT). In this context, Toluidine Blue O (TBO) is being explored for its photosensitizing properties against *S. mutans*. There is a huge variation in the effective concentration of TBO among the current studies owing to the differences in source of and delivery system TBO as well as the time, power and energy densities of light.

Objective: Hypothesizing the better antibacterial effectiveness, we have synthesised and characterised the improved TBO (ITBO) encapsulated in nanoliposomes. Further, we evaluated the zone of inhibition and Minimum inhibitory concentration (MIC) of nanoliposome-loaded improved TBO (NLITBO) against *S. mutans* suspensions in vitro.

Method: ITBO was synthesised per patent application number (202131053258 A dtd 26.05.2023). NLITBO was prepared using the thin-film hydration method, and a dynamic light scattering experiment determined the vesicle size, polydispersity index, and zeta potential. Surface features were characterized by Scanning and Transmission Electron microscopy. ITBO release from NLITBO was assessed using the extrapolation method. The antibacterial activity of the NLITBO was determined by evaluating the zone of inhibition (ZOI) in the *S. mutans* culture. The minimum inhibitory concentration (MIC) of NLITBO as a photosensitizer with red light (wavelength 650nm, power density 0.1 W/cm^2, energy density 9-9.1 J/ cm^2, 90seconds time) was evaluated against *S. mutans* cells by colourimetric method.

Results: Percentage drug loading, loading efficiency, yield percentage, vesicle size, polydispersity index (PDI) value, Zeta potential of NLTBO was reported as 9.3±0.4 %, 84.4±7.6%, 73.5%, 123.52 nm, 0.57, -39.54mV respectively. Clusters of unilamellar nanovesicles with smooth surfaces with no perforations were observed in SEM and TEM. The size of the vesicle was within 100 nm. At 24 hours, a cumulative 79.81 % of ITBO was released from NLITBO. ZOI of NLITBO (1 µg /ml) was 0.7 mm, and MIC was 0.6µg/ml.

Conclusion: For the first time, we have evaluated the antibacterial efficacy of NLITBO against *S. mutans* with a special focus on improvising the purity of TBO (ITBO) by a unique synthesis method and the delivery of ITBO by encapsulating in the nanoliposome. We have determined the MIC of NLITBO and optimized the light parameters. This would facilitate consistencies in further similar research and ease the clinical translation of aPDT using NLITBO to prevent and treat dental caries.

Keywords: *Streptococcus mutans*, Toluidine Blue O, nanoliposomes, antibacterial efficacy, biofilm, dental caries

Introduction

Dental caries, the most common infectious oral cavity disease, is a biofilm-mediated progressive disease that results in tooth cavitation.(1) *Streptococcus mutans* is the major cariogenic species in the dental biofilm. Tooth surface adhesion, biofilm formation, acidogenicity through glycolysis, aciduricity, and glucosyltransferase-mediated extracellular polysaccharide synthesis are the cariogenic attributes of *S. mutans*.(2) Antibacterial agents such as chlorhexidine and triclosan have effectively reduced caries bi[illegible]m. penetrating the biofilm matrix is a primary challenge for antibacterial agents. The extracellular polymeric substances within the matrix absorb the antibacterial agents, limiting their diffusion and availability within the biofilm interior. (3,4) Furthermore, the small pores of extracellular polymeric substances impede the passage of larger molecules, (5) necessitating significantly higher antimicrobial doses. Enzymatic inactivation of penetrated antibacterial agents within the biofilm further complicates its effectiveness.(6) Further, the antimicrobial effectiveness is challenged by the dormant growth phase of bacteria inside the biofilm.(7) Once inside the biofilm, antimicrobial agents primarily exert their effects by damaging cell walls or interfering with metabolic processes intracellularly.(8) This triggers the emergence of antimicrobial resistance through various mechanisms, including mutation, acquisition of exogenous resistance genes, horizontal transfer of resistance genes, and efflux pump activity.(7,9) Additionally, the mosaic structure of biofilms generates gradients of oxygen, nutrients, pH, and redox potential, leading to heterogeneity in growth rate and metabolic activity across different biofilm layers, which demand high specificity of antimicrobials. (10) Besides microbial resistance, allergic reactions to Chlorhexidine (11), ability to form calculus by being incorporated into the dental plaque (12), and increased microleakage (13). Further, it encourages research on exploring alternatives to these antimicrobials. Antimicrobial photodynamic therapy (aPDT) has appeared as a non-invasive alternative to modifying cariogenic biofilm.(13,14) where light of appropriate wavelength activates photosensitizers, thus releasing singlet oxygen. This singlet oxygen eventually causes tissue deterioration and cell death through necrosis or apoptosis.

The antibacterial effect of Toluidine Blue O (TBO) as a photosensitizer has been explored in a few studies, (15–19) particularly against gram-positive bacteria like *S. mutans* due to their

cationic and hydrophilic nature. However, there is wide variation in the effective antibacterial concentration of TBO against *streptococcus mutans*, which owes to inconsistency in the purity of TBO, delivery system of TBO and light parameters. Adequate penetration of the TBO into the biofilm is a noteworthy challenge for antibacterial efficacy, which in turn is dependent upon the purity and delivery system of TBO. Moreover, poor stability and limited bioavailability further limit the photosensitising properties of TBO. To optimize the photosensitising properties of TBO in harnessing the bacterial reduction outcome, we have attempted to minimise the above limitations of TBO-mediated aPDT by synthesising a purer form of TBO (ITBO). Nanoliposomes have been investigated for their biofilm penetrating potential, cell membrane-fusogenicity and biodegradability (9) which simultaneously improves the biocompatibility of the drug-loaded in it. Therefore, further, we attempted to load the ITBO in the core of the nanoliposome (NLITBO). The objective of the present research was to test the antibacterial efficacy of NLITBO against planktonic suspension of clinical strains of *S. mutans*.

Methodology:

1. Synthesising Improved Toluidine blue O (ITBO)

ITBO was synthesised by the method as suggested in the patent application **(**202131053258 A dtd 26.05.2023**)**

1. Preparation of NLITBO

For the preparation of drug-loaded nanoliposomes, plant-based phospholipids like soya-L-α-lecithin were used as the main lipid component. Along with that, cholesterol was also used to stabilize the lipid structure. The experimental nanoliposomes were developed using the thin film hydration method with the required modifications.

Briefly, the amount of soya-L-α-lecithin and cholesterol were dissolved in the required chloroform in a 250 ml round bottom flask. To this mixture, butylated hydroxyl toluene (2% w/v) was added as an antioxidant to prevent oxidation of lipids. The prepared mixture was then subjected to gentle rotation and evaporation of the solvent in a rotary vacuum evaporator (Rotavap, PBU-6, Superfit, Mumbai, India), connected with a water bath at 40 °C. Toluidine blue was added, and a thin film was formed along the inner wall of the round bottom flask. The flask was then kept in a desiccator overnight to remove organic solvent residues still in the thin film. On day 2, the formed thin film was hydrated with phosphate buffer saline (PBS), pH 7.4,

for 1 h at 130-150 rpm rotation. Following hydration, the mixture was subjected to sonication in a bath-type sonicator to reduce the size of liposomes to the nanoscale range. Following sonication, the formulation was kept in a refrigerator overnight at 4 °C. On day 3, the sample was subjected to cold centrifugation at 15,000 rpm for 45 minutes. The supernatant was discarded, and the sediments were collected and stored at –20 °C overnight. The pre-cooled samples were dried in a laboratory lyophilizer for 8-10 hours to obtain the dry powdered product.

2. **Physico-chemical characterization of NLITBO**

a) *Percentage Yield, Percentage drug loading and loading efficiency*: Lyophilized NLITBOs were weighed after each production batch, and the percentage yield was measured as % Yield= (Amount of NLITBOs obtained after lyophilization / Total amount of components used in the formulation batch) × 100. Briefly, the amount of lyophilized NLITBOs was dissolved in ethanol and water in a 7:3 ratio. The sample was then vortexed for 2 min, sonicated for 5 min and centrifuged at 12,000 rpm. The supernatant was examined at 631.0 nm using a UV/VIS spectrophotometer (Beckman, Fullerton, CA, USA). The following formula was calculated for the percentage drug loading and loading efficiency in NLITBOs.

Drug loading (%) = (Amount of TBO in NLITBOs / Amount of NLITBOs obtained) × 100

Drug loading efficiency (%) = (Practical TBO loading/ Theoretical TBO loading) × 100

b) *Determination of average vesicle size, polydispersity index (PDI) and Zeta potential:* To determine the average hydrodynamic size, the PDI and zeta potential of NLTBOs were evaluated using the Zeta sizer (Nano ZS 90, Malvern Instruments Ltd., UK) (*Satapathy et al., 2023*). Briefly, lyophilized NLITBOs were diluted with deionized water, followed by sonication for 5 minutes. At 25 ± 0.5 ºC, the sample was observed using a Zeta sizer.

c) Determination of surface features

The surface morphology and interior structure of the NLITBO nanovesicles were investigated using SEM and TEM, respectively. The lyophilized samples were spread out over a stub on a carbon tape. The sample was then vacuum dried, followed by gold coating over the surface, and was observed under SEM (JSM 6100; JEOL, Tokyo, Japan). NLITBO was suspended in deionized water and put onto the carbon-coated copper grid (mesh), which was then air-dried.

The dried sample was then analysed under TEM (Tecnai G2 Spirit BioTwin FP 5018/40, FEI, Hillsboro, USA).

d) ITBO release assessment in vitro

10 mg of lyophilized NLITBOs was suspended in phosphate-buffered saline (PBS, pH of 7.4) and was placed in a dialysis bag (Himedia dialysis membrane-60, Mumbai, India). Two open ends of the dialysis bag were tied together using cotton thread, and the assembly was placed in a beaker filled with 50 millilitres of PBS. The beaker was placed over a magnetic stirrer (rotation speed: 300 rpm), and at fixed intervals, 1 millilitre of the release medium was removed with simultaneous replenishment of fresh PBS. The samples were analysed using a UV-visible spectrophotometer at a fixed wavelength of 631.0 nm. The amount of ITBO released from NLTBOs was determined by the extrapolation method from the standard calibration curve.

e) Bacterial strain and culture conditio[illegible]

Clinical strains of *S. muta*[illegible] isolated from patients with caries, were cultured in blood agar (Hi-media) and maintained in the laboratory. The strains were cultured in Muller Hinton agar media for experimentation. Bacterial Inoculum was prepared by inoculating *S. mutans* culture in Muller Hinton broth and incubated until the culture reached the logarithmic growth phase. any test to see the growth.

f) Zone of inhibition

In vitro, the antibacterial activity of NLITBO was determined by the agar well diffusion method. 0.5 McFarland standard culture (1.5×108 cfu mL−1) of *S. mutans* was prepared in Muller-Hinton agar media (HI media, India). 100 µL inoculum of *S mutans* was spread onto Muller Hinton agar plates using a sterile cotton swab and allowed to dry, and wells were made with a sterile borer in the inoculated agar plates. 1 microgram of lyophilised NLITBO was dissolved in 1 ml of distilled water. 100 µL volumes of each test concentration were propelled into the culture plate of *S. mutans*. Then, the plate was incubated for 24 h at 37 °C. 10% aqueous Dimethyl sulfoxide (DMSO) was used as the negative control; 2% Chlorhexidine was used as the positive control. The antimicrobial activity of NLITBO was observed by recording the zone of inhibition. The experiments were performed in triplicates, and the mean diameter of the zone of inhibition (ZOI) with standard deviation was recorded.

g) Minimum inhibitory concentration determination

The Minimum inhibitory concentration (MIC) of NLITBO was determined by the colourimetric method in 96 well microtiter plates (Tarsons).(20) A stock solution of NLITBO in distilled water was made. The designing of the 96-well microplate was performed as follows: One column each was designed for positive and negative control. Seven test columns were composed of serial concentrations of NLITBO (0.9, 0.8, 0.7, 0.6, 0.5, 0.4, 0.3 µg/ ml respectively). The microbial culture was inoculated into Nutrient broth. The inoculum density was adjusted to approximately 0.5 McFarland standard (1-2 x 10^8 CFU/mL).100 µl of NLITBO solutions having concentrations ranging from 0.9 µg to 0.3 µg along with 100 µl of the prepared bacterial culture were added in each column of 96 well microtiter plate ensuring the final volume in each well is 200 µL. The plate was covered to prevent contamination and light and incubated at 37°C for 10 minutes. After incubation, each well was exposed to red LED light with a wavelength of 650nm and energy density approximating 9.1 J/cm^2. At the same energy density, the experiment was repeated thrice with three different combinations of power density and time (0.1 W/cm^2 + 90s, 0.08 W/cm^2 + 110s, 0.06 W/cm^2 + 150s). The light was applied to each well using a round tip with an 8 mm diameter attached to the light-emitting device (Novo Duo, Novolase, India). 20 µL of TTC solution (0.2% w/v) was added to each well, including the positive and negative controls. The positive control was without NLITBO, and the negative control was without the bacteria. Wells other than the receiving wells were blocked from light by aluminium foil. The tip of the light device accurately fits the surface of each well, which is approximately 7 mm in diameter, so a uniform sphere of light is applied in the suspension mix. The plate was incubated overnight at room temperature to allow the reduction of TTC by metabolically active microorganisms. The wells were compared to determine the lowest concentration of TBO, which shows no or minimal colour change or formazan formation. The colour change was read visually, and a stereomicroscope (Motic, USA) was used to observe red formazan precipitates in the wells. The experiment was conducted in triplicate, and the mean concentration of TBO at which bacterial growth was inhibited was accepted as the MIC.

Results

a) % Yield, % drug loading, and % loading efficiency of experimental NLITBOs

The three best formulations were selected from several formulation batches based on their drug-carrying capacity. Further, out of the three reported formulations, NLTBO-2 was found to be suitable (Table 1). The % drug loading for NLTBO-2 was 9.3±0.4 %, whereas 3.6±1.1% and 5.2±1.02 % for NLITBO-1 and NLITBO-3, respectively. NLITBO-2 also showed higher loading efficiency (84.4±7.6%) with yield percentage (73.5%) than the other two formulations (Table 1). Based on these parameters, we selected NLITBO-2 for further studies.

Table 1. Composition and percentage yield, loading and efficiency of three formulations

Formulation code	CHL: SL	ITBO Quantity (mg)	% yield	Practical % loading	% Loading efficiency
NLTBO -1	1:1	15	61.4	3.6±1.1	58.3 ±5.1
NLTBO -2	1:3	15	73.5	9.3±0.4	84.4±7.6
NLTBO -3	1:5	15	56.6	5.2±1.02	61.3 ±0.4

b) Size analysis and zeta potential of selected NLTBOs

DLS data revealed that the selected formulation (NLTBO-2) was well within the desired nano-size range (Z-avg = 123.52 nm) (Fig.1A). The PDI value was found to be 0.57. Zeta potential of NLTBO-2 was reported to be -39.54mV (Fig.1 B).

c) Surface and internal structure analysis

SEM and TEM results depicted the successful formulation of NLITBO within the desired nano-range. All formed nanovesicles were spherical with smooth surfaces (Figure 2A). Most NLTBO-2 had a homogeneous size distribution, and there was a strong correlation between the vesicle sizes and the average size found in the DLS data. TEM study depicted a clear formation of unilamellar vesicular structures (Figure 2B). Nanoliposomal vesicles with a spherical form, undistorted surface, and darker interior were observed in the TEM image (Figure 2B). The vesicles had no leaks or perforations, and the lamellarity was unbroken. The size of the vesicle was determined to be within 100 nm, which too supported by SEM and DLS data.

d) In vitro drug release study

The in vitro drug release study demonstrated sustained release characteristics of the NLITBO-2 at pH 7.4. At 24 h, a cumulative 79.81 % of ITBO was released from NLITBO-2 (Figure 3).

e*) Zone of inhibition*

Positive control, 2% chlorhexidine, showed a wider zone of inhibition (1.2±0.3mm), whereas the zone of inhibition produced by NLITBO was maximum (0.7±0.2mm) at 1 microgram/ml, intermediate (0.3±0.1mm) at 0.25 µg /ml and none at 0.25microgram/ml of NLITBO.

f) MIC of NLITBO

We observed no colour change in the test well containing 0.6 µg /ml (SD=0.03) NLITBO, indicating clear inhibition of bacterial growth to a gradual increase in the colour intensity as the concentration of NLITBO decreases. Therefore, we concluded that the MIC of NLITBO is 0.60 µg /ml (SD=0.03). Both positive and negative controls did not show any change.

Discussion

This experiment has explored the antibacterial efficacy of NLITBO against planktonic suspension of clinical strains of S mutans, a significant cariogenic species. This study assessed the potential of NLITBO as a photosensitiser in aPDT, focusing on the advantage of nanoliposomes as a delivery system and the purer form of TBO (ITBO) as a photosensitiser. The results showed that NLITBO exhibited significant antibacterial activity against *S. mutans.* The zone of inhibition produced by NLITBO (0.7mm) alone without PDT is slightly less than chlorhexidine (1.2mm) but promising enough to warrant further exploration of MIC of aPDT using NLITBO.

The successful synthesis of ITBO and incorporation into the nanoliposome was well characterized by the appropriate composition ratio of soya lecithin and cholesterol, high percentage yield (), drug loading capacity, and surface and interior characteristics. Incorporating soya α lecithin and cholesterol in 5 a 1 ratio has been found to extend the stability of cinnamon oil for 96 hrs and enhance the anti-biofilm action against methicillin-resistant staphylococcus aureus (MRSA) by almost 10 times as compared to free cinnamon oil. (21) The lower value of PDI (0.57) indicates a homogenous and narrow distribution pattern of the formulation. -The 39.5mV zeta potential of NLITBO classifies it as an anionic nanoliposome,

which helps maintain stability in suspension. Higher similar charges between the nanovesicles create strong repulsive forces, keeping vesicles separated for longer. Stable nano formulations have been reported to have a surface charge beyond -30 mV to +30 mV.(22,23) The drug release profiles exhibited a controlled release pattern over the study period, which is beneficial for effective clinical application, reducing dose and dose-related side effects. ITBO showed antibacterial activity even without photoactivation, as observed by ZOI in the present study, which implied inherent antibacterial properties of ITBO that are potentiated by red light application. This finding has negated by Shen et al (24) but supported by Nastri et al (25) who demonstrated the antibacterial effect of TBO at 10 µM concentration which was further potentiated by light application. The antibacterial activity of NLITBO without light may be attributed to the improved TBO and coating with anionic nanoliposome. NLITBO, owing to its zeta potential -39.5mV, is an anionic nanoliposome carrying cationic photosensitiser, ITBO. Anionic nanoliposomes may be less effective than cationic liposomes against gram-positive bacteria like *S. mutans*.(26) However, upon activation of the cationic photosensitiser, ITBO may penetrate the dental biofilm and *S .mutans* cell wall more effectively than cationic nanoliposome carrying cationic ITBO as it may not penetrate biofilm well due to the overall charge repulsion. Studies suggested that nanoliposomes loaded with cationic photosensitizers are designed for hydrophobic interaction with the negatively charged surface of gram-positive bacterial cells. This allows for the diffusion of nanoliposome-loaded photosensitisers into S mutans. (27) The liposome-bacteria fusion was shown to enhance the diffusion of the drugs into *Pseudomonas aeruginosa*. (28,29) This selective targeting enhances the specificity and efficacy of aPDT against gram-positive pathogens. Upon exposure to red light, the photosensitizer, ITBO loaded within the nanoliposomes, becomes excited. It undergoes photochemical reactions, leading to the generation of reactive oxygen species (ROS), such as singlet oxygen and free radicals. The generated ROS exert their antimicrobial effects by inducing oxidative damage to various cellular components of the gram-positive bacterial cells, eventually disrupting cellular functions and promoting cell death. This disruption facilitates the entry of ROS into the bacterial cytoplasm, enhancing their antimicrobial activity. Moreover, sustained release characteristics of the NLITBO-2, as found in this study, shall prove beneficial as there would be a consistent therapeutic effect even in the presence of saliva as the experiment was conducted at pH 7.4. A significant portion of ITBO (79.81 %) was released from NLITBO-2 by the end of 24 hours. This attribute can be advantageous in a targeted and sustained approach during aPDT against gram-positive bacteria, providing a promising strategy for

combating bacterial infections while minimising the risk of antimicrobial resistance and enhancing patient compliance.

This study has identified MIC of NLITBO against clinical strains of *streptococcus mutans* suspension with red light (wavelength – 650 nm, power density - 0.1mW/cm^2 for 90 seconds). Effective antimicrobial activity of TBO in *S mutans* suspension was observed in the concentration range of 0.01 to 3.08 mg/ml. (15–19,30–32) the effective concentration for anti-biofilm activity in *S. mutans* was 0.1mg/ml (19,33–35) and 41.067 mg/ml. (36) (34,35,37,38) Although the effective antibacterial concentration should vary according to the nature of bacterial culture, there is a huge difference in the effective concentration of TBO in studies on bacterial suspensions, too. (15–19,30–32) This variation may be attributed to the difference in strains of s mutans, light parameters such as energy density, power density, incubation time and time of red-light exposure across studies. Energy densities reported in different studies ranged from 9J/cm2 to 180J/cm2 (19,26,33,35–38), while power densities ranged from 100 mW to 500 mW. Duration of light exposure also varied from 30 seconds (39) to 12 minutes 50 seconds (17). Except for one (39) rest of the studies have used the commercial strain. In fact, the two studies (27,40) which evaluated the antimicrobial potential of TBO clinically in children with early childhood caries failed to significantly reduce the bacterial count of the saliva. This inconsistent result could be the absence of an appropriate drug delivery system. Nanoliposomal formulation, an effective drug delivery system, is known to enhance the bioavailability and biodistribution of the loaded drug.(40) Afrasiabi et al. (41) have shown nanoliposomes' enhanced antimicrobial photodynamic therapy containing curcumin and doxycycline against *Aggregatibacter actinomycetemcomitans*. Therefore, we, for the first time, have emphasised the nanoliposome encapsulation of ITBO and demonstrated that NLITBO is an effective antibacterial agent against clinical strains of *streptococcus mutans* under optimal red-light parameters (wavelength 650nm, power density 0.1 W, energy density 9-9.1 J, 90seconds time) with a MIC of 0.6µg/ml. These conditions are effective, and the biocompatible concentration of NLITBO and light energy dosage are selected to target the 24-hour incubated bacterial suspension of *S. mutans.*

Firstly, our study demonstrated the minimum inhibitory concentration (MIC) of NLITBO. The observed MIC of 0.60 µg/mL for NLITBO indicates its potent bactericidal activity, highlighting its potential as a promising candidate for caries prevention. Supporting this previous study (18) has also shown better antibiofilm action of TBO-silver nanoparticle conjugates on *S. mutans* than TBO alone. TBO has also been added with microbial efflux pump

inhibitors for successful biofilm inactivation. (42) The enhanced efficacy of NLITBO can be attributed to several factors. One plausible explanation is the improved bioavailability and targeted delivery of TBO facilitated by nanoliposomes. Nanoliposomes offer a stable and controlled release platform, allowing for prolonged exposure of TBO to bacterial cells, thus maximizing its antimicrobial effects and minimizing potential adverse effects on surrounding tissues. Additionally, the nanoliposomes get attached to the cell wall of bacteria and enhance ITBO penetration into the bacterial cytoplasm, where it gets photoactivated and the nascent oxygen released causes bacterial death. Furthermore, the nanoliposome carrier system may protect ITBO from degradation and enhance its retention within the oral cavity, prolonging its therapeutic effects.

Despite the promising findings of our study, several limitations should be considered. Firstly, our study was conducted in vitro, and further investigations are needed to assess the efficacy of NLITBO in vivo. Additionally, while our study focused on *S. mutans*, future research should explore its effects on other oral pathogens and its potential interactions with the oral microbiome. Furthermore, the safety profile of NLITBO, including potential cytotoxicity and adverse effects, warrants thorough evaluation before clinical translation. The sensitivity of planktonic bacteria to antimicrobial agents is far lower than biofilm-grown bacteria.(43) Therefore, further research may be directed to find out the biofilm inhibitory concentration and biofilm microbicidal concentration, which would effectively explore the efficacy, safety, and practical application of ITBO in the context of dental care to determine its role in preventing and managing infections associated with *S. mutans.*

In conclusion, our study demonstrates the enhanced antimicrobial efficacy of NLITBO and the MIC with optimum light parameters during aPDT against *Streptococcus mutans*. This research's purity and drug delivery system may have clinical benefits when exploring caries preventive agents. Further research is needed to validate our findings in vivo, assess safety profiles, and explore potential applications in clinical practice. Overall, NLITBO represents an innovative and promising approach to caries prevention, addressing critical challenges in antimicrobial therapy and paving the way for improved oral health outcomes.

Acknowledgement: Nil

Funding : The authors did not receive any funding for the conduct of this research. The APC is funded by Ajman University

Conflict of Interest: None to declare

Figure Legends

Figure 1: DLS demonstrating Size (A) and Zeta potential (B) of NLITBO

Figure 2: Surface structure in SEM (A) and interior structure in TEM (B) of NLITBO

Figure 3: In vitro release behaviour of NLITBO in PBS, pH 7.4

Figure 4: Zone of Inhibition 1. NLITBO (0.25 µg /µl) - no zone; 2. NLITBO (0.5 µg /ml) - 0.3 mm.; 3. NLITBO (1 µg /µl) - 0.7 mm ; 4. Negative Control - no zone; 5; Chlorhexidine 2% - 1.2 mm

Table legends

Table 1 – Composition and percentage yield, loading and efficiency of three formulations

References

1. Marcenes W, Kassebaum NJ, Bernabé E, Flaxman A, Naghavi M, Lopez A, et al. Global burden of oral conditions in 1990-2010: a systematic analysis. J Dent Res. 2013 Jul;92(7):592–7.

2. Lynch DJ, Michalek SM, Zhu M, Drake D, Qian F, Banas JA. Cariogenicity of Streptococcus mutans Glucan-Binding Protein Deletion Mutants. Oral Health Dent

Manag [Internet]. 2013 Dec [cited 2024 Apr 22];12(4):191–9. Available from: https://www.ncbi.nlm.nih.gov/pmc/articles/PMC4437697/

3. Gupta TT, Karki SB, Fournier R, Ayan H. Mathematical Modelling of the Effects of Plasma Treatment on the Diffusivity of Biofilm. Appl Sci [Internet]. 2018 Oct [cited 2024 Apr 17];8(10):1729. Available from: https://www.mdpi.com/2076-3417/8/10/1729

4. Oliveira M, Brugnera D, Piccoli R. Biofilms in the dairy industries: general aspects and the use of essential oils as a new control alternative. Rev Inst Laticínios Cândido Tostes. 2013 Feb 1;68:65–73.

5. Gilbert P, Allison DG, McBain AJ. Biofilms in vitro and in vivo: do singular mechanisms imply cross-resistance? J Appl Microbiol. 2002;92 Suppl:98S-110S.

6. Albayaty YN, Thomas N, Hasan S, Prestidge CA. Penetration of topically used antimicrobials through *Staphylococcus aureus* biofilms: A comparative study using different models. J Drug Deliv Sci Technol [Internet]. 2018 Dec 1 [cited 2024 Apr 17];48:429–36. Available from: https://www.sciencedirect.com/science/article/pii/S1773224718308256

7. Smith AW. Biofilms and antibiotic therapy: is there a role for combating bacterial resistance by the use of novel drug delivery systems? Adv Drug Deliv Rev. 2005 Jul 29;57(10):1539–50.

8. Neville N, Jia Z. Approaches to the Structure-Based Design of Antivirulence Drugs: Therapeutics for the Post-Antibiotic Era. Mol Basel Switz. 2019 Jan 22;24(3):378.

9. Dos Santos Ramos MA, Da Silva PB, Spósito L, De Toledo LG, Bonifácio BV, Rodero CF, et al. Nanotechnology-based drug delivery systems for control of microbial biofilms: a review. Int J Nanomedicine. 2018;13:1179–213.

10. Stewart PS, Franklin MJ. Physiological heterogeneity in biofilms. Nat Rev Microbiol. 2008 Mar;6(3):199–210.

11. Pemberton MN, Gibson J. Chlorhexidine and hypersensitivity reactions in dentistry. Br Dent J. 2012 Dec;213(11):547–50.

12. Zanatta FB, Antoniazzi RP, Rösing CK. Staining and calculus formation after 0.12% chlorhexidine rinses in plaque-free and plaque covered surfaces: a randomized trial. J Appl Oral Sci Rev FOB. 2010;18(5):515–21.

13. Soukos NS, Goodson JM. Photodynamic therapy in the control of oral biofilms. Periodontol 2000 [Internet]. 2011 [cited 2024 Apr 19];55(1):143–66. Available from: https://onlinelibrary.wiley.com/doi/abs/10.1111/j.1600-0757.2010.00346.x

14. Gursoy H, Ozcakir-Tomruk C, Tanalp J, Yilmaz S. Photodynamic therapy in dentistry: a literature review. Clin Oral Investig. 2013 May;17(4):1113–25.

15. Aragão MGB, Costa CAG de A, Lima RA, Rodrigues LKA, Duarte S, Zanin ICJ. Comparative Effect of Two Red Lights on Streptococcus mutans Biofilms and Assessment of Temperature Variances in Human Teeth During In Vitro Photodynamic Antimicrobial Chemotherapy. Photobiomodulation Photomed Laser Surg. 2019 Jan;37(1):31–7.

16. Araújo PV, Teixeira KIR, Lanza LD, Cortes ME, Poletto LTA. In vitro lethal photosensitization of S. mutans using methylene blue and toluidine blue O as photosensitizers. Acta Odontol Latinoam AOL. 2009;22(2):93–7.

17. Misba L, Zaidi S, Khan AU. Efficacy of photodynamic therapy against Streptococcus mutans biofilm: Role of singlet oxygen. J Photochem Photobiol B. 2018 Jun;183:16–21.

18. Misba L, Kulshrestha S, Khan AU. Antibiofilm action of a toluidine blue O-silver nanoparticle conjugate on Streptococcus mutans: a mechanism of type I photodynamic therapy. Biofouling. 2016;32(3):313–28.

19. Zanin ICJ, Lobo MM, Rodrigues LKA, Pimenta LAF, Höfling JF, Gonçalves RB. Photosensitization of in vitro biofilms by toluidine blue O combined with a light-emitting diode. Eur J Oral Sci. 2006 Feb;114(1):64–9.

20. Barnes V L, Heithoff DM, Mahan SP, House JK, Mahan MJ. Antimicrobial susceptibility testing to evaluate minimum inhibitory concentration values of clinically relevant antibiotics. STAR Protoc [Internet]. 2023 Aug 10 [cited 2024 May 14];4(3):102512. Available from: https://www.ncbi.nlm.nih.gov/pmc/articles/PMC10448204/

21. Cui H, Li W, Li C, Vittayapadung S, Lin L. Liposome containing cinnamon oil with antibacterial activity against methicillin-resistant Staphylococcus aureus biofilm. Biofouling. 2016;32(2):215–25.

22. Satapathy BS, Biswal B, Pattnaik S, Parida R, Sahoo RN. A mucoadhesive nanolipo gel containing Aegle marmelos gum to enhance transdermal effectiveness of linezolid for vaginal infection: In vitro evaluation, in vitro-ex vivo correlation, pharmacokinetic studies. Int J Pharm. 2023 Dec 15;648:123542.

23. Satapathy BS, Kumar LA, Pattnaik G, Barik B. Lomustine Incorporated Lipid Nanostructures Demonstrated Preferential Anticancer Properties in C6 Glioma Cell Lines with Enhanced Pharmacokinetic Profile in Mice. Acta Chim Slov. 2021 Dec 15;68(4):970–82.

24. Shen J, Liang Q, Su G, Zhang Y, Wang Z, Baudouin C, et al. In Vitro Effect of Toluidine Blue Antimicrobial Photodynamic Chemotherapy on Staphylococcus epidermidis and Staphylococcus aureus Isolated from Ocular Surface Infection. Transl Vis Sci Technol [Internet]. 2019 Jun 21 [cited 2024 May 14];8(3):45. Available from: https://www.ncbi.nlm.nih.gov/pmc/articles/PMC6590089/

25. Nastri L, Donnarumma G, Porzio C, De Gregorio V, Tufano MA, Caruso F, et al. Effects of Toluidine Blue-Mediated Photodynamic Therapy on Periopathogens and Periodontal Biofilm: In Vitro Evaluation. Int J Immunopathol Pharmacol [Internet]. 2010 Oct 1 [cited 2024 May 14];23(4):1125–32. Available from: https://doi.org/10.1177/039463201002300416

26. Drulis-Kawa Z, Dorotkiewicz-Jach A, Gubernator J, Gula G, Bocer T, Doroszkiewicz W. The interaction between Pseudomonas aeruginosa cells and cationic PC:Chol:DOTAP liposomal vesicles versus outer-membrane structure and envelope properties of bacterial cell. Int J Pharm. 2009 Feb 9;367(1–2):211–9.

27. Beaulac C, Sachetelli S, Lagace J. In-vitro bactericidal efficacy of sub-MIC concentrations of liposome-encapsulated antibiotic against gram-negative and gram-positive bacteria. J Antimicrob Chemother. 1998 Jan;41(1):35–41.

28. Sachetelli S, Khalil H, Chen T, Beaulac C, Sénéchal S, Lagacé J. Demonstration of a fusion mechanism between a fluid bactericidal liposomal formulation and bacterial cells. Biochim Biophys Acta. 2000 Feb 15;1463(2):254–66.

29. Mugabe C, Halwani M, Azghani AO, Lafrenie RM, Omri A. Mechanism of Enhanced Activity of Liposome-Entrapped Aminoglycosides against Resistant Strains of Pseudomonas aeruginosa. Antimicrob Agents Chemother [Internet]. 2006 Jun [cited 2024 Apr 9];50(6):2016–22. Available from: https://www.ncbi.nlm.nih.gov/pmc/articles/PMC1479138/

30. Vahabi S, Fekrazad R, Ayremlou S, Taheri S, Zangeneh N. The effect of antimicrobial photodynamic therapy with radachlorin and toluidine blue on streptococcus mutans: an in vitro study. J Dent Tehran Iran. 2011;8(2):48–54.

31. Fekrazad R, Seraj B, Chiniforush N, Rokouei M, Mousavi N, Ghadimi S. Effect of antimicrobial photodynamic therapy on the counts of salivary Streptococcus mutans in children with severe early childhood caries. Photodiagnosis Photodyn Ther. 2017 Jun;18:319–22.

32. Hakimiha N, Khoei F, Bahador A, Fekrazad R. The susceptibility of Streptococcus mutans to antibacterial photodynamic therapy: a comparison of two different photosensitizers and light sources. J Appl Oral Sci Rev FOB. 2014 Apr;22(2):80–4.

33. de Sousa Farias SS, Nemezio MA, Corona S a. M, Aires CP, Borsatto MC. Effects of low-level laser therapy combined with toluidine blue on polysaccharides and biofilm of Streptococcus mutans. Lasers Med Sci. 2016 Jul;31(5):1011–6.

34. Balhaddad AA, AlQranei MS, Ibrahim MS, Weir MD, Martinho FC, Xu HHK, et al. Light Energy Dose and Photosensitizer Concentration Are Determinants of Effective Photo-Killing against Caries-Related Biofilms. Int J Mol Sci. 2020 Oct 15;21(20):7612.

35. Teixeira AH, Pereira ES, Rodrigues LKA, Saxena D, Duarte S, Zanin ICJ. Effect of photodynamic antimicrobial chemotherapy on in vitro and in situ biofilms. Caries Res. 2012;46(6):549–54.

36. de Oliveira FS, Cruvinel T, Cusicanqui Méndez DA, Dionísio EJ, Rios D, Machado MAAM. The *in vitro* effect of Antimicrobial Photodynamic Therapy on dental

microcosm biofilms from partially erupted permanent molars: A pilot study. Photodiagnosis Photodyn Ther [Internet]. 2018 Mar 1 [cited 2024 Apr 10];21:163–7. Available from: https://www.sciencedirect.com/science/article/pii/S1572100017304076

37. Shirato M, Nakamura K, Kanno T, Lingström P, Niwano Y, Örtengren U. Time-kill kinetic analysis of antimicrobial chemotherapy based on hydrogen peroxide photolysis against Streptococcus mutans biofilm. J Photochem Photobiol B. 2017 Aug;173:434–40.

38. Zanin ICJ, Gonçalves RB, Junior AB, Hope CK, Pratten J. Susceptibility of Streptococcus mutans biofilms to photodynamic therapy: an in vitro study. J Antimicrob Chemother. 2005 Aug;56(2):324–30.

39. Fekrazad R, Khoei F, Hakimiha N, Bahador A. Photoelimination of Streptococcus mutans with two methods of photodynamic and photothermal therapy. Photodiagnosis Photodyn Ther. 2013 Dec;10(4):626–31.

40. Fatfat Z, Karam M, Maatouk B, Fahs D, Gali-Muhtasib H. Chapter 7 - Nanoliposomes as safe and efficient drug delivery nanovesicles. In: Nayak AK, Hasnain MS, Laha B, Maiti S, editors. Advanced and Modern Approaches for Drug Delivery [Internet]. Academic Press; 2023 [cited 2024 Apr 8]. p. 159–97. Available from: https://www.sciencedirect.com/science/article/pii/B9780323916684000022

41. Afrasiabi S, Partoazar A, Chiniforush N. In vitro study of nanoliposomes containing curcumin and doxycycline for enhanced antimicrobial photodynamic therapy against Aggregatibacter actinomycetemcomitans. Sci Rep [Internet]. 2023 Jul 18 [cited 2024 Apr 8];13(1):11552. Available from: https://www.nature.com/articles/s41598-023-38812-4

42. Kishen A, Upadya M, Tegos GP, Hamblin MR. Efflux pump inhibitor potentiates antimicrobial photodynamic inactivation of Enterococcus faecalis biofilm. Photochem Photobiol. 2010;86(6):1343–9.

43. Lindhe J, Karring T, Lang NP. Clinical periodontology and implant dentistry. 4th ed. Oxford, UK: Blackwell; 2003.

Figure 1 Click here to access/download;Figure;Figure 1.png

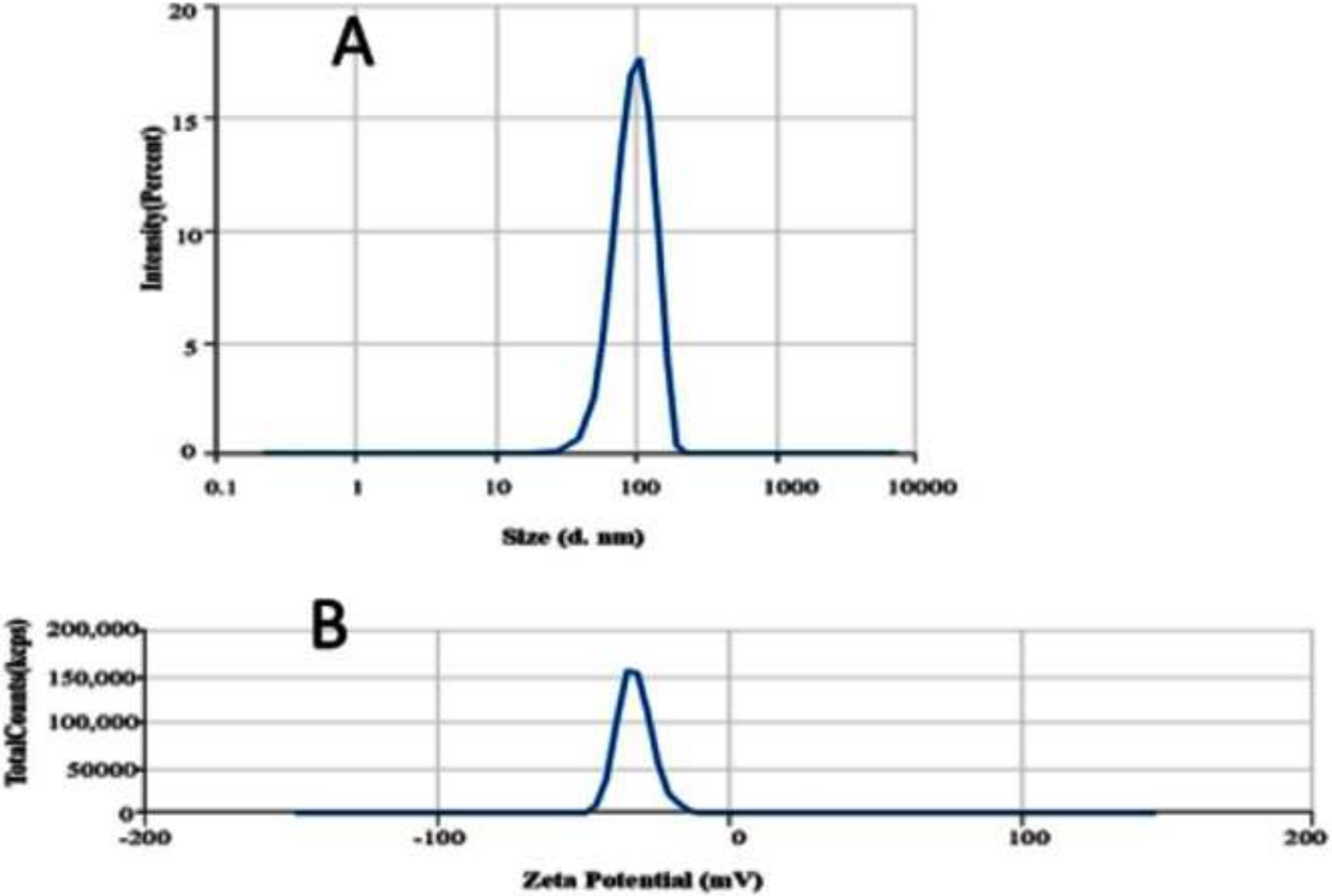

Figure 2

Click here to access/download;Figure;Figure 2.png

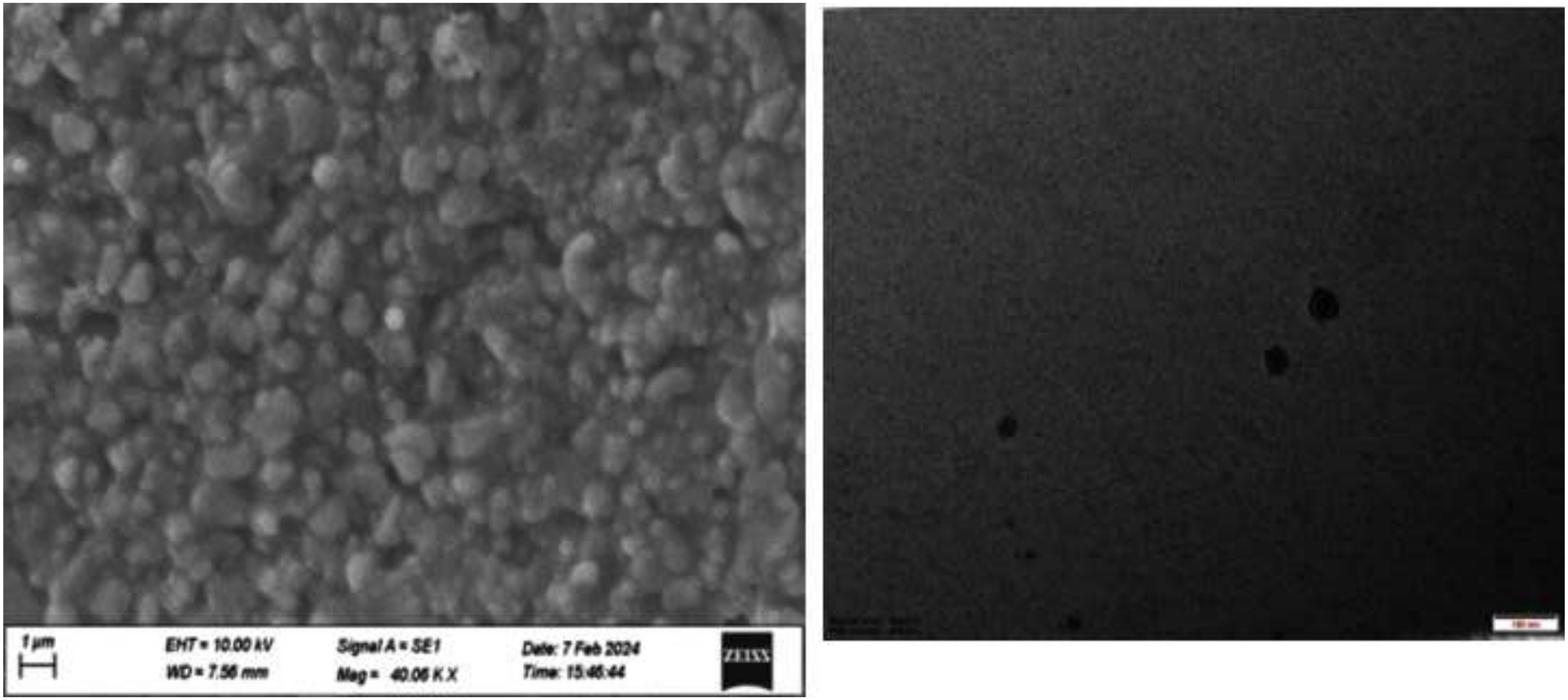

Figure 3

Click here to access/download;Figure;Figure 3.png

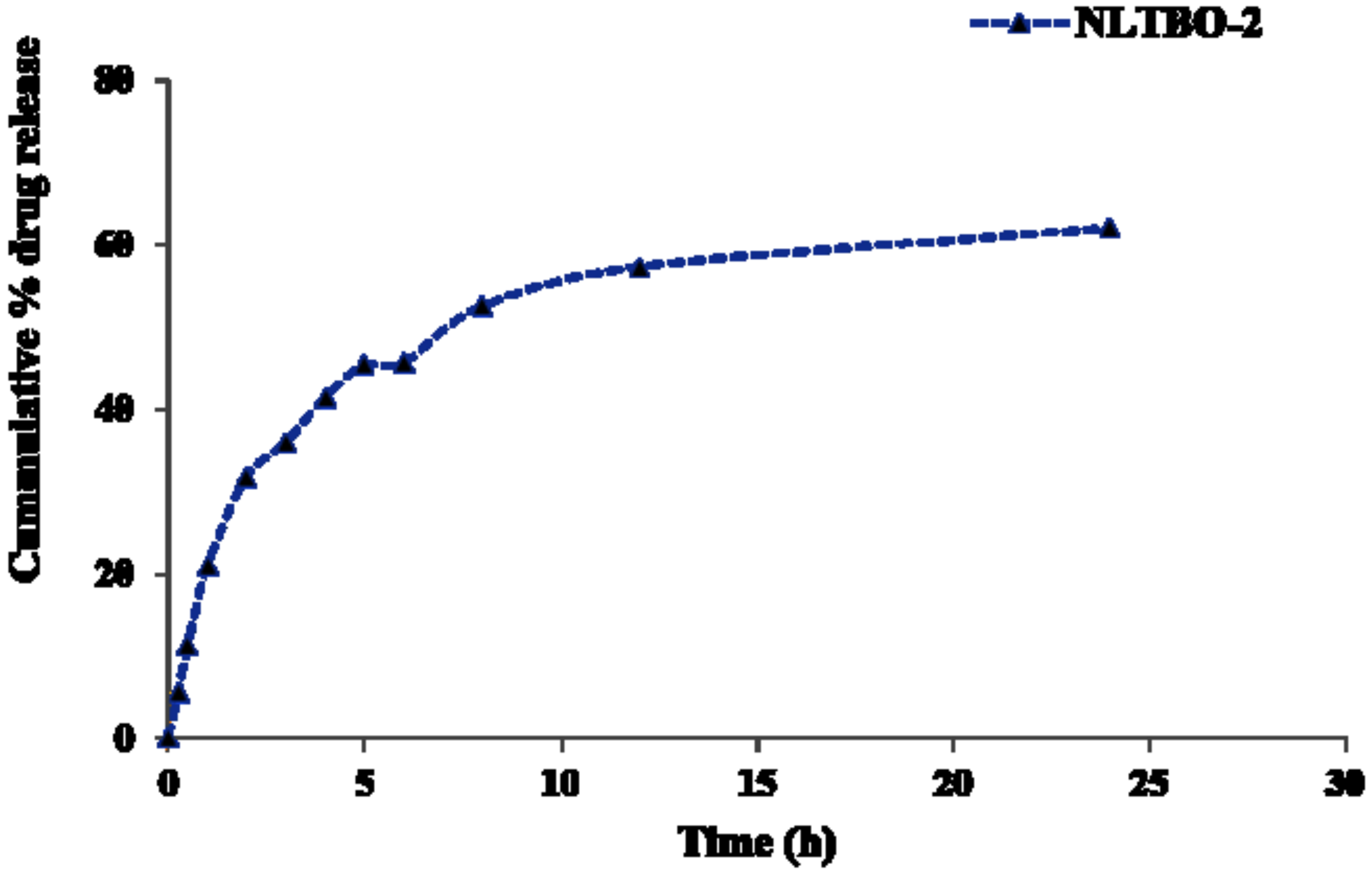

Figure 4

Click here to access/download;Figure;Figure 4.jpg

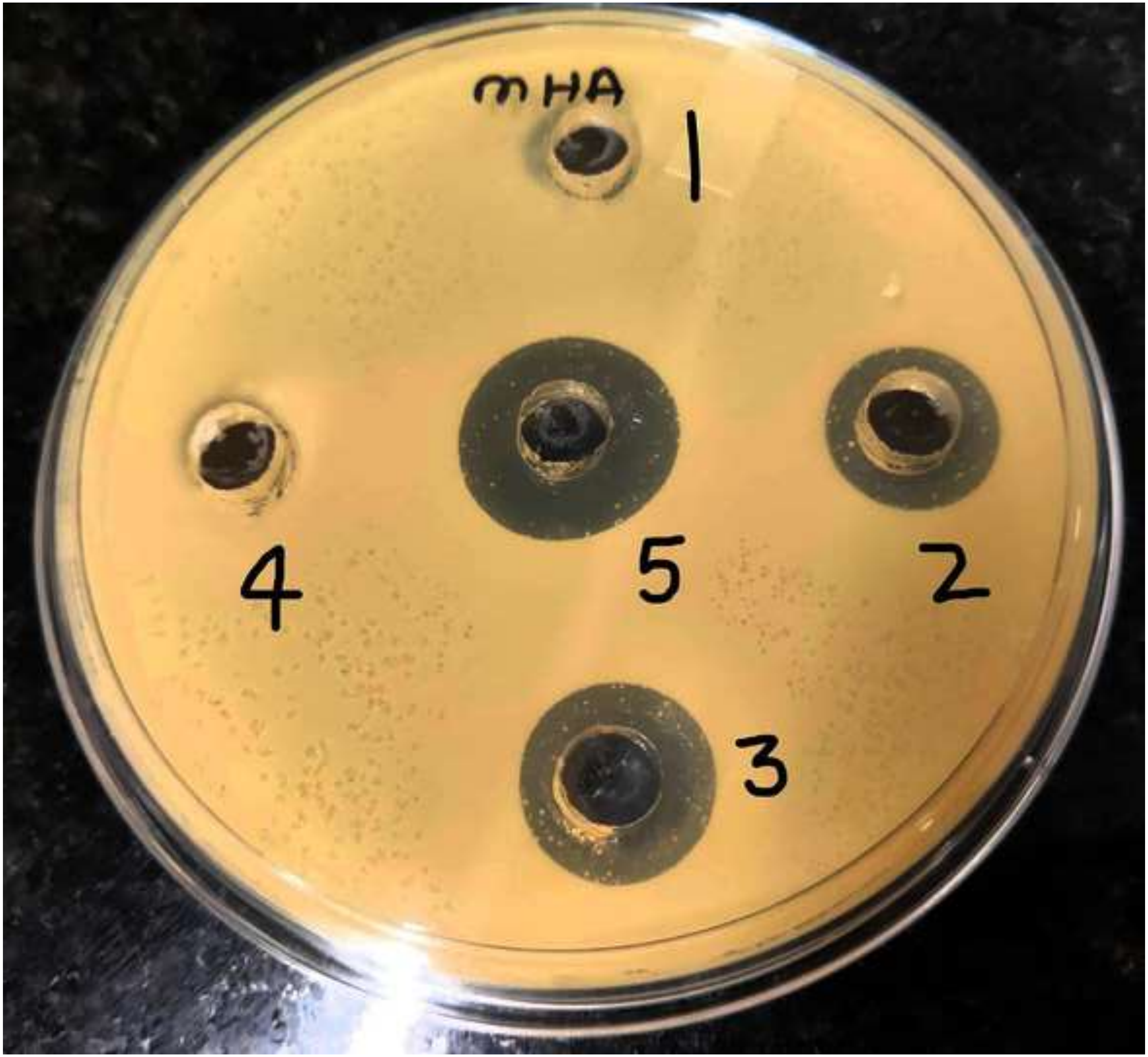

Supplement: Attachment — Submitted filename: PONE-D-24-19897_reviewer (1).pdf [file pone.0312521.s001.pdf]
