# Supplementary material for: Exploring the photosensitizing potential of Nanoliposome Loaded Improved Toluidine Blue O (NLITBO) Against Streptococcus mutans: An in-vitro feasibility study
Source: PLoS One. 2024 Oct 30;19(10):e0312521. doi: 10.1371/journal.pone.0312521 (PMC11524459; doi:10.1371/journal.pone.0312521)
Supplement: Attachment — Submitted filename: PONE-D-24-19897_R1_reviewer.pdf [file pone.0312521.s003.pdf]

PLOS ONE

Exploring the photosensitizing potential of nanoliposome loaded improved Toluidine Blue O (NLITBO) against Streptococcus mutans: An in-vitro feasibility study

--Manuscript Draft--

**Manuscript Number:**	PONE-D-24-19897R1
**Article Type:**	Research Article
**Full Title:**	Exploring the photosensitizing potential of nanoliposome loaded improved Toluidine Blue O (NLITBO) against Streptococcus mutans: An in-vitro feasibility study
**Short Title:**	nanoliposome loaded improved Toluidine Blue O (NLITBO) against Streptococcus mutans
**Corresponding Author:**	Divya Gopinath<br>Ajman University<br>Ajman, UNITED ARAB EMIRATES
**Keywords:**	Streptococcus mutans, Toluidine Blue O, nanoliposomes, antibacterial efficacy, biofilm, dental caries
**Abstract:**	Background: Streptococcus mutans is a major contributor to dental caries due to its ability to produce acid and survive in biofilms. Microbial resistance towards common antimicrobial agents like chlorhexidine and triclosan has shifted the research towards antimicrobial Photodynamic therapy (PDT). In this context, Toluidine Blue O (TBO) is being explored for its photosensitizing properties against S. mutans. There is a huge variation in the effective concentration of TBO among the current studies owing to the differences in source of and delivery system TBO as well as the time, power and energy densities of light.<br>Objective: Hypothesizing the better antibacterial effectiveness, we have synthesised and characterised the improved TBO (ITBO) encapsulated in nanoliposomes. Further, we evaluated the zone of inhibition and Minimum inhibitory concentration (MIC) of nanoliposome-loaded improved TBO (NLITBO) against S. mutans suspensions in vitro.<br>Method: ITBO was synthesised per patent application number (202131053258 A dtd 26.05.2023). NLITBO was prepared using the thin-film hydration method, and a dynamic light scattering experiment determined the vesicle size, polydispersity index, and zeta potential. Surface features were characterized by Scanning and Transmission Electron microscopy. ITBO release from NLITBO was assessed using the extrapolation method. The antibacterial activity of the NLITBO was determined by evaluating the zone of inhibition (ZOI) in the S. mutans culture. The minimum inhibitory concentration (MIC) of NLITBO as a photosensitizer with red light (wavelength 650nm, power density 0.1 W/cm2, energy density 9-9.1 J/ cm2, 90seconds time) was evaluated against S. mutans cells by colourimetric method.<br>Results: Percentage drug loading, loading efficiency, yield percentage, vesicle size, polydispersity index (PDI) value, Zeta potential of NLTBO was reported as 9.3±0.4 %, 84.4±7.6%, 73.5%, 123.52 nm, 0.57, -39.54mV respectively. Clusters of unilamellar nanovesicles with smooth surfaces with no perforations were observed in SEM and TEM. The size of the vesicle was within 100 nm. At 24 hours, a cumulative 79.81 % of ITBO was released from NLITBO. ZOI of NLITBO (1 µg /ml) was 0.7 mm, and MIC was 0.6µg/ml.<br>Conclusion : For the first time, we have evaluated the antibacterial efficacy of NLITBO against S. mutans with a special focus on improvising the purity of TBO (ITBO) by a unique synthesis method and the delivery of ITBO by encapsulating in the nanoliposome. We have determined the MIC of NLITBO and optimized the light parameters. This would facilitate consistencies in further similar research and ease the clinical translation of aPDT using NLITBO to prevent and treat dental caries.
**Order of Authors:**	Swagatika Panda
	Lipsa Rout
	Neeta Mohanty
	Anurag Satpathy
	Bhabani sankar satapathy

Powered by Editorial Manager® and ProduXion Manager® from Aries Systems Corporation

	Shakti Rath
	Divya Gopinath
**Response to Reviewers:**	Reviewer # 1<br>Topic is innovative. Manuscript is well written. Statistical test is mentioned. All references from reference list are depicted in the manuscript. Conclusion is in conformity with the objectives of the study.<br>Author response– Thank you for your encouraging words.<br><br>Reviewer #2:<br><br>Reviewer comment 1: The manuscript is quite a preliminary study. Without proper data, its not ready to be published.in Biofilms, there is always different bacterial species, so targeting alone, S. mutans is not the cure of the disease.<br>Author Response - While we acknowledge the complexity of biofilm communities and the presence of multiple bacterial species, this study was designed as a feasibility study to explore the efficacy of NLITBO against the primary contributor of the cariogenic biofilm which is Streptococcus mutans. Future research will involve expanding the scope to include other bacterial species commonly found in the biofilm, thereby providing a more comprehensive understanding of the potential of NLITBO in targeting polymicrobial biofilms. Our findings lay the groundwork for such studies by demonstrating the photosensitizing potential of NLITBO against a critical cariogenic species.<br>Reviewer comment 2 The treatment of planktonic suspension will not address the real problem. Biofilm existence comprising of S mutans and many others in case of dental caries.<br>Author Response – Yes, We agree with the reviewer and acknowledge that the treatment of planktonic suspensions will not address the real problem. However, our study aims to establish a foundational understanding of the antibacterial efficacy of NLITBO against S. mutans in planktonic form as a preliminary step. The promising result of this study may guide the next logical step to evaluate its effectiveness on dental biofilms.<br>Reviewer comment 3 Why both SEM and TEM. One was sufficient?<br>Author Response: We studied the surface morphology of NLITBO by SEM and internal structure of NLITBO by TEM so that both surface and internal properties are evaluated<br>Reviewer comment 4 Please prepare a biofilm of dental bacteria in vitro and then test the formulation. In planktonic mode against one bacteria. Of no use<br>Author response: Thank you for the insight. We agree that testing NLITBO in dental biofilm would provide a clinically relevant evaluation of its antibacterial efficacy. However, due to the time constraint and complexity of establishing a reliable in vitro dental biofilm model, it may not be feasible to conduct such experiment. Planktonic solutions are often used as a first line evaluation of any novel antibacterial agent's efficacy and the result guides further research. We appreciate your concern and looking forward to explore this avenue in further studies.<br>Reviewer comment 5 :ATCC or accession number of S mutans<br>Author response: The S mutans strain was isolated clinically. The methods has been described in page 8 line 228-229<br>Reviewer comment 6 Statistical analysis?<br>Author response: Thank you for your valuable suggestions. We have elaborated more on the statistical analysis on page 10, line 278-280<br><br>Reviewer #3:<br>In this article, the authors prepared NLITBO, characterized its features, and evaluated the antibacterial activity against S.mutans. This would provide a new approach for preventing and treating dental caries.<br>Author response– Thank you for your encouraging words.<br>Reviewer comment 1<br>Introduction<br>-Line # 87-91: Add the latest reference; https://doi.org/10.1016/j.jtemb.2024.127448<br>Author response: We have added the reference as suggested.<br>Reviewer comment 2

Powered by Editorial Manager® and ProduXion Manager® from Aries Systems Corporation

	-Line # 93-103: Add more detail about the biofilm and the effect of antibacterial agent on the biofilm. Compare with the other antibacterial agents and also add how the antimicrobial agent used in study are helpful in pharmaceutical industries.<br>Author response: Thank you for the insightful comments. We have added the further details of biofilm including effect of antibacterial agents on the biofilm. We also compared with other antibacterial agents and added the implication of this antimicrobial agent, i.e NLITBO in pharmaceutical industry.<br>Reviewer comment 3<br>Methodology<br>Add new separate heading with detail of reagents/chemicals, instrumentation, media etc. used in present study.<br>Author response: We have done the modification as suggested.<br>Reviewer comment 4<br>-Line # 136-138: Add the reference.<br>Author response: We have added the reference as suggested<br>Reviewer comment 5<br>-Line # 139: Reference<br>Author response: We have added the reference as suggested<br>Reviewer comment 6<br>-Add the references in each section of Methodology.<br>Author response: We have added the reference as suggested<br>Reviewer comment 7<br>Results<br>-Make sure bacterial species name must be written in correct form in all text.<br>Author response: We have done the modification as suggested.<br>Reviewer comment 8<br>Discussion<br>-Line #: 282, 283: This is the part of result section<br>Author response: We have done the modification as suggested.<br>Reviewer comment 9<br>-Line # 286-289: Why ZOI produced by NLITBO were higher than those of chlorhexidine, give strong reason.<br>Author response: Mean ZOI produced by NLITBO is not higher but not significantly different from mean ZOI produced by Chlorhexidine. As per your valuable suggestions I have substantiated with possible reasons in the discussion.<br>Reviewer comment 10<br>-Line # 292: Add reference.<br>Author response: Those empty parentheses is to write the values as found in this study. We have added the details.<br>Reviewer comment 11<br>-Line # 296, 297: Cite the proper reference.<br>Author response: We have added the reference as suggested<br>Reviewer comment 12<br>-Line # 302: Reference?<br>Author response: We have added the reference as suggested<br><br>Reviewer comment 13<br>-Line # 320: Reference?<br>Author response: We have added the reference as suggested<br>Reviewer comment 14<br>-S. mutans, correct it with same pattern in all text.<br>Author response: We have done the modification as suggested.<br>Reviewer comment 15<br>-Add the explanation, how the NPs affect on the biofilm producing bacterial structure?<br>Author response: We have added the explanation as suggested<br>Reviewer comment 16<br>-Lines # 345-348: Unnecessary statement. Is this related to this study?<br>Author response :Thank you for raising the concern. We believe the sentences are not explanatory enough for which we have edited these lines again. These sentences are a critical analysis of the studies which were conducted to extablish the effectiveness of TBO as a photosensitizer. Only one study has used the clinical strain similar to our study. In general, clinical strains exhibit more genetic diversity and the findings on clinical strains are applicable in clinical settings. Therefore, we have highlighted the broader applicability of our results. Similarly contradicting our establishment of

Powered by Editorial Manager® and ProduXion Manager® from Aries Systems Corporation

	antibacterial efficacy of NLITBO, two studies could not prove clinical effectiveness of TBO in early childhood caries, which may be attributed to the use of laboratory grade TBO, lack of appropriate delivery method and/or lack of optimum light parameters. In this light we have discussed the importance of nanoliposome as a drug delivery system.
Additional Information:	
Question	Response
**Financial Disclosure**<br><br>Enter a financial disclosure statement that describes the sources of funding for the work included in this submission. Review the submission guidelines for detailed requirements. View published research articles from *PLOS ONE* for specific examples.<br><br>This statement is required for submission and **will appear in the published article** if the submission is accepted. Please make sure it is accurate.<br><br>**Funded studies**<br>Enter a statement with the following details:<br>• Initials of the authors who received each award<br>• Grant numbers awarded to each author<br>• The full name of each funder<br>• URL of each funder website<br>• Did the sponsors or funders play any role in the study design, data collection and analysis, decision to publish, or preparation of the manuscript?<br><br>Did you receive funding for this work?	The author(s) received no specific funding for this work.
**Competing Interests**<br><br>Use the instructions below to enter a competing interest statement for this submission. On behalf of all authors, disclose any competing interests that could be perceived to bias this work—acknowledging all financial support and any other relevant financial or non-financial competing interests.	The authors have declared that no competing interests exist.

Powered by Editorial Manager® and ProduXion Manager® from Aries Systems Corporation

This statement is required for submission and **will appear in the published article** if the submission is accepted. Please make sure it is accurate and that any funding sources listed in your Funding Information later in the submission form are also declared in your Financial Disclosure statement.<br><br>View published research articles from *PLOS ONE* for specific examples.<br><br>**NO authors have competing interests**<br><br>Enter: *The authors have declared that no competing interests exist.*<br><br>**Authors with competing interests**<br><br>Enter competing interest details beginning with this statement:<br><br>*I have read the journal's policy and the authors of this manuscript have the following competing interests: [insert competing interests here]*<br><br>* typeset	
**Ethics Statement**<br><br>Enter an ethics statement for this submission. This statement is required if the study involved:<br><br>• Human participants<br>• Human specimens or tissue<br>• Vertebrate animals or cephalopods<br>• Vertebrate embryos or tissues<br>• Field research<br><br>Write "N/A" if the submission does not require an ethics statement.<br><br>General guidance is provided below. Consult the submission guidelines for detailed instructions. **Make sure that all information entered here is included in the Methods section of the manuscript.**	N/A

**Format for specific study types**<br><br>**Human Subject Research (involving human participants and/or tissue)**<br>• Give the name of the institutional review board or ethics committee that approved the study<br>• Include the approval number and/or a statement indicating approval of this research<br>• Indicate the form of consent obtained (written/oral) or the reason that consent was not obtained (e.g. the data were analyzed anonymously)<br><br>**Animal Research (involving vertebrate animals, embryos or tissues)**<br>• Provide the name of the Institutional Animal Care and Use Committee (IACUC) or other relevant ethics board that reviewed the study protocol, and indicate whether they approved this research or granted a formal waiver of ethical approval<br>• Include an approval number if one was obtained<br>• If the study involved *non-human primates*, add *additional details* about animal welfare and steps taken to ameliorate suffering<br>• If anesthesia, euthanasia, or any kind of animal sacrifice is part of the study, include briefly which substances and/or methods were applied<br><br>**Field Research**<br><br>Include the following details if this study involves the collection of plant, animal, or other materials from a natural setting:<br>• Field permit number<br>• Name of the institution or relevant body that granted permission	
**Data Availability**<br><br>Authors are required to make all data underlying the findings described fully available, without restriction, and from the time of publication. PLOS allows rare exceptions to address legal and ethical concerns. See the PLOS Data Policy and FAQ for detailed information.	Yes - all data are fully available without restriction

A Data Availability Statement describing where the data can be found is required at submission. Your answers to this question constitute the Data Availability Statement and **will be published in the article**, if accepted.<br><br>**Important:** Stating 'data available on request from the author' is not sufficient. If your data are only available upon request, select 'No' for the first question and explain your exceptional situation in the text box.<br><br>Do the authors confirm that all data underlying the findings described in their manuscript are fully available without restriction?	
**Describe where the data may be found in full sentences. If you are copying our sample text, replace any instances of XXX with the appropriate details.**<br><br>• If the data are **held or will be held in a public repository**, include URLs, accession numbers or DOIs. If this information will only be available after acceptance, indicate this by ticking the box below. For example: *All XXX files are available from the XXX database (accession number(s) XXX, XXX.*).<br>• If the data are all contained **within the manuscript and/or Supporting Information files**, enter the following: *All relevant data are within the manuscript and its Supporting Information files.*<br>• If neither of these applies but you are able to provide **details of access elsewhere**, with or without limitations, please do so. For example:<br><br>*Data cannot be shared publicly because of [XXX]. Data are available from the XXX Institutional Data Access / Ethics Committee (contact via XXX) for researchers who meet the criteria for access to confidential data.*<br><br>*The data underlying the results presented in the study are available*	All relevant data are within the manuscript

Powered by Editorial Manager® and ProduXion Manager® from Aries Systems Corporation

from (include the name of the third party and contact information or URL).<br>• This text is appropriate if the data are owned by a third party and authors do not have permission to share the data.<br><br>* typeset	
Additional data availability information:	

Manuscript

Click here to access/download;Manuscript;Revised manuscript Plos One.docx

Exploring the photosensitizing potential of nanoliposome loaded improved Toluidine Blue O (NLITBO) against *Streptococcus mutans*: An in-vitro feasibility study

Swagatika Panda[1]*, Lipsa Rout[2], Neeta Mohanty[3], Anurag Satpathy[4], Bhabani sankar satapathy[5], Shakti Rath[6], Divya Gopinath[7,8]*,

1. Department of Oral Pathology and Microbiology, Institute of Dental Sciences, Siksha'O'Anusandhan deemed to be University. Bhubaneswar. Odisha. India. swagatikapanda@soa.ac.in
2. Institute of Dental Sciences, Siksha'O'Anusandhan deemed to be University. Bhubaneswar. Odisha. India. rout.lipsa21@gmail.com
3. Department of Oral Pathology and Microbiology, Institute of Dental Sciences, Siksha'O'Anusandhan deemed to be University. Bhubaneswar. Odisha. India. dr.neetamohanty@gmail.com
4. Department of Periodontics and Implantology, Institute of Dental Sciences, Siksha'O'Anusandhan deemed to be University. Bhubaneswar. Odisha. India. anuragsatpathy@soa.ac.in
5. Professor. School of Pharmaceutical Sciences (SPS), Siksha O Anusandhan Deemed to be University, Kalinga Nagar, Ghatikia, Bhubaneswar. Odisha. India. bhabanisatapathy@soa.ac.in
6. Department of Microbiology & Research, Institute of Dental Sciences, Siksha'O'Anusandhan deemed to be University. India. shaktirathmb@gmail.com
7. Basic medical and Dental Sciences Dept, College of Dentistry. Ajman University, UAE. d.gopinath@ajman.ac.ae
8. Centre of Medical and Bio-Allied Health Sciences and Research, Ajman University, Ajman, UAE

Corresponding author: Swagatika Panda and Divya Gopinath

E-mail: swagatikapanda@soa.ac.in and d.gopinath@ajman.ac.ae

Exploring the photosensitizing potential of nanoliposome loaded improved Toluidine Blue O (NLITBO) against *Streptococcus mutans*: An *in-vitro* feasibility study

Abstract:

Background: *Streptococcus mutans* is a major contributor to dental caries due to its ability to produce acid and survive in biofilms. Microbial resistance towards common antimicrobial agents like chlorhexidine and triclosan has shifted the research towards antimicrobial Photodynamic therapy (PDT). In this context, Toluidine Blue O (TBO) is being explored for its photosensitizing properties against *Streptococcus mutans*. There is a huge variation in the effective concentration of TBO among the current studies owing to the differences in source of and delivery system TBO as well as the time, power and energy densities of light.

Objective: The primary objectives of this study are to encapsulate improved Toluidine Blue O (ITBO) in nanoliposomes (NLITBO), characterize it, and evaluate its antibacterial photosensitizing potential against *Streptococcus mutans* suspensions in vitro.

Method: ITBO was synthesised as per Indian patent (number -543908). NLITBO was prepared using the thin-film hydration method. Dynamic light scattering experiment determined the vesicle size, polydispersity index(PDI), and zeta potential. Surface features were characterized by Scanning and Transmission Electron microscopy. ITBO release from NLITBO was assessed using the extrapolation method. The antibacterial activity of the NLITBO was determined by evaluating the zone of inhibition (ZOI) in the *Streptococcus mutans* culture and comparing with 2% chlorhexidine gluconate. The minimum inhibitory concentration (MIC) of NLITBO as a photosensitizer with red light (wavelength 650nm, power density 0.1 W/cm^2, energy density 9-9.1 J/ cm^2, 90seconds time) was evaluated against *Streptococcus mutans* cells by colorimetric method in 96 well plate.

Results: Percentage drug loading, loading efficiency, yield percentage, vesicle size, PDI, Zeta potential of NLTBO was reported as 9.3±0.4 %, 84.4±7.6%, 73.5%, 123.52 nm, 0.57, -39.54mV respectively. Clusters of uni-lamellar nanovesicles with smooth surfaces with no perforations were observed in SEM and TEM. The size of the vesicle was within 100 nm. At 24 hours, a cumulative 79.81 % of ITBO was released from NLITBO. Mean ZOI and MIC of NLITBO (1 µg /ml) were found to be 0.7±0.2 mm, 0.6µg/ml respectively.

Conclusion:

We have synthesized and encapsulated improved Toluidine Blue O (ITBO) in nanoliposomes (NLITBO) and thoroughly characterized the formulation. The antibacterial efficacy of NLITBO without light was demonstrated by ZOI which is similar to 2% chlorhexidine gluconate. MIC of NLITBO as a photosensitiser along with the optimal light parameter was also proposed in this study. These findings suggested that NLITBO could serve as an effective alternative to conventional antibacterial treatments in managing *Streptococcus mutans* rich biofilms. It can have potential pharmaceutical application in oral health care. Keywords: *Streptococcus mutans*, Toluidine Blue O, nanoliposomes, antibacterial efficacy, biofilm, dental caries

Introduction

Dental caries, the most common infectious oral cavity disease, is a biofilm-mediated progressive disease that results in tooth cavitation.(1) *Streptococcus mutans* is the major cariogenic species in the dental biofilm. Tooth surface adhesion, biofilm formation, acidogenicity through glycolysis, aciduricity, and glucosyltransferase-mediated extracellular polysaccharide synthesis are the cariogenic attributes of *Streptococcus mutans*.(2) Biofilms are complex, structurally organized microbial colonies and extracellular polymeric substances (EPS), a biochemical mixture of polysaccharides, proteins, glycopeptides nucleic acids and lipids. (3) EPS restricts the entry of antimicrobials, and host immune response components, thereby offering protection to microorganisms. The primary challenge for the conventional antibacterial agents such as chlorhexidine and triclosan is penetrating the biofilm.. matrixes in the biofilm absorbs the antibacterial agents, limiting their diffusion and availability within the biofilm interior. (4,5) Furthermore, the small pores EPS impede the passage of larger molecules, (6) necessitating significantly higher antimicrobial doses. Enzymatic inactivation of penetrated antibacterial agents within the biofilm further complicates its effectiveness.(7) Further, the antibacterial effectiveness is challenged by the dormant growth phase of bacteria inside the bio[illegible].(8) Once inside the biofilm, antibacterial agents primarily exert their effects by damaging cell walls or interfering with metabolic processes intracellularly.(9) This triggers the emergence of antimicrobial resistance through various mechanisms, including mutation, acquisition of exogenous resistance genes, horizontal transfer of resistance genes, and efflux pump activity.(8,10) Additionally, the mosaic structure of biofilms generates gradients of oxygen, nutrients, pH, and redox potential, leading to heterogeneity in growth rate and metabolic activity across different biofilm layers, which demand high specificity of antibacterials. (11) Besides microbial resistance, allergic reactions to chlorhexidine (12), ability to form calculus by being incorporated into the dental plaque (13), and increased microleakage (13). These challenges encourage research on exploring alternatives to the existing antimicrobials. Antimicrobial photodynamic therapy (aPDT) has appeared as a non-invasive alternative to modifying cariogenic biofilm.(14,15) where light of appropriate wavelength activates photosensitizers, thus releasing singlet oxygen. This singlet oxygen eventually causes tissue deterioration and cell death through necrosis or apoptosis.

The antibacterial effect of Toluidine Blue O (TBO) as a photosensitizer has been explored in a few studies, (16–20) particularly against gram-positive bacteria like *Streptococcus mutans* due to their cationic and hydrophilic nature. However, there is wide variation in the effective antibacterial concentration of TBO against *Streptococcus mutans*, which owes to inconsistency in the purity of TBO, delivery system of TBO and light parameters. Adequate penetration of the TBO into the biofilm is a noteworthy challenge for antibacterial efficacy, which in turn is dependent upon the purity and delivery system of TBO. Moreover, poor stability and limited bioavailability further limit the photosensitising properties of TBO. To optimize the photosensitising properties of TBO in harnessing the bacterial reduction outcome, we have attempted to minimise the above limitations of TBO-mediated aPDT by synthesising a purer form of TBO (ITBO). Nanoliposomes have been investigated for their biofilm penetrating potential, cell membrane-fusogenicity and biodegradability (10) which simultaneously improves the biocompatibility of the drug-loaded in it. Therefore, further, we attempted to load the ITBO in the core of the nanoliposome (NLITBO). The objective of the present research was to test the antibacterial efficacy of NLITBO against planktonic suspension of clinical strains of *Streptococcus mutans*. The antibacterial activity of NLITBO, when supported by such experiments, could be applied to caries and various other diseases where *Streptococcus mutans* is a primary component of the biofilm. The pharmaceutical industry could harness this technology to develop novel antimicrobial therapies against resistant biofilms, eventually improving patient outcomes and reducing healthcare costs associated with chronic infections.

Methodology:

1. Materials and instrumentations

In addition to the general laboratory equipment, we have used rotary vacuum evaporator, bath sonicator, laboratory lyophilizer, UV/Vis spectrophotometry, zeta sizer, Scanning electron microscope (SEM), and Transmission electron microscope (TEM) for characterization of NLITBO, red LED light source, 96 well plate, and stereomicroscope for in-vivo antibacterial efficacy testing. The list of chemicals, reagents and media are listed in table number 1.

Material	Company	CAS/Catalog number
Improved Toluidine Blue O (ITBO)	Synthesised as mentioned in Indian patent number 543908	NA
Soya-L-α-lecithin (SL)	Sigma-Aldrich	8002-43-5

Cholesterol (CHL)	Sigma-Aldrich	57-88-5
Chloroform	Sigma-Aldrich	67-66-3
Butylated Hydroxyl Toluene (2% w/v)	Sigma-Aldrich	128-37-0
Ethanol	Sigma-Aldrich	64-17-5
Muller Hinton Agar	Himedia	M173
Muller Hinton Broth	Himedia	M391
Nutrient Broth	Himedia	M002
Dimethyl Sulfoxide	Emplura	SA1S70054
Chlorohexidine gluconate	Zodenta	CG-148
Blood Agar Base	Himedia	M073
10X PBS	Himedia	ML023

Table 1 – List of chemicals, reagents, and media used in the study

2. Synthesising Improved Toluidine blue O (ITBO)

ITBO was synthesised by the method as suggested in the Indian patent –(number 543908) dated 01.07.2024**).** (21)

3. Preparation of NLITBO

For the preparation of drug-loaded nanoliposomes, plant-based phospholipids like soya-L-α-lecithin was used as the main lipid component. (22,23) Along with that, cholesterol was also used to stabilize the lipid structure. (24) The experimental nanoliposomes were developed using the thin film hydration method with the required modifications.(25)

Briefly, the amount of soya-L-α-lecithin (SL) and cholesterol (CHL) were dissolved in the required chloroform in a 250 ml round bottom flask. To this mixture, butylated hydroxyl toluene (2% w/v) was added as an antioxidant to prevent oxidation of lipids. The prepared mixture was then subjected to gentle rotation and evaporation of the solvent in a rotary vacuum evaporator (Rotavap, PBU-6, Superfit, Mumbai, India), connected with a water bath at 40 °C. Toluidine blue was added, and a thin film was formed along the inner wall of the round bottom flask. The flask was then kept in a desiccator overnight to remove organic solvent residues still in the thin film. On day 2, the formed thin film was hydrated with phosphate buffer saline (PBS), pH 7.4, for 1 h at 130-150 rpm rotation. Following hydration, the mixture was subjected to sonication in a bath-type sonicator to reduce the size of liposomes to the nanoscale range.

Following sonication, the formulation was kept in a refrigerator overnight at 4 °C. On day 3, the sample was subjected to cold centrifugation at 15,000 rpm for 45 minutes. The supernatant was discarded, and the sediments were collected and stored at –20 °C overnight. The pre-cooled samples were dried in a laboratory lyophilizer for 8-10 hours to obtain the dry powdered product.

4. **Physico-chemical characterization of NLITBO**

a) *Percentage Yield, Percentage drug loading and loading efficiency*: Lyophilized NLITBOs were weighed after each production batch, and the percentage yield was measured as % Yield= (Amount of NLITBOs obtained after lyophilization / Total amount of components used in the formulation batch) × 100. Briefly, the amount of lyophilized NLITBOs was dissolved in ethanol and water in a 7:3 ratio. The sample was then vortexed for 2 min, sonicated for 5 min and centrifuged at 12,000 rpm. The supernatant was examined at 631.0 nm using a UV/VIS spectrophotometer (Beckman, Fullerton, CA, USA). The following formula was calculated for the percentage drug loading and loading efficiency in NLITBOs.

Drug loading (%) = (Amount of TBO in NLITBOs / Amount of NLITBOs obtained) × 100

Drug loading efficiency (%) = (Practical TBO loading/ Theoretical TBO loading) × 100

b) *Determination of average vesicle size, polydispersity index (PDI) and Zeta potential:* To determine the average hydrodynamic size, the PDI and zeta potential of NLTBOs were evaluated using the Zeta sizer (Nano ZS 90, Malvern Instruments Ltd., UK) (*Satapathy et al., 2023*). Briefly, lyophilized NLITBOs were diluted with deionized water, followed by sonication for 5 minutes. At 25 ± 0.5 ºC, the sample was observed using a Zeta sizer.

c) Determination of surface features

The surface morphology and interior structure of the NLITBO nanovesicles were investigated using SEM and TEM, respectively. The lyophilized samples were spread out over a stub on a carbon tape. The sample was then vacuum dried, followed by gold coating over the surface, and was observed under SEM (JSM 6100; JEOL, Tokyo, Japan). NLITBO was suspended in deionized water and put onto the carbon-coated copper grid (mesh), which was then air-dried. The dried sample was then analysed under TEM (Tecnai G2 Spirit BioTwin FP 5018/40, FEI, Hillsboro, USA).

d) ITBO release assessment in vitro

10 mg of lyophilized NLITBOs was suspended in phosphate-buffered saline (PBS, pH of 7.4) and was placed in a dialysis bag (Himedia dialysis membrane-60, Mumbai, India). Two open ends of the dialysis bag were tied together using cotton thread, and the assembly was placed in a beaker filled with 50 millilitres of PBS. The beaker was placed over a magnetic stirrer (rotation speed: 300 rpm), and at fixed intervals, 1 millilitre of the release medium was removed with simultaneous replenishment of fresh PBS. The samples were analysed using a UV-visible spectrophotometer at a fixed wavelength of 631.0 nm. The amount of ITBO released from NLITBOs was determined by the extrapolation method from the standard calibration curve.

e) Bacterial strain and culture condition

Clinical strains of *Streptococcus* [illegible]*tans*, isolated from patients with caries as described previously,(26) were cultured in blood agar (Hi-media) and maintained in the laboratory. The isolation and identification of the bacteria followed the standard microbiological procedure, ensuring the authenticity and clinical relevance of the strains. The strains were cultured in Muller Hinton agar media for experimentation. Bacterial Inoculum was prepared by inoculating *Streptococcus mutans* culture in Muller Hinton broth and incubated until the culture reached the logarithmic growth phase.

f) Zone of inhibition(ZOI)

In vitro, the antibacterial activity of NLITBO was determined by the agar well diffusion method. 0.5 McFarland standard culture (1.5×108 cfu mL−1) of *Streptococcus mutans* was prepared in Muller-Hinton agar media (HI media, India). 100 µL inoculum of *Streptococcus mutans* was spread onto Muller Hinton agar plates using a sterile cotton swab and allowed to dry, and wells were made with a sterile borer in the inoculated agar plates. 1 microgram of lyophilised NLITBO was dissolved in 1 ml of distilled water. 100 µL volumes of each test concentration were propelled into the culture plate of *Streptococcus mutans*. Then, the plate was incubated for 24 h at 37 °C. 10% aqueous Dimethyl sulfoxide (DMSO) was used as the negative control; 2% chlorhexidine was used as the positive control. The antimicrobial activity of NLITBO was observed by recording the ZOI. The experiments were performed in triplicates, and the mean diameter of the ZOI with standard deviation was recorded.

*g) Minimum inhibitory concentration (*MIC*) determination*

The MIC of NLITBO was determined by the colourimetric method in 96 well microtiter plates (Tarsons).(27) A stock solution of NLITBO in distilled water was made. The designing of the 96-well microplate was performed as follows: One column each was designed for positive and negative control. Seven test columns were composed of serial concentrations of NLITBO (0.9, 0.8, 0.7, 0.6, 0.5, 0.4, 0.3 µg/ ml respectively). The microbial culture was inoculated into Nutrient broth. The inoculum density was adjusted to approximately 0.5 McFarland standard (1-2 x 10^8 CFU/mL).100 µl of NLITBO solutions having concentrations ranging from 0.9 µg to 0.3 µg along with 100 µl of the prepared bacterial culture were added in each column of 96 well microtiter plate ensuring the final volume in each well is 200 µL. The plate was covered to prevent contamination and light and incubated at 37°C for 10 minutes. After incubation, each well was exposed to red LED light with a wavelength of 650nm and energy density approximating 9.1 J/cm^2. At the same energy density, the experiment was repeated thrice with three different combinations of power density and time (0.1 W/cm^2 + 90s, 0.08 W/cm^2 + 110s, 0.06 W/cm^2 + 150s). The light was applied to each well using a round tip with an 8 mm diameter attached to the light-emitting device (Novo Duo, Novolase, India). 20 µL of TTC solution (0.2% w/v) was added to each well, including the positive and negative controls. The positive control was without NLITBO, and the negative control was without the bacteria. Wells other than the receiving wells were blocked from light by aluminium foil. The tip of the light device accurately fits the surface of each well, which is approximately 7 mm in diameter, so a uniform sphere of light is applied in the suspension mix. The plate was incubated overnight at room temperature to allow the reduction of TTC by metabolically active microorganisms. The wells were compared to determine the lowest concentration of TBO, which shows no or minimal colour change or formazan formation. The colour change was read visually, and a stereomicroscope (Motic, USA) was used to observe red formazan precipitates in the wells. The experiment was conducted in triplicate, and the mean concentration of TBO at which bacterial growth was inhibited was accepted as the MIC.

h) Statistical tests: Mean of ZOI among four groups were compared by Analysis of Variance (ANOVA) to observe the differences. To assess the variability across repeated experiments we calculated mean and standard deviation of MIC of TBO.

Results

a) Percentage Yield, Percentage drug loading, and Percentage loading efficiency of experimental NLITBOs

The three best formulations were selected from several formulation batches based on their drug-carrying capacity. Further, out of the three reported formulations, NLITBO-2 was found to be suitable (Table 1). The % drug loading for NLITBO-2 was 9.3±0.4 %, whereas 3.6±1.1% and 5.2±1.02 % for NLITBO-1 and NLITBO-3, respectively. NLITBO-2 also showed higher loading efficiency (84.4±7.6%) with yield percentage (73.5%) than the other two formulations (Table 2). Based on these parameters, we selected NLITBO-2 for further studies.

Table 2. Composition and percentage yield, loading and efficiency of three formulations

Formulation code	SL:CHL	ITBO Quantity (mg)	% yield	Practical % loading	% Loading efficiency
NLTBO -1	1:1	15	61.4	3.6±1.1	58.3 ±5.1
NLTBO -2	1:3	15	73.5	9.3±0.4	84.4±7.6
NLTBO -3	1:5	15	56.6	5.2±1.02	61.3 ±0.4

b) Size analysis and zeta potential of selected NLTBOs

DLS data revealed that the selected formulation (NLTBO-2) was well within the desired nano-size range (Z-avg = 123.52 nm) (Fig.1A). The PDI value was found to be 0.57. Zeta potential of NLTBO-2 was reported to be -39.54mV (Fig.1 B).

c) Surface and internal structure analysis

SEM and TEM results depicted the successful formulation of NLITBO within the desired nano-range. All formed nanovesicles were spherical with smooth surfaces (Figure 2A). Most NLTBO-2 had a homogeneous size distribution, and there was a strong correlation between the vesicle sizes and the average size found in the DLS data. TEM study depicted a clear formation of uni-lamellar vesicular structures (Figure 2B). Nanoliposome vesicles with a spherical form, undistorted surface, and darker interior were observed in the TEM image (Figure 2B). The vesicles had no leaks or perforations, and the lamellarity was unbroken. The size of the vesicle was determined to be within 100 nm, which too supported by SEM and DLS data.

d) In vitro drug release study

The in vitro drug release study demonstrated sustained release characteristics of the NLITBO-2 at pH 7.4. At 24 h, a cumulative 79.81 % of ITBO was released from NLITBO-2 (Figure 3).

e*) Zone of inhibition(ZOI)*

Mean ZOI of the positive control, 2% chlorhexidine gluconate, 1 µg/ml NLITBO, 0.5 µg/ml NLITBO, and 0.25 µg/ml NLITBO were 1.2±0.3mm, 0.7±0.2mm, 0.3±0.1mm, and 0 mm respectively. ANOVA test demonstrated no significant difference between the four groups ($p>0.05$).

f) MIC of NLITBO

We observed no colour change in the test well containing 0.6 µg /ml ±0.03) NLITBO, indicating clear inhibition of bacterial growth to a gradual increase in the colour intensity as the concentration of NLITBO decreases. Therefore, we concluded that the MIC of NLITBO is 0.60 µg /ml ±0.03). Both positive and negative controls did not show any change.

Discussion

This experiment has explored the antibacterial efficacy of NLITBO against planktonic suspension of clinical strains of *Streptococcus mutans* , a significant cariogenic species. This study assessed the potential of NLITBO as a photosensitiser in aPDT, focusing on the advantage of nanoliposomes as a delivery system and the purer form of TBO (ITBO) as a photosensitiser. The results showed that NLITBO exhibited significant antibacterial activity against *Streptococcus mutans*. The ZOI produced by 1 µg/ml NLITBO (0.7+0.2 mm) alone without PDT is not significantly different than ZOI produced by 2% chlorhexidine (1.2+0.3 mm) (p value>0.05) but promising enough to warrant further exploration of MIC of aPDT using NLITBO. This indicates that the antimicrobial effect of NLITBO is equivalent to chlorhexidine even without the light activation. This may be attributed to improved stability, improved cellular uptake, membrane disruption, and the synergistic effects of the nanoliposome carrier in NLITBO. Further experiment on antimicrobial effect of NLITBO activated by light can be explored.

The successful synthesis of ITBO and incorporation into the nanoliposome was well characterized by the appropriate composition ratio of soya α lecithin and cholesterol (1:3), high percentage yield (73.5%), drug loading capacity (84.4±7.6), and surface and interior characteristics . Incorporating soya-α-lecithin and cholesterol in 5 : 1 ratio has been found to extend the stability of cinnamon oil for 96 hrs and enhance the anti-biofilm action against

methicillin-resistant staphylococcus aureus (MRSA) by almost 10 times as compared to free cinnamon oil. (28) The lower value of PDI (0.57) indicates a homogenous and narrow distribution pattern of the formulation.(29) Zeta potential of -39.5mV classifies NLITBO as an anionic nanoliposome, which improves the stability in suspension. Higher similar charges between the nanovesicles create strong repulsive forces, keeping vesicles separated for longer. Stable nano formulations have been reported to have a surface charge beyond -30 mV to +30 mV.(30,31) The drug release profile exhibited a controlled release pattern over the study period, which is beneficial for effective clinical application, reducing dose and dose-related side effects. (32) NLITBO showed antibacterial activity even without photoactivation, as observed by mean ZOI in the present study, which implied inherent antibacterial properties of NLITBO that can be further potentiated by red light application. This finding is negated by Shen et al (33) but supported by Nastri et al (34) who demonstrated the antibacterial effect of TBO at 10 µM concentration which was further potentiated by light application. The antibacterial activity of NLITBO without light may be attributed to the improved TBO and coating with anionic nanoliposome. NLITBO, owing to its zeta potential -39.5mV, is an anionic nanoliposome carrying cationic photosensitiser, ITBO. Anionic nanoliposomes may be less effective than cationic liposomes against gram-positive bacteria like *Streptococcus mutans*.(35) However, upon activation of the cationic photosensitiser, (ref) ITBO may penetrate the dental biofilm and *Streptococcus mutans* cell wall more effectively than cationic nanoliposome carrying cationic ITBO as it may not penetrate biofilm well due to the overall charge repulsion. Studies suggested that nanoliposomes loaded with cationic photosensitizers are designed for hydrophobic interaction with the negatively charged surface of gram-positive bacterial cells. This allows for the diffusion of nanoliposome-loaded photosensitisers into *Streptococcus mutans*. (36) The liposome-bacteria fusion was shown to enhance the diffusion of the drugs into *Pseudomonas aeruginosa*. (37,38) This selective targeting enhances the specificity and efficacy of aPDT against gram-positive pathogens. Upon exposure to red light, the photosensitizer, ITBO loaded within the nanoliposomes, becomes excited. (39) It undergoes photochemical reactions, leading to the generation of reactive oxygen species (ROS), such as singlet oxygen and free radicals. (40) The generated ROS exert their antimicrobial effects by inducing oxidative damage to various cellular components of the gram-positive bacterial cells, eventually disrupting cellular functions and promoting cell death. This disruption facilitates the entry of ROS into the bacterial cytoplasm, enhancing their antimicrobial activity. Moreover, sustained release characteristics of the NLITBO-2, as found in this study, shall prove beneficial as there would be a consistent therapeutic effect even in the presence of saliva as the experiment

was conducted at pH 7.4. A significant portion of ITBO (79.81 %) was released from NLITBO-2 by the end of 24 hours. This attribute can be advantageous in a targeted and sustained approach during aPDT against gram-positive bacteria, providing a promising strategy for combating bacterial infections while minimising the risk of antimicrobial resistance and enhancing patient compliance.

This study has identified MIC of NLITBO against clinical strains of *Streptococcus mutans* suspension with red light (wavelength – 650 nm, power density - 0.1mW/cm^2 for 90 seconds). Effective antimicrobial activity of TBO in *Streptococcus mutans* suspension was observed in the concentration range of 0.01 to 3.08 mg/ml. (16–20,41–43) the effective concentration for anti-biofilm activity in *Streptococcus mutans* was 0.1mg/ml (20,44–46) and 41.067 mg/ml. (47) (45,46,48,49) Although the effective antibacterial concentration should vary according to the nature of bacterial culture, there is a huge difference in the effective concentration of TBO in studies on bacterial suspensions, too. (16–20,41–43) This variation may be attributed to the difference in strains of s mutans, light parameters such as energy density, power density, incubation time and time of red-light exposure across studies. Energy densities reported in different studies ranged from 9J/cm2 to 180J/cm2 (19,26,33,35–38), while power densities ranged from 100 mW to 500 mW. Duration of light exposure also varied from 30 seconds (50) to 12 minutes 50 seconds (18). Except for one (39) rest of the studies have used the commercial strain. In general, clinical strains exhibit more genetic diversity, and the findings on clinical strains are applicable in clinical settings. Therefore, the present result can find a broader applicability. There are two clinical studies (27,40) which evaluated the antimicrobial potential of TBO in children with early childhood caries but failed to significantly reduce the bacterial count of the saliva. This contradicting result could be due to the absence of an appropriate drug delivery system. Nanoliposomal formulation, an effective drug delivery system, is known to enhance the bioavailability and biodistribution of the loaded drug.(51) Afrasiabi et al. (52) have shown nanoliposomes' enhanced antimicrobial photodynamic therapy containing curcumin and doxycycline against *Aggregatibacter actinomycetemcomitans*. Therefore, we, for the first time, have emphasised the nanoliposome encapsulation of ITBO and demonstrated that NLITBO is an effective antibacterial agent against clinical strains of *Streptococcus mutans* under optimal red-light parameters (wavelength 650nm, power density 0.1 W, energy density 9-9.1 J, 90seconds time) with a MIC of 0.6µg/ml. These conditions are effective, and the biocompatible concentration of NLITBO and light energy dosage are selected to target the 24-hour incubated bacterial suspension of *Streptococcus mutans.*

Firstly, our study demonstrated the minimum inhibitory concentration (MIC) of NLITBO. The observed MIC of 0.60 μg/mL for NLITBO indicates its potent bactericidal activity, highlighting its potential as a promising candidate for caries prevention. Supporting this previous study (19) has also shown better antibiofilm action of TBO-silver nanoparticle conjugates on *Streptococcus mutans* than TBO alone. TBO has also been added with microbial efflux pump inhibitors for successful biofilm inactivation. (53) The enhanced efficacy of NLITBO can be attributed to several factors. One plausible explanation is the improved bioavailability and targeted delivery of TBO facilitated by nanoliposomes. Nanoliposomes offer a stable and controlled release platform, allowing for prolonged exposure of TBO to bacterial cells, thus maximizing its antimicrobial effects and minimizing potential adverse effects on surrounding tissues. While the conventional antimicrobials often struggle to penetrate the extracellular polymeric substance barrier in the biofilm to effectively target bacteria, nanoparticles' (NP) ability to penetrate the biofilm can inhibit the biofilm formation. (54) NPs can exert the antibacterial activity through different mechanisms such as cell wall disruption, interaction with DNA/RNA or proteins, biofilm inhibition, or ROS generation. (55) There are three stages during the interaction of NP with biofilm. NP transported to the surface of the biofilm, gets attached to the biofilm followed by migration in the biofilm. Several factors such as physicochemical properties of NPs and extracellular matrix influence how the individual phases take place. (56) Additionally, the nanoliposomes get attached to the cell wall of bacteria and enhance ITBO penetration into the bacterial cytoplasm, where it gets photoactivated and the nascent oxygen released causes bacterial death. Furthermore, the nanoliposome carrier system may protect ITBO from degradation and enhance its retention within the oral cavity, prolonging its therapeutic effects.

Despite the promising findings of our study, several limitations should be considered. Firstly, our study was conducted in vitro, and further investigations are needed to assess the efficacy of NLITBO in vivo. Additionally, while our study focused on *Streptococcus mutans* , future research should explore its effects on other oral pathogens and its potential interactions with the oral microbiome. Furthermore, the safety profile

of NLITBO, including potential cytotoxicity and adverse effects, warrants thorough evaluation before clinical translation. The sensitivity of planktonic bacteria to antimicrobial agents is far lower than biofilm-grown bacteria.(57) Therefore, further research may be directed to find out the biofilm inhibitory concentration and biofilm microbicidal concentration, which would effectively explore the efficacy, safety, and practical application of ITBO in the context of

dental care to determine its role in preventing and managing infections associated with *Streptococcus mutans* .

In conclusion, our study demonstrates the enhanced antimicrobial efficacy of NLITBO and the MIC with optimum light parameters during aPDT against *Streptococcus mutans*. This research's purity and drug delivery system may have clinical benefits when exploring caries preventive agents. Further research is needed to validate our findings in vivo, assess safety profiles, and explore potential applications in clinical practice. Overall, NLITBO represents an innovative and promising approach to caries prevention, addressing critical challenges in antimicrobial therapy and paving the way for improved oral health outcomes.

Acknowledgement: Nil

Funding : The authors did not receive any funding for the conduct of this research. The APC is funded by Ajman University

Conflict of Interest: None to declare

Figure Legends

Figure 1: DLS demonstrating Size (A) and Zeta potential (B) of NLITBO

Figure 2: Surface structure in SEM (A) and interior structure in TEM (B) of NLITBO

Figure 3: In vitro release behaviour of NLITBO in PBS, pH 7.4

Figure 4: Zone of Inhibition 1. NLITBO (0.25 µg /µl) - no zone; 2. NLITBO (0.5 µg /ml) - 0.3±0.1 mm.; 3. NLITBO (1 µg /µl) - 0.7±0.2 mm ; 4. Negative Control - no zone; 5; Chlorhexidine gluconate 2% - 1.2 ± 0.3 mm

Table legends

Table 1 – List of chemicals, reagents and media used in the study.

Table 2 – Composition and percentage yield, loading and efficiency of three formulations

References

1. Marcenes W, Kassebaum NJ, Bernabé E, Flaxman A, Naghavi M, Lopez A, et al. Global burden of oral conditions in 1990-2010: a systematic analysis. J Dent Res. 2013 Jul;92(7):592–7.

2. Lynch DJ, Michalek SM, Zhu M, Drake D, Qian F, Banas JA. Cariogenicity of Streptococcus mutans Glucan-Binding Protein Deletion Mutants. Oral Health Dent Manag [Internet]. 2013 Dec [cited 2024 Apr 22];12(4):191–9. Available from: https://www.ncbi.nlm.nih.gov/pmc/articles/PMC4437697/

3. Chávez de Paz LE, Resin A, Howard KA, Sutherland DS, Wejse PL. Antimicrobial effect of chitosan nanoparticles on streptococcus mutans biofilms. Appl Environ Microbiol. 2011 Jun;77(11):3892–5.

4. Gupta TT, Karki SB, Fournier R, Ayan H. Mathematical Modelling of the Effects of Plasma Treatment on the Diffusivity of Biofilm. Appl Sci [Internet]. 2018 Oct [cited 2024 Apr 17];8(10):1729. Available from: https://www.mdpi.com/2076-3417/8/10/1729

5. Oliveira M, Brugnera D, Piccoli R. Biofilms in the dairy industries: general aspects and the use of essential oils as a new control alternative. Rev Inst Laticínios Cândido Tostes. 2013 Feb 1;68:65–73.

6. Gilbert P, Allison DG, McBain AJ. Biofilms in vitro and in vivo: do singular mechanisms imply cross-resistance? J Appl Microbiol. 2002;92 Suppl:98S-110S.

7. Albayaty YN, Thomas N, Hasan S, Prestidge CA. Penetration of topically used antimicrobials through *Staphylococcus aureus* biofilms: A comparative study using different models. J Drug Deliv Sci Technol [Internet]. 2018 Dec 1 [cited 2024 Apr 17];48:429–36. Available from: https://www.sciencedirect.com/science/article/pii/S1773224718308256

8. Smith AW. Biofilms and antibiotic therapy: is there a role for combating bacterial resistance by the use of novel drug delivery systems? Adv Drug Deliv Rev. 2005 Jul 29;57(10):1539–50.

9. Neville N, Jia Z. Approaches to the Structure-Based Design of Antivirulence Drugs: Therapeutics for the Post-Antibiotic Era. Mol Basel Switz. 2019 Jan 22;24(3):378.

10. Dos Santos Ramos MA, Da Silva PB, Spósito L, De Toledo LG, Bonifácio BV, Rodero CF, et al. Nanotechnology-based drug delivery systems for control of microbial biofilms: a review. Int J Nanomedicine. 2018;13:1179–213.

11. Stewart PS, Franklin MJ. Physiological heterogeneity in biofilms. Nat Rev Microbiol. 2008 Mar;6(3):199–210.

12. Pemberton MN, Gibson J. Chlorhexidine and hypersensitivity reactions in dentistry. Br Dent J. 2012 Dec;213(11):547–50.

13. Zanatta FB, Antoniazzi RP, Rösing CK. Staining and calculus formation after 0.12% chlorhexidine rinses in plaque-free and plaque covered surfaces: a randomized trial. J Appl Oral Sci Rev FOB. 2010;18(5):515–21.

14. Soukos NS, Goodson JM. Photodynamic therapy in the control of oral biofilms. Periodontol 2000 [Internet]. 2011 [cited 2024 Apr 19];55(1):143–66. Available from: https://onlinelibrary.wiley.com/doi/abs/10.1111/j.1600-0757.2010.00346.x

15. Gursoy H, Ozcakir-Tomruk C, Tanalp J, Yilmaz S. Photodynamic therapy in dentistry: a literature review. Clin Oral Investig. 2013 May;17(4):1113–25.

16. Aragão MGB, Costa CAG de A, Lima RA, Rodrigues LKA, Duarte S, Zanin ICJ. Comparative Effect of Two Red Lights on Streptococcus mutans Biofilms and Assessment of Temperature Variances in Human Teeth During In Vitro Photodynamic Antimicrobial Chemotherapy. Photobiomodulation Photomed Laser Surg. 2019 Jan;37(1):31–7.

17. Araújo PV, Teixeira KIR, Lanza LD, Cortes ME, Poletto LTA. In vitro lethal photosensitization of S. mutans using methylene blue and toluidine blue O as photosensitizers. Acta Odontol Latinoam AOL. 2009;22(2):93–7.

18. Misba L, Zaidi S, Khan AU. Efficacy of photodynamic therapy against Streptococcus mutans biofilm: Role of singlet oxygen. J Photochem Photobiol B. 2018 Jun;183:16–21.

19. Misba L, Kulshrestha S, Khan AU. Antibiofilm action of a toluidine blue O-silver nanoparticle conjugate on Streptococcus mutans: a mechanism of type I photodynamic therapy. Biofouling. 2016;32(3):313–28.

20. Zanin ICJ, Lobo MM, Rodrigues LKA, Pimenta LAF, Höfling JF, Gonçalves RB. Photosensitization of in vitro biofilms by toluidine blue O combined with a light-emitting diode. Eur J Oral Sci. 2006 Feb;114(1):64–9.

21. Panda S. SYNTHESIS OF TOLUIDINE BLUE O AND A KIT FOR MUCOSAL APPLICATION. 543908, 2024.

22. Le NTT, Cao VD, Nguyen TNQ, Le TTH, Tran TT, Hoang Thi TT. Soy Lecithin-Derived Liposomal Delivery Systems: Surface Modification and Current Applications. Int J Mol Sci. 2019 Sep 23;20(19):4706.

23. van Hoogevest P, Wendel A. The use of natural and synthetic phospholipids as pharmaceutical excipients. Eur J Lipid Sci Technol EJLST. 2014 Sep;116(9):1088–107.

24. Briuglia ML, Rotella C, McFarlane A, Lamprou DA. Influence of cholesterol on liposome stability and on in vitro drug release. Drug Deliv Transl Res. 2015 Jun;5(3):231–42.

25. TAMADDON L, MOHAMADI N, BAVARSAD N. Preparation and Characterization of Mucoadhesive Loratadine Nanoliposomes for Intranasal Administration. Turk J Pharm Sci. 2021 Aug;18(4):492–7.

26. Mohanty M, Govind S, Rath S. Prevalence of salivary microbial load and lactic acid presence in diabetic and non-diabetic individuals with different dental caries stages. Restor Dent Endod. 2024 Feb;49(1):e4.

27. Barnes V L, Heithoff DM, Mahan SP, House JK, Mahan MJ. Antimicrobial susceptibility testing to evaluate minimum inhibitory concentration values of clinically relevant antibiotics. STAR Protoc [Internet]. 2023 Aug 10 [cited 2024 May 14];4(3):102512. Available from: https://www.ncbi.nlm.nih.gov/pmc/articles/PMC10448204/

28. Cui H, Li W, Li C, Vittayapadung S, Lin L. Liposome containing cinnamon oil with antibacterial activity against methicillin-resistant Staphylococcus aureus biofilm. Biofouling. 2016;32(2):215–25.

29. Salah S, Awad GEA, Makhlouf AIA. Improved vaginal retention and enhanced antifungal activity of miconazole microsponges gel: Formulation development and in vivo therapeutic efficacy in rats. Eur J Pharm Sci Off J Eur Fed Pharm Sci. 2018 Mar 1;114:255–66.

30. Satapathy BS, Biswal B, Pattnaik S, Parida R, Sahoo RN. A mucoadhesive nanolipo gel containing Aegle marmelos gum to enhance transdermal effectiveness of linezolid for vaginal infection: In vitro evaluation, in vitro-ex vivo correlation, pharmacokinetic studies. Int J Pharm. 2023 Dec 15;648:123542.

31. Satapathy BS, Kumar LA, Pattnaik G, Barik B. Lomustine Incorporated Lipid Nanostructures Demonstrated Preferential Anticancer Properties in C6 Glioma Cell Lines with Enhanced Pharmacokinetic Profile in Mice. Acta Chim Slov. 2021 Dec 15;68(4):970–82.

32. Maiti S, Sen KK, Maiti S, Sen KK. Introductory Chapter: Drug Delivery Concepts. In: Advanced Technology for Delivering Therapeutics [Internet]. IntechOpen; 2017 [cited 2024 Aug 17]. Available from: https://www.intechopen.com/chapters/54825

33. Shen J, Liang Q, Su G, Zhang Y, Wang Z, Baudouin C, et al. In Vitro Effect of Toluidine Blue Antimicrobial Photodynamic Chemotherapy on Staphylococcus epidermidis and Staphylococcus aureus Isolated from Ocular Surface Infection. Transl Vis Sci Technol [Internet]. 2019 Jun 21 [cited 2024 May 14];8(3):45. Available from: https://www.ncbi.nlm.nih.gov/pmc/articles/PMC6590089/

34. Nastri L, Donnarumma G, Porzio C, De Gregorio V, Tufano MA, Caruso F, et al. Effects of Toluidine Blue-Mediated Photodynamic Therapy on Periopathogens and Periodontal Biofilm: In Vitro Evaluation. Int J Immunopathol Pharmacol [Internet]. 2010 Oct 1 [cited 2024 May 14];23(4):1125–32. Available from: https://doi.org/10.1177/039463201002300416

35. Drulis-Kawa Z, Dorotkiewicz-Jach A, Gubernator J, Gula G, Bocer T, Doroszkiewicz W. The interaction between Pseudomonas aeruginosa cells and cationic PC:Chol:DOTAP liposomal vesicles versus outer-membrane structure and envelope properties of bacterial cell. Int J Pharm. 2009 Feb 9;367(1–2):211–9.

36. Beaulac C, Sachetelli S, Lagace J. In-vitro bactericidal efficacy of sub-MIC concentrations of liposome-encapsulated antibiotic against gram-negative and gram-positive bacteria. J Antimicrob Chemother. 1998 Jan;41(1):35–41.

37. Sachetelli S, Khalil H, Chen T, Beaulac C, Sénéchal S, Lagacé J. Demonstration of a fusion mechanism between a fluid bactericidal liposomal formulation and bacterial cells. Biochim Biophys Acta. 2000 Feb 15;1463(2):254–66.

38. Mugabe C, Halwani M, Azghani AO, Lafrenie RM, Omri A. Mechanism of Enhanced Activity of Liposome-Entrapped Aminoglycosides against Resistant Strains of Pseudomonas aeruginosa. Antimicrob Agents Chemother [Internet]. 2006 Jun [cited 2024 Apr 9];50(6):2016–22. Available from: https://www.ncbi.nlm.nih.gov/pmc/articles/PMC1479138/

39. Najm M, Pourhajibagher M, Badirzadeh A, Razmjou E, Alipour M, Khoshmirsafa M, et al. Photodynamic Therapy Using Toluidine Blue O (TBO) Dye as a Photosensitizer against Leishmania major. Iran J Public Health. 2021 Oct;50(10):2111–20.

40. Oleinick NL, Evans HH. The photobiology of photodynamic therapy: cellular targets and mechanisms. Radiat Res. 1998 Nov;150(5 Suppl):S146-156.

41. Vahabi S, Fekrazad R, Ayremlou S, Taheri S, Zangeneh N. The effect of antimicrobial photodynamic therapy with radachlorin and toluidine blue on streptococcus mutans: an in vitro study. J Dent Tehran Iran. 2011;8(2):48–54.

42. Fekrazad R, Seraj B, Chiniforush N, Rokouei M, Mousavi N, Ghadimi S. Effect of antimicrobial photodynamic therapy on the counts of salivary Streptococcus mutans in children with severe early childhood caries. Photodiagnosis Photodyn Ther. 2017 Jun;18:319–22.

43. Hakimiha N, Khoei F, Bahador A, Fekrazad R. The susceptibility of Streptococcus mutans to antibacterial photodynamic therapy: a comparison of two different photosensitizers and light sources. J Appl Oral Sci Rev FOB. 2014 Apr;22(2):80–4.

44. de Sousa Farias SS, Nemezio MA, Corona S a. M, Aires CP, Borsatto MC. Effects of low-level laser therapy combined with toluidine blue on polysaccharides and biofilm of Streptococcus mutans. Lasers Med Sci. 2016 Jul;31(5):1011–6.

45. Balhaddad AA, AlQranei MS, Ibrahim MS, Weir MD, Martinho FC, Xu HHK, et al. Light Energy Dose and Photosensitizer Concentration Are Determinants of Effective Photo-Killing against Caries-Related Biofilms. Int J Mol Sci. 2020 Oct 15;21(20):7612.

46. Teixeira AH, Pereira ES, Rodrigues LKA, Saxena D, Duarte S, Zanin ICJ. Effect of photodynamic antimicrobial chemotherapy on in vitro and in situ biofilms. Caries Res. 2012;46(6):549–54.

47. de Oliveira FS, Cruvinel T, Cusicanqui Méndez DA, Dionísio EJ, Rios D, Machado MAAM. The *in vitro* effect of Antimicrobial Photodynamic Therapy on dental microcosm biofilms from partially erupted permanent molars: A pilot study. Photodiagnosis Photodyn Ther [Internet]. 2018 Mar 1 [cited 2024 Apr 10];21:163–7. Available from: https://www.sciencedirect.com/science/article/pii/S1572100017304076

48. Shirato M, Nakamura K, Kanno T, Lingström P, Niwano Y, Örtengren U. Time-kill kinetic analysis of antimicrobial chemotherapy based on hydrogen peroxide photolysis against Streptococcus mutans biofilm. J Photochem Photobiol B. 2017 Aug;173:434–40.

49. Zanin ICJ, Gonçalves RB, Junior AB, Hope CK, Pratten J. Susceptibility of Streptococcus mutans biofilms to photodynamic therapy: an in vitro study. J Antimicrob Chemother. 2005 Aug;56(2):324–30.

50. Fekrazad R, Khoei F, Hakimiha N, Bahador A. Photoelimination of Streptococcus mutans with two methods of photodynamic and photothermal therapy. Photodiagnosis Photodyn Ther. 2013 Dec;10(4):626–31.

51. Fatfat Z, Karam M, Maatouk B, Fahs D, Gali-Muhtasib H. Chapter 7 - Nanoliposomes as safe and efficient drug delivery nanovesicles. In: Nayak AK, Hasnain MS, Laha B, Maiti S, editors. Advanced and Modern Approaches for Drug Delivery [Internet]. Academic Press; 2023 [cited 2024 Apr 8]. p. 159–97. Available from: https://www.sciencedirect.com/science/article/pii/B9780323916684000022

52. Afrasiabi S, Partoazar A, Chiniforush N. In vitro study of nanoliposomes containing curcumin and doxycycline for enhanced antimicrobial photodynamic therapy against Aggregatibacter actinomycetemcomitans. Sci Rep [Internet]. 2023 Jul 18 [cited 2024 Apr 8];13(1):11552. Available from: https://www.nature.com/articles/s41598-023-38812-4

53. Kishen A, Upadya M, Tegos GP, Hamblin MR. Efflux pump inhibitor potentiates antimicrobial photodynamic inactivation of Enterococcus faecalis biofilm. Photochem Photobiol. 2010;86(6):1343–9.

54. Mohanta YK, Chakrabartty I, Mishra AK, Chopra H, Mahanta S, Avula SK, et al. Nanotechnology in combating biofilm: A smart and promising therapeutic strategy. Front Microbiol. 2022;13:1028086.

55. Wang Y. Liposome as a delivery system for the treatment of biofilm-mediated infections. J Appl Microbiol. 2021;131(6):2626–39.

56. Shkodenko L, Kassirov I, Koshel E. Metal Oxide Nanoparticles Against Bacterial Biofilms: Perspectives and Limitations. Microorganisms. 2020 Oct;8(10):1545.

57. Lindhe J, Karring T, Lang NP. Clinical periodontology and implant dentistry. 4th ed. Oxford, UK: Blackwell; 2003.

Figure 1

Click here to access/download;Figure;Figure 1.png

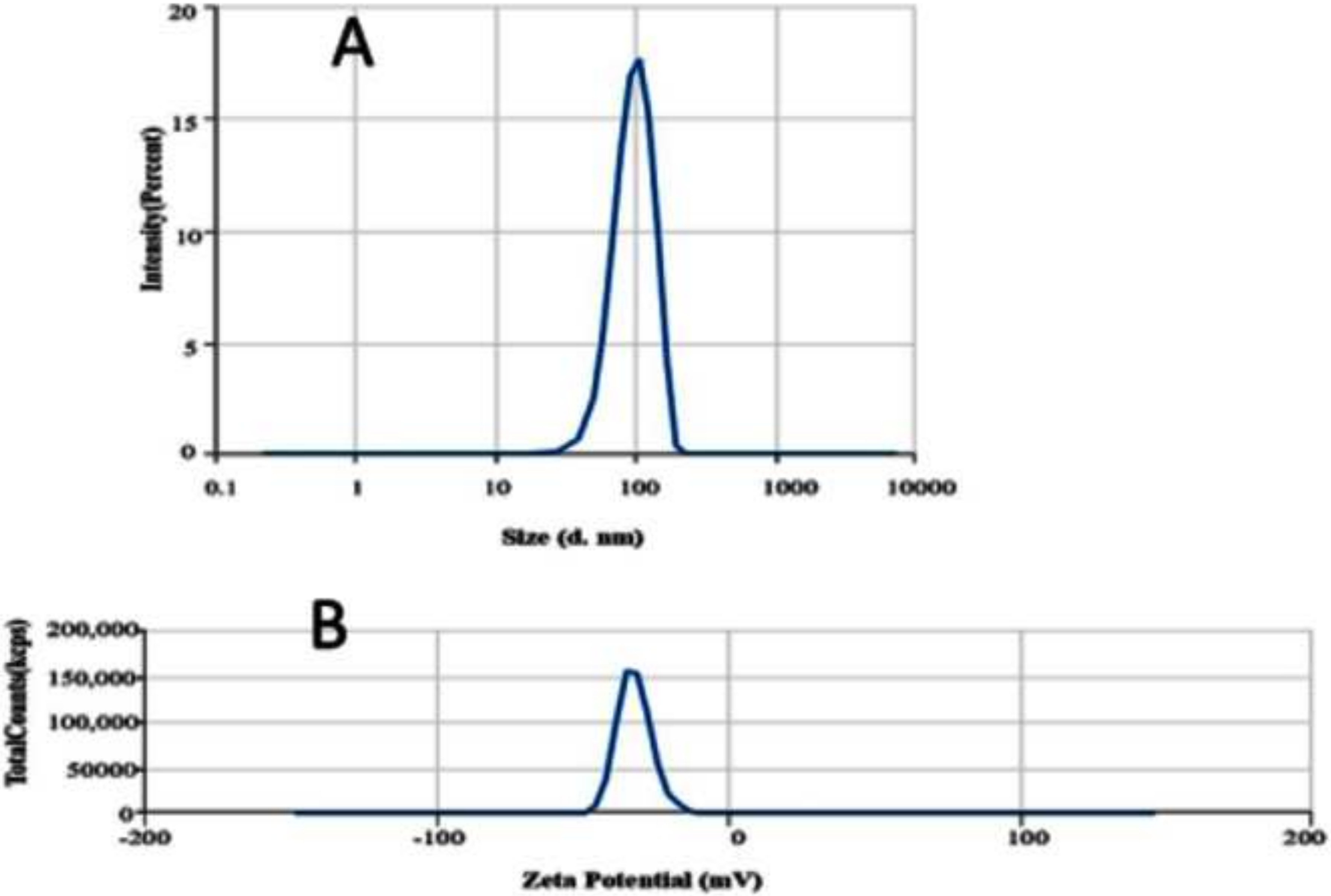

Figure 2

Click here to access/download;Figure;Figure 2.png

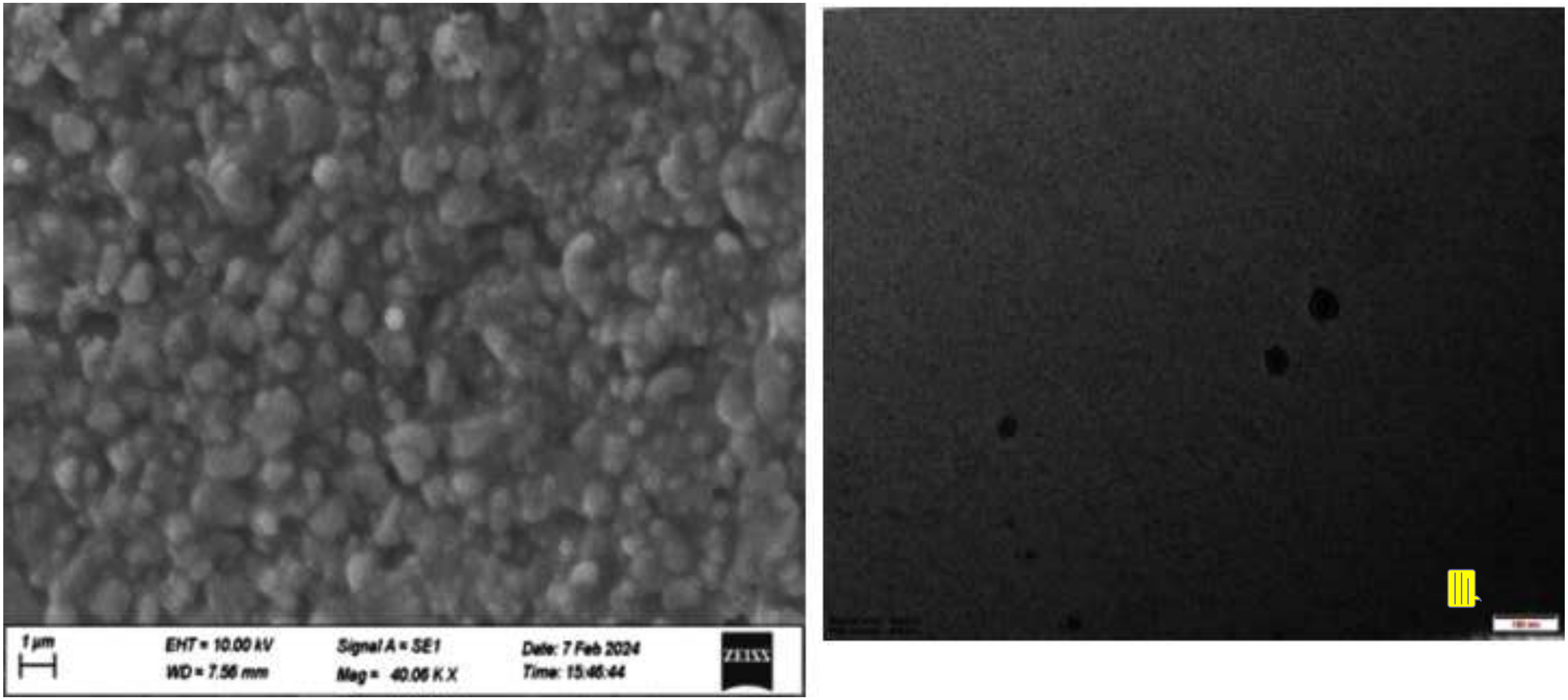

Figure 3

Click here to access/download;Figure;Figure 3.png

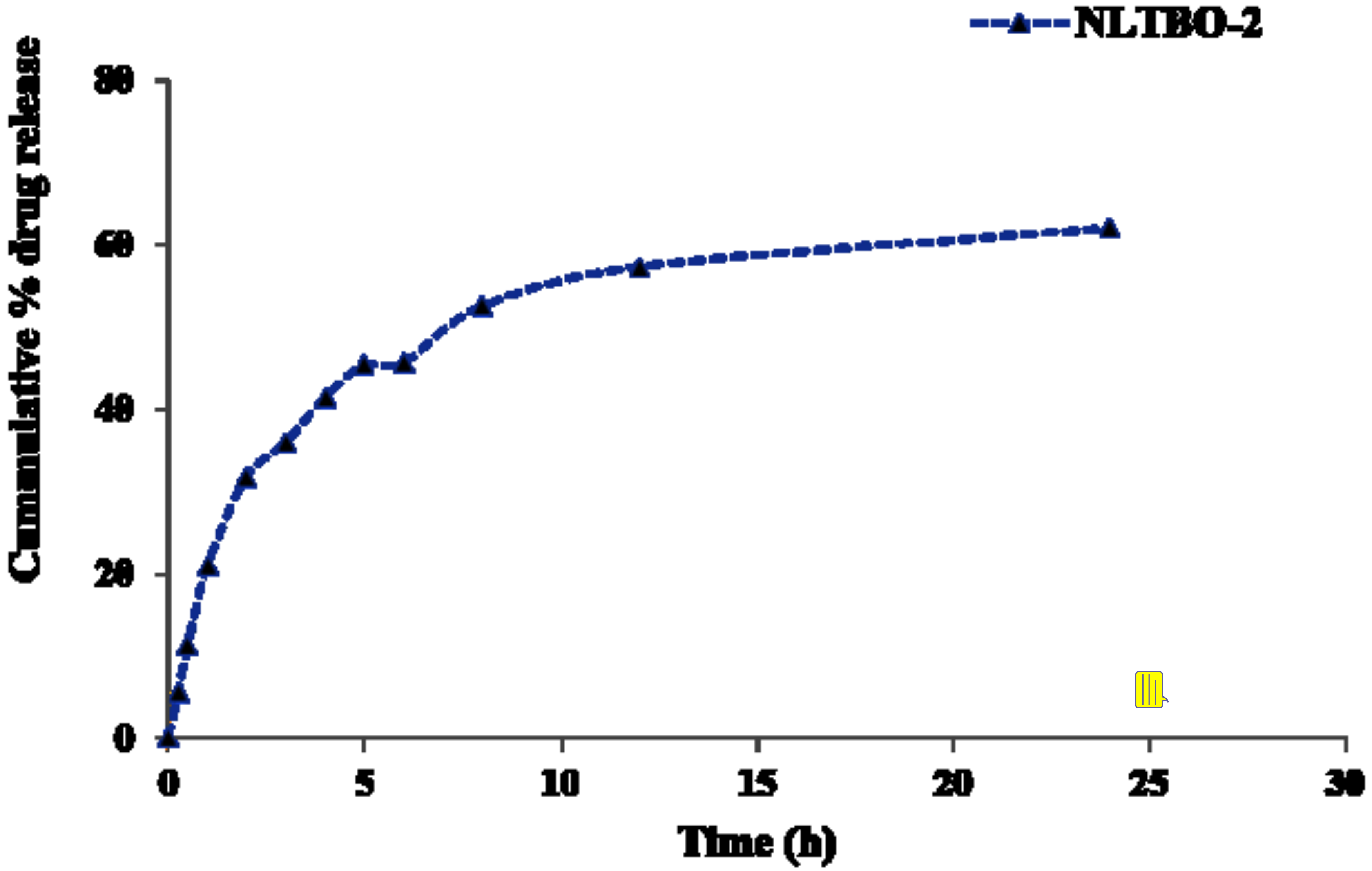

Figure 4

Click here to access/download;Figure;Figure 4.jpg

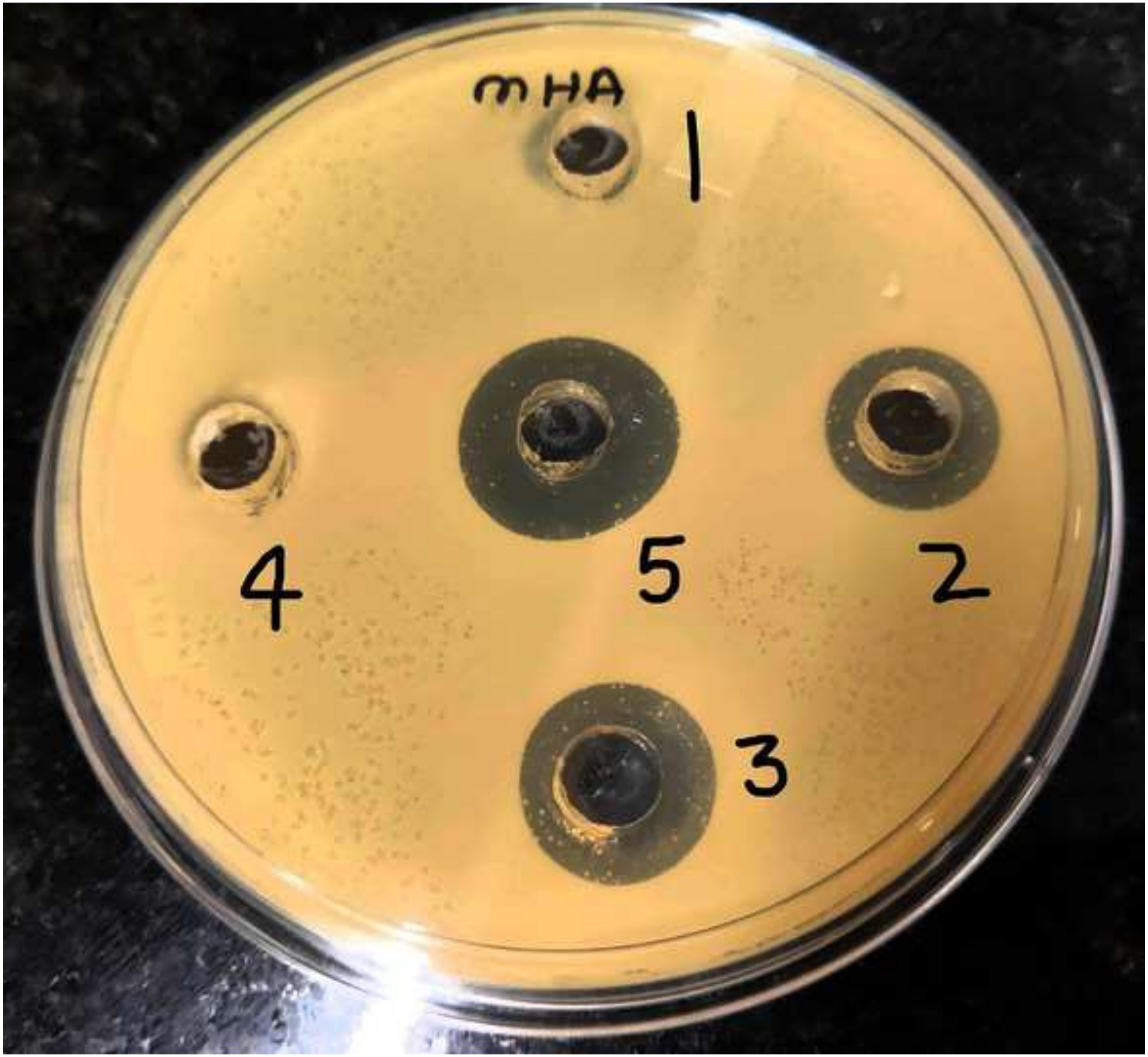

Exploring the photosensitizing potential of nanoliposome loaded improved Toluidine Blue O (NLITBO) against *Streptococcus mutans*: An in-vitro feasibility study

Swagatika Panda[1]*, Lipsa Rout[2], Neeta Mohanty[3], Anurag Satpathy[4], Bhabani sankar satapathy[5], Shakti Rath[6], Divya Gopinath[7,8]*,

1. Department of Oral Pathology and Microbiology, Institute of Dental Sciences, Siksha'O'Anusandhan deemed to be University. Bhubaneswar. Odisha. India. swagatikapanda@soa.ac.in
2. Institute of Dental Sciences, Siksha'O'Anusandhan deemed to be University. Bhubaneswar. Odisha. India. rout.lipsa21@gmail.com
3. Department of Oral Pathology and Microbiology, Institute of Dental Sciences, Siksha'O'Anusandhan deemed to be University. Bhubaneswar. Odisha. India. dr.neetamohanty@gmail.com
4. Department of Periodontics and Implantology, Institute of Dental Sciences, Siksha'O'Anusandhan deemed to be University. Bhubaneswar. Odisha. India. anuragsatpathy@soa.ac.in
5. Professor. School of Pharmaceutical Sciences (SPS), Siksha O Anusandhan Deemed to be University, Kalinga Nagar, Ghatikia, Bhubaneswar. Odisha. India. bhabanisatapathy@soa.ac.in
6. Department of Microbiology & Research, Institute of Dental Sciences, Siksha'O'Anusandhan deemed to be University. India. shaktirathmb@gmail.com
7. Basic medical and Dental Sciences Dept, College of Dentistry. Ajman University, UAE. d.gopinath@ajman.ac.ae
8. Centre of Medical and Bio-Allied Health Sciences and Research, Ajman University, Ajman, UAE

Corresponding author: Swagatika Panda and Divya Gopinath

E-mail: swagatikapanda@soa.ac.in and d.gopinath@ajman.ac.ae

Exploring the photosensitizing potential of nanoliposome loaded improved Toluidine Blue O (NLITBO) against *Streptococcus mutans*: An *in-vitro* feasibility study

Abstract:

Background: *Streptococcus mutans* is a major contributor to dental caries due to its ability to produce acid and survive in biofilms. Microbial resistance towards common antimicrobial agents like chlorhexidine and triclosan has shifted the research towards antimicrobial Photodynamic therapy (PDT). In this context, Toluidine Blue O (TBO) is being explored for its photosensitizing properties against *Streptococcus mutans*~~*S. mutans*~~. There is a huge variation in the effective concentration of TBO among the current studies owing to the differences in source of and delivery system TBO as well as the time, power and energy densities of light.

Objective: The primary objectives of this study are to encapsulate improved Toluidine Blue O (ITBO) in nanoliposomes (NLITBO), characterize it, and evaluate its antibacterial photosensitizing potential against *Streptococcus mutans* suspensions in vitro. ~~Hypothesizing the better antibacterial effectiveness, we have synthesised and characterised the improved TBO (ITBO) encapsulated in nanoliposomes. Further, we evaluated the zone of inhibition and Minimum inhibitory concentration (MIC) of nanoliposome-loaded improved TBO (NLITBO) against~~ ~~*Streptococcus mutans*~~ ~~*S. mutans* suspensions in vitro.~~

Method: ITBO was synthesised as per Indian patent ~~application number~~ (number - 543908)~~(202131053258 A dtd 26.05.2023)~~. NLITBO was prepared using the thin-film hydration method.~~, and a~~ D~~d~~ynamic light scattering experiment determined the vesicle size, polydispersity index(PDI), and zeta potential. Surface features were characterized by Scanning and Transmission Electron microscopy. ITBO release from NLITBO was assessed using the extrapolation method. The antibacterial activity of the NLITBO was determined by evaluating the zone of inhibition (ZOI) in the *Streptococcus mutans* ~~*S. mutans*~~ culture and comparing with 2% chlorhexidine gluconate. The minimum inhibitory concentration (MIC) of NLITBO as a photosensitizer with red light (wavelength 650nm, power density 0.1 W/cm^2, energy density 9-9.1 J/ cm^2, 90seconds time) was evaluated against *Streptococcus mutans* ~~*S. mutans*~~ cells by ~~colourimetric~~colorimetric method in 96 well plate.

Results: Percentage drug loading, loading efficiency, yield percentage, vesicle size, ~~polydispersity index~~ (PDI~~) value~~, Zeta potential of NLTBO was reported as 9.3±0.4 %, 84.4±7.6%, 73.5%, 123.52 nm, 0.57, -39.54mV respectively. Clusters of uni-lamellar nanovesicles with smooth surfaces with no perforations were observed in SEM and TEM. The size of the vesicle was within 100 nm. At 24 hours, a cumulative 79.81 % of ITBO was released

from NLITBO. Mean ZOI and MIC of NLITBO (1 µg /ml) were found to be~~as~~ 0.7±0.2 mm, ~~and MIC was~~ 0.6µg/ml respectively.

Conclusion: ~~For the first time, we have evaluated the antibacterial efficacy of NLITBO against *Streptococcus mutans* *S. mutans* with a special focus on improvising the purity of TBO (ITBO) by a unique synthesis method and the delivery of ITBO by encapsulating in the nanoliposome. We have determined the MIC of NLITBO and optimized the light parameters. This would facilitate consistencies in further similar research and ease the clinical translation of aPDT using NLITBO to prevent and treat dental caries.~~

We have synthesized and encapsulated improved Toluidine Blue O (ITBO) in nanoliposomes (NLITBO) and thoroughly characterized the formulation. The antibacterial efficacy of NLITBO without light was demonstrated by ZOI which is similar to 2% chlorhexidine gluconate. MIC of NLITBO as a photosensitiser along with the optimal light parameter was also proposed in this study. These findings suggested that NLITBO could serve as an effective alternative to conventional antibacterial treatments in managing *Streptococcus mutans* rich biofilms. It can have potential pharmaceutical application in oral health care.

Formatted: Font: Italic

Keywords: *Streptococcus mutans*, Toluidine Blue O, nanoliposomes, antibacterial efficacy, biofilm, dental caries

Introduction

Dental caries, the most common infectious oral cavity disease, is a biofilm-mediated progressive disease that results in tooth cavitation.(1) *Streptococcus mutans* is the major cariogenic species in the dental biofilm. Tooth surface adhesion, biofilm formation, acidogenicity through glycolysis, aciduricity, and glucosyltransferase-mediated extracellular polysaccharide synthesis are the cariogenic attributes of *Streptococcus mutans*~~*S. mutans*~~.(2) Biofilms are complex, structurally organized microbial colonies and extracellular polymeric substances (EPS), a biochemical mixture of polysaccharides, proteins, glycopeptides nucleic acids and lipids. (3) EPS restricts the entry of antimicrobials, and host immune response components, thereby offering protection to microorganisms. The primary challenge for the conventional ~~A~~antibacterial agents such as chlorhexidine and triclosan ~~have effectively reduced caries biofilm.~~ is penetrating the biofilm. ~~matrix is a primary challenge for antibacterial agents~~. ~~The extracellular polymeric substances within the matrix~~matrixes in the biofilm absorbs the antibacterial agents, limiting their diffusion and availability within the biofilm interior. (4,5) Furthermore, the small pores ~~of extracellular polymeric substances~~EPS impede the passage of larger molecules, (6) necessitating significantly higher antimicrobial doses. Enzymatic inactivation of penetrated antibacterial agents within the biofilm further complicates its effectiveness.(7) Further, the anti~~microbial~~bacterial effectiveness is challenged by the dormant growth phase of bacteria inside the biofilm.(8) Once inside the biofilm, antibacterial~~microbial~~ agents primarily exert their effects by damaging cell walls or interfering with metabolic processes intracellularly.(9) This triggers the emergence of antimicrobial resistance through various mechanisms, including mutation, acquisition of exogenous resistance genes, horizontal transfer of resistance genes, and efflux pump activity.(8,10) Additionally, the mosaic structure

of biofilms generates gradients of oxygen, nutrients, pH, and redox potential, leading to heterogeneity in growth rate and metabolic activity across different biofilm layers, which demand high specificity of ~~antimicrobials~~antibacterials. (11) Besides microbial resistance, allergic reactions to c~~C~~hlorhexidine (12), ability to form calculus by being incorporated into the dental plaque (13), and increased microleakage (13). ~~Further,~~These challenges ~~it~~ encourage~~s~~ research on exploring alternatives to the~~se~~ existing antimicrobials. Antimicrobial photodynamic therapy (aPDT) has appeared as a non-invasive alternative to modifying cariogenic biofilm.(14,15) where light of appropriate wavelength activates photosensitizers, thus releasing singlet oxygen. This singlet oxygen eventually causes tissue deterioration and cell death through necrosis or apoptosis.

The antibacterial effect of Toluidine Blue O (TBO) as a photosensitizer has been explored in a few studies, (16–20) particularly against gram-positive bacteria like *Streptococcus mutans* ~~*S. mutans*~~ due to their cationic and hydrophilic nature. However, there is wide variation in the effective antibacterial concentration of TBO against *S~~s~~treptococcus mutans*, which owes to inconsistency in the purity of TBO, delivery system of TBO and light parameters. Adequate penetration of the TBO into the biofilm is a noteworthy challenge for antibacterial efficacy, which in turn is dependent upon the purity and delivery system of TBO. Moreover, poor stability and limited bioavailability further limit the photosensitising properties of TBO. To optimize the photosensitising properties of TBO in harnessing the bacterial reduction outcome, we have attempted to minimise the above limitations of TBO-mediated aPDT by synthesising a purer form of TBO (ITBO). Nanoliposomes have been investigated for their biofilm penetrating potential, cell membrane-fusogenicity and biodegradability (10) which simultaneously improves the biocompatibility of the drug-loaded in it. Therefore, further, we attempted to load the ITBO in the core of the nanoliposome (NLITBO). The objective of the present research was to test the antibacterial efficacy of NLITBO against planktonic suspension of clinical strains of *Streptococcus mutans*~~*S. mutans*~~. The antibacterial activity of NLITBO, when supported by such experiments, could be applied to caries and various other diseases where *Streptococcus mutans* is a primary component of the biofilm. The pharmaceutical industry could harness this technology to develop novel antimicrobial therapies against resistant biofilms, eventually improving patient outcomes and reducing healthcare costs associated with chronic infections.

Methodology:

1. Materials and instrumentations

In addition to the general laboratory equipment, we have used rotary vacuum evaporator, bath sonicator, laboratory lyophilizer, UV/Vis spectrophotometry, zeta sizer, Scanning electron microscope (SEM), and Transmission electron microscope (TEM) for characterization of NLITBO, red LED light source, 96 well plate, and stereomicroscope for in-vivo antibacterial efficacy testing. The list of chemicals, reagents and media are listed in table number 1.

~~m~~Material	Company	CAS/Catalog number
Improved Toluidine Blue O (ITBO)	Synthesised as mentioned in Indian patent number 543908	NA
Soya-L-α-lecithin (SL)	**Sigma-Aldrich**	**8002-43-5**
Cholesterol (CHL)	**Sigma-Aldrich**	**57-88-5**
Chloroform	**Sigma-Aldrich**	67-66-3
Butylated Hydroxyl Toluene (2% w/v)	**Sigma-Aldrich**	128-37-0
Ethanol	**Sigma-Aldrich**	64-17-5
Muller Hinton Agar	Himedia	M173
Muller Hinton Broth	Himedia	M391
Nutrient Broth	Himedia	M002
Dimethyl Sulfoxide	Emplura	SA1S70054
Chlorohexidine gluconate	Zodenta	CG-148
Blood Agar Base	Himedia	M073
10X PBS	Himedia	ML023

Table 1 – List of chemicals, reagents, and media used in the study

~~1~~2. Synthesising Improved Toluidine blue O (ITBO)

ITBO was synthesised by the method as suggested in the Indian patent ~~application~~ ~~(202131053258~~ (number 543908) ~~A~~ dtd ~~2~~01~~6~~.0~~5~~7.2024~~3~~). (21)

3~~1~~. Preparation of NLITBO

For the preparation of drug-loaded nanoliposomes, plant-based phospholipids like soya-L-α-lecithin w~~ere~~as used as the main lipid component. (22,23) ~~(ref)~~ Along with that, cholesterol was also used to stabilize the lipid structure. (24) The experimental nanoliposomes were developed using the thin film hydration method with the required modifications.(25) ~~(ref)~~ Briefly, the amount of soya-L-α-lecithin (SL) and cholesterol (CHL) were dissolved in the required chloroform in a 250 ml round bottom flask. To this mixture, butylated hydroxyl toluene (2% w/v) was added as an antioxidant to prevent oxidation of lipids. The prepared mixture was then subjected to gentle rotation and evaporation of the solvent in a rotary vacuum evaporator (Rotavap, PBU-6, Superfit, Mumbai, India), connected with a water bath at 40 °C. Toluidine blue was added, and a thin film was formed along the inner wall of the round bottom flask. The flask was then kept in a desiccator overnight to remove organic solvent residues still in the thin film. On day 2, the formed thin film was hydrated with phosphate buffer saline (PBS), pH 7.4, for 1 h at 130-150 rpm rotation. Following hydration, the mixture was subjected to sonication in a bath-type sonicator to reduce the size of liposomes to the nanoscale range. Following sonication, the formulation was kept in a refrigerator overnight at 4 °C. On day 3, the sample was subjected to cold centrifugation at 15,000 rpm for 45 minutes. The supernatant was discarded, and the sediments were collected and stored at –20 °C overnight. The pre-cooled samples were dried in a laboratory lyophilizer for 8-10 hours to obtain the dry powdered product.

~~2~~4. Physico-chemical characterization of NLITBO

a) *Percentage Yield, Percentage drug loading and loading efficiency*: Lyophilized NLITBOs were weighed after each production batch, and the percentage yield was measured as % Yield= (Amount of NLITBOs obtained after lyophilization / Total amount of components used in the formulation batch) × 100. Briefly, the amount of lyophilized NLITBOs was dissolved in ethanol and water in a 7:3 ratio. The sample was then vortexed for 2 min, sonicated for 5 min and centrifuged at 12,000 rpm. The supernatant was examined at 631.0 nm using a UV/VIS spectrophotometer (Beckman, Fullerton, CA, USA). The following formula was calculated for the percentage drug loading and loading efficiency in NLITBOs.

Formatted: Highlight

Drug loading (%) = (Amount of TBO in NLITBOs / Amount of NLITBOs obtained) × 100

Drug loading efficiency (%) = (Practical TBO loading/ Theoretical TBO loading) × 100

b) *Determination of average vesicle size, polydispersity index (PDI) and Zeta potential:* To determine the average hydrodynamic size, the PDI and zeta potential of NLTBOs were evaluated using the Zeta sizer (Nano ZS 90, Malvern Instruments Ltd., UK) (*Satapathy et al., 2023*). Briefly, lyophilized NLITBOs were diluted with deionized water, followed by sonication for 5 minutes. At 25 ± 0.5 ºC, the sample was observed using a Zeta sizer.

Formatted: Not Highlight

c) Determination of surface features

The surface morphology and interior structure of the NLITBO nanovesicles were investigated using SEM and TEM, respectively. The lyophilized samples were spread out over a stub on a carbon tape. The sample was then vacuum dried, followed by gold coating over the surface, and was observed under SEM (JSM 6100; JEOL, Tokyo, Japan). NLITBO was suspended in deionized water and put onto the carbon-coated copper grid (mesh), which was then air-dried. The dried sample was then analysed under TEM (Tecnai G2 Spirit BioTwin FP 5018/40, FEI, Hillsboro, USA).

d) ITBO release assessment in vitro

10 mg of lyophilized NLITBOs was suspended in phosphate-buffered saline (PBS, pH of 7.4) and was placed in a dialysis bag (Himedia dialysis membrane-60, Mumbai, India). Two open ends of the dialysis bag were tied together using cotton thread, and the assembly was placed in a beaker filled with 50 millilitres of PBS. The beaker was placed over a magnetic stirrer (rotation speed: 300 rpm), and at fixed intervals, 1 millilitre of the release medium was removed with simultaneous replenishment of fresh PBS. The samples were analysed using a UV-visible spectrophotometer at a fixed wavelength of 631.0 nm. The amount of ITBO released from NLITBOs was determined by the extrapolation method from the standard calibration curve.

e) Bacterial strain and culture condition

Clinical strains of *Streptococcus mutans*, isolated from patients with caries as described previously,(26) were cultured in blood agar (Hi-media) and maintained in the laboratory. The isolation and identification of the bacteria followed the standard microbiological procedure, ensuring the authenticity and clinical relevance of the strains. The strains were cultured in Muller Hinton agar media for experimentation. Bacterial Inoculum was prepared by

inoculating *Streptococcus mutans* ~~*S. mutans*~~ culture in Muller Hinton broth and incubated until the culture reached the logarithmic growth phase.

f) Zone of inhibition(ZOI)

In vitro, the antibacterial activity of NLITBO was determined by the agar well diffusion method. 0.5 McFarland standard culture (1.5×108 cfu mL−1) of *Streptococcus mutans* ~~*S. mutans*~~ was prepared in Muller-Hinton agar media (HI media, India). 100 µL inoculum of *Streptococcus mutans* ~~*S mutans*~~ was spread onto Muller Hinton agar plates using a sterile cotton swab and allowed to dry, and wells were made with a sterile borer in the inoculated agar plates. 1 microgram of lyophilised NLITBO was dissolved in 1 ml of distilled water. 100 µL volumes of each test concentration were propelled into the culture plate of *Streptococcus mutans*~~*S. mutans*~~. Then, the plate was incubated for 24 h at 37 °C. 10% aqueous Dimethyl sulfoxide (DMSO) was used as the negative control; 2% c~~C~~hlorhexidine was used as the positive control. The antimicrobial activity of NLITBO was observed by recording the ~~zone of inhibition~~ZOI. The experiments were performed in triplicates, and the mean diameter of the ~~zone of inhibition (~~ZOI~~)~~ with standard deviation was recorded.

g) Minimum inhibitory concentration (MIC) *determination*

The ~~Minimum inhibitory concentration (MIC)~~MIC of NLITBO was determined by the colourimetric method in 96 well microtiter plates (Tarsons).(27) A stock solution of NLITBO in distilled water was made. The designing of the 96-well microplate was performed as follows: One column each was designed for positive and negative control. Seven test columns were composed of serial concentrations of NLITBO (0.9, 0.8, 0.7, 0.6, 0.5, 0.4, 0.3 µg/ ml respectively). The microbial culture was inoculated into Nutrient broth. The inoculum density was adjusted to approximately 0.5 McFarland standard (1-2 x 10^8 CFU/mL).100 µl of NLITBO solutions having concentrations ranging from 0.9 µg to 0.3 µg along with 100 µl of the prepared bacterial culture were added in each column of 96 well microtiter plate ensuring the final volume in each well is 200 µL. The plate was covered to prevent contamination and light and incubated at 37°C for 10 minutes. After incubation, each well was exposed to red LED light with a wavelength of 650nm and energy density approximating 9.1 J/cm^2. At the same energy density, the experiment was repeated thrice with three different combinations of power density and time (0.1 W/cm^2 + 90s, 0.08 W/cm^2 + 110s, 0.06 W/cm^2 + 150s). The light

was applied to each well using a round tip with an 8 mm diameter attached to the light-emitting device (Novo Duo, Novolase, India). 20 µL of TTC solution (0.2% w/v) was added to each well, including the positive and negative controls. The positive control was without NLITBO, and the negative control was without the bacteria. Wells other than the receiving wells were blocked from light by aluminium foil. The tip of the light device accurately fits the surface of each well, which is approximately 7 mm in diameter, so a uniform sphere of light is applied in the suspension mix. The plate was incubated overnight at room temperature to allow the reduction of TTC by metabolically active microorganisms. The wells were compared to determine the lowest concentration of TBO, which shows no or minimal colour change or formazan formation. The colour change was read visually, and a stereomicroscope (Motic, USA) was used to observe red formazan precipitates in the wells. The experiment was conducted in triplicate, and the mean concentration of TBO at which bacterial growth was inhibited was accepted as the MIC.

h) Statistical tests: Mean of ZOI among four groups were compared by Analysis of Variance (ANOVA) to observe the differences. To assess the variability across repeated experiments we calculated mean and standard deviation of MIC of TBO.

Formatted: Font: Not Bold

Results

a) Percentage~~%~~ Yield, Percentage ~~%~~ drug loading, and ~~%~~ Percentage loading efficiency of experimental NLITBOs

The three best formulations were selected from several formulation batches based on their drug-carrying capacity. Further, out of the three reported formulations, NLITBO-2 was found to be suitable (Table 1). The % drug loading for NLITBO-2 was 9.3±0.4 %, whereas 3.6±1.1% and 5.2±1.02 % for NLITBO-1 and NLITBO-3, respectively. NLITBO-2 also showed higher loading efficiency (84.4±7.6%) with yield percentage (73.5%) than the other two formulations (Table 2~~1~~). Based on these parameters, we selected NLITBO-2 for further studies.

Table 2~~1~~. Composition and percentage yield, loading and efficiency of three formulations

Formulation code	~~CHL:~~ SL:CHL	ITBO Quantity (mg)	% yield	Practical % loading	% Loading efficiency
NLTBO -1	1:1	15	61.4	3.6±1.1	58.3 ±5.1
NLTBO -2	1:3	15	73.5	9.3±0.4	84.4±7.6
NLTBO -3	1:5	15	56.6	5.2±1.02	61.3 ±0.4

b) Size analysis and zeta potential of selected NLTBOs

DLS data revealed that the selected formulation (NLTBO-2) was well within the desired nano-size range (Z-avg = 123.52 nm) (Fig.1A). The PDI value was found to be 0.57. Zeta potential of NLTBO-2 was reported to be -39.54mV (Fig.1 B).

c) Surface and internal structure analysis

SEM and TEM results depicted the successful formulation of NLITBO within the desired nano-range. All formed nanovesicles were spherical with smooth surfaces (Figure 2A). Most NLTBO-2 had a homogeneous size distribution, and there was a strong correlation between the vesicle sizes and the average size found in the DLS data. TEM study depicted a clear formation of uni-lamellar vesicular structures (Figure 2B). Nanoliposome~~al~~ vesicles with a spherical form, undistorted surface, and darker interior were observed in the TEM image (Figure 2B). The vesicles had no leaks or perforations, and the lamellarity was unbroken. The size of the vesicle was determined to be within 100 nm, which too supported by SEM and DLS data.

d) In vitro drug release study

The in vitro drug release study demonstrated sustained release characteristics of the NLITBO-2 at pH 7.4. At 24 h, a cumulative 79.81 % of ITBO was released from NLITBO-2 (Figure 3).

e) *Zone of inhibition(ZOI)*

Mean ZOI of the ~~P~~positive control, 2% chlorhexidine gluconate, 1 µg/ml NLITBO, 0.5 µg/ml NLITBO, and 0.25 µg/ml NLITBO were 1.2±0.3mm, 0.7±0.2mm, 0.3±0.1mm, and 0 mm respectively. ANOVA test demonstrated no significant difference between the four groups (p>0.05). ~~showed a wider zone of inhibition (1.2±0.3mm), whereas the zone of inhibition~~

~~produced by NLITBO was maximum (0.7±0.2mm) at 1 microgram/ml, intermediate (0.3±0.1mm) at 0.25 µg /ml and none at 0.25microgram/ml of NLITBO.~~

f) MIC of NLITBO

We observed no colour change in the test well containing 0.6 µg /ml ±~~(SD=~~0.03) NLITBO, indicating clear inhibition of bacterial growth to a gradual increase in the colour intensity as the concentration of NLITBO decreases. Therefore, we concluded that the MIC of NLITBO is 0.60 µg /ml ±~~(SD=~~0.03). Both positive and negative controls did not show any change.

Discussion

This experiment has explored the antibacterial efficacy of NLITBO against planktonic suspension of clinical strains of *Streptococcus mutans* ~~S mutans~~, a significant cariogenic species. This study assessed the potential of NLITBO as a photosensitiser in aPDT, focusing on the advantage of nanoliposomes as a delivery system and the purer form of TBO (ITBO) as a photosensitiser. The results showed that NLITBO exhibited significant antibacterial activity against *Streptococcus mutans*~~*S. mutans*~~. The ~~zone of inhibition~~ZOI produced by 1 µg/ml NLITBO (0.7+0.2~~—~~ mm) alone without PDT is not significantly~~slightly~~ different~~less~~ than ZOI produced by 2% chlorhexidine (1.2+0.3~~——~~ mm) (p value>0.05) but promising enough to warrant further exploration of MIC of aPDT using NLITBO. This indicates that the antimicrobial effect of NLITBO is equivalent to chlorhexidine even without the light activation. This may be attributed to improved stability, improved cellular uptake, membrane disruption, and the synergistic effects of the nanoliposome carrier in NLITBO. Further experiment on antimicrobial effect of NLITBO activated by light can be explored.

The successful synthesis of ITBO and incorporation into the nanoliposome was well characterized by the appropriate composition ratio of soya α lecithin and cholesterol (1:3), high percentage yield (73.5%), drug loading capacity (84.4±7.6), and surface and interior characteristics ~~()~~. Incorporating soya~~-~~α~~-~~lecithin and cholesterol in 5 :~~a~~ 1 ratio has been found to extend the stability of cinnamon oil for 96 hrs and enhance the anti-biofilm action against methicillin-resistant staphylococcus aureus (MRSA) by almost 10 times as compared to free cinnamon oil. (28) The lower value of PDI (0.57) indicates a homogenous and narrow distribution pattern of the formulation.(29)~~ The 39.5mV~~ Z~~z~~eta potential of -39.5mV classifies NLITBO ~~classifies it~~ as an anionic nanoliposome, which ~~helps maintain~~improves the stability in suspension. Higher similar charges between the nanovesicles create strong repulsive forces, keeping vesicles separated for longer. Stable nano formulations have been reported to have a

Formatted: Not Highlight

Formatted: Not Highlight

surface charge beyond -30 mV to +30 mV.(30,31) The drug release profile~~s~~ exhibited a controlled release pattern over the study period, which is beneficial for effective clinical application, reducing dose and dose-related side effects. (32) NLITBO showed antibacterial activity even without photoactivation, as observed by mean ZOI in the present study, which implied inherent antibacterial properties of NLITBO that can be~~are~~ further potentiated by red light application. This finding is~~has~~ negated by Shen et al (33) but supported by Nastri et al (34) who demonstrated the antibacterial effect of TBO at 10 µM concentration which was further potentiated by light application. The antibacterial activity of NLITBO without light may be attributed to the improved TBO and coating with anionic nanoliposome. NLITBO, owing to its zeta potential -39.5mV, is an anionic nanoliposome carrying cationic photosensitiser, ITBO. Anionic nanoliposomes may be less effective than cationic liposomes against gram-positive bacteria like *Streptococcus mutans*~~*S. mutans*~~.(35) However, upon activation of the cationic photosensitiser, (ref) ITBO may penetrate the dental biofilm and *Streptococcus mutans* ~~*S .mutans*~~ cell wall more effectively than cationic nanoliposome carrying cationic ITBO as it may not penetrate biofilm_ well due to the overall charge repulsion. Studies suggested that nanoliposomes loaded with cationic photosensitizers are designed for hydrophobic interaction with the negatively charged surface of gram-positive bacterial cells. This allows for the diffusion of nanoliposome-loaded photosensitisers into *Streptococcus mutans*~~S. mutans~~. (36) The liposome-bacteria fusion was shown to enhance the diffusion of the drugs into *Pseudomonas aeruginosa*. (37,38) This selective targeting enhances the specificity and efficacy of aPDT against gram-positive pathogens. Upon exposure to red light, the photosensitizer, ITBO loaded within the nanoliposomes, becomes excited. (39) It undergoes photochemical reactions, leading to the generation of reactive oxygen species (ROS), such as singlet oxygen and free radicals. (40) The generated ROS exert their antimicrobial effects by inducing oxidative damage to various cellular components of the gram-positive bacterial cells, eventually disrupting cellular functions and promoting cell death. This disruption facilitates the entry of ROS into the bacterial cytoplasm, enhancing their antimicrobial activity. Moreover, sustained release characteristics of the NLITBO-2, as found in this study, shall prove beneficial as there would be a consistent therapeutic effect even in the presence of saliva as the experiment was conducted at pH 7.4. A significant portion of ITBO (79.81 %) was released from NLITBO-2 by the end of 24 hours. This attribute can be advantageous in a targeted and sustained approach during aPDT against gram-positive bacteria, providing a promising strategy for combating bacterial infections while minimising the risk of antimicrobial resistance and enhancing patient compliance.

This study has identified MIC of NLITBO against clinical strains of S~~s~~*treptococcus mutans* suspension with red light (wavelength – 650 nm, power density - 0.1mW/cm^2 for 90 seconds). Effective antimicrobial activity of TBO in *Streptococcus mutans* ~~*S mutans*~~ suspension was observed in the concentration range of 0.01 to 3.08 mg/ml. (16–20,41–43) the effective concentration for anti-biofilm activity in *Streptococcus mutans* ~~*S. mutans*~~ was 0.1mg/ml (20,44–46) and 41.067 mg/ml. (47) (45,46,48,49) Although the effective antibacterial concentration should vary according to the nature of bacterial culture, there is a huge difference in the effective concentration of TBO in studies on bacterial suspensions, too. (16–20,41–43) This variation may be attributed to the difference in strains of s mutans, light parameters such as energy density, power density, incubation time and time of red-light exposure across studies. Energy densities reported in different studies ranged from 9J/cm2 to 180J/cm2 (19,26,33,35–38), while power densities ranged from 100 mW to 500 mW. Duration of light exposure also varied from 30 seconds (50) to 12 minutes 50 seconds (18). Except for one (39) rest of the studies have used the commercial strain. In general, clinical strains exhibit more genetic diversity, and the findings on clinical strains are applicable in clinical settings. Therefore, the present result can find a broader applicability. ~~In fact,~~ T~~t~~here are two clinical studies (27,40) which evaluated the antimicrobial potential of TBO ~~clinically~~ in children with early childhood caries but failed to significantly reduce the bacterial count of the saliva. This ~~inconsistent~~contradicting result could be due to the absence of an appropriate drug delivery system. Nanoliposomal formulation, an effective drug delivery system, is known to enhance the bioavailability and biodistribution of the loaded drug.(51) Afrasiabi et al. (52) have shown nanoliposomes' enhanced antimicrobial photodynamic therapy containing curcumin and doxycycline against *Aggregatibacter actinomycetemcomitans*. Therefore, we, for the first time, have emphasised the nanoliposome encapsulation of ITBO and demonstrated that NLITBO is an effective antibacterial agent against clinical strains of *Streptococcus mutans* under optimal red-light parameters (wavelength 650nm, power density 0.1 W, energy density 9-9.1 J, 90seconds time) with a MIC of 0.6µg/ml. These conditions are effective, and the biocompatible concentration of NLITBO and light energy dosage are selected to target the 24-hour incubated bacterial suspension of *Streptococcus mutans*~~*S. mutans*~~.

Firstly, our study demonstrated the minimum inhibitory concentration (MIC) of NLITBO. The observed MIC of 0.60 µg/mL for NLITBO indicates its potent bactericidal activity, highlighting its potential as a promising candidate for caries prevention. Supporting this previous study (19) has also shown better antibiofilm action of TBO-silver nanoparticle

conjugates on *Streptococcus mutans* ~~*S. mutans*~~ than TBO alone. TBO has also been added with microbial efflux pump inhibitors for successful biofilm inactivation. (53) The enhanced efficacy of NLITBO can be attributed to several factors. One plausible explanation is the improved bioavailability and targeted delivery of TBO facilitated by nanoliposomes. Nanoliposomes offer a stable and controlled release platform, allowing for prolonged exposure of TBO to bacterial cells, thus maximizing its antimicrobial effects and minimizing potential adverse effects on surrounding tissues. While the conventional antimicrobials often struggle to penetrate the extracellular polymeric substance barrier in the biofilm to effectively target bacteria, nanoparticles' (NP) ability to penetrate the biofilm can inhibit the biofilm formation. (54) NPs can exert the antibacterial activity through different mechanisms such as cell wall disruption, interaction with DNA/RNA or proteins, biofilm inhibition, or ROS generation. (55) There are three stages during the interaction of NP with biofilm. NP transported to the surface of the biofilm, gets attached to the biofilm followed by migration in the biofilm. Several factors such as physicochemical properties of NPs and extracellular matrix influence how the individual phases take place. (56) Additionally, the nanoliposomes get attached to the cell wall of bacteria and enhance ITBO penetration into the bacterial cytoplasm, where it gets photoactivated and the nascent oxygen released causes bacterial death. Furthermore, the nanoliposome carrier system may protect ITBO from degradation and enhance its retention within the oral cavity, prolonging its therapeutic effects.

Despite the promising findings of our study, several limitations should be considered. Firstly, our study was conducted in vitro, and further investigations are needed to assess the efficacy of NLITBO in vivo. Additionally, while our study focused on *Streptococcus mutans* ~~*S. mutans*~~, future research should explore its effects on other oral pathogens and its potential interactions with the oral microbiome. Furthermore, the safety profile

of NLITBO, including potential cytotoxicity and adverse effects, warrants thorough evaluation before clinical translation. The sensitivity of planktonic bacteria to antimicrobial agents is far lower than biofilm-grown bacteria.(57) Therefore, further research may be directed to find out the biofilm inhibitory concentration and biofilm microbicidal concentration, which would effectively explore the efficacy, safety, and practical application of ITBO in the context of dental care to determine its role in preventing and managing infections associated with *Streptococcus mutans* ~~*S. mutans*~~.

In conclusion, our study demonstrates the enhanced antimicrobial efficacy of NLITBO and the MIC with optimum light parameters during aPDT against *Streptococcus mutans*. This research's purity and drug delivery system may have clinical benefits when exploring caries preventive agents. Further research is needed to validate our findings in vivo, assess safety profiles, and explore potential applications in clinical practice. Overall, NLITBO represents an innovative and promising approach to caries prevention, addressing critical challenges in antimicrobial therapy and paving the way for improved oral health outcomes.

Acknowledgement: Nil

Funding : The authors did not receive any funding for the conduct of this research. The APC is funded by Ajman University

Conflict of Interest: None to declare

Figure Legends

Figure 1: DLS demonstrating Size (A) and Zeta potential (B) of NLITBO

Figure 2: Surface structure in SEM (A) and interior structure in TEM (B) of NLITBO

Figure 3: In vitro release behaviour of NLITBO in PBS, pH 7.4

Figure 4: Zone of Inhibition 1. NLITBO (0.25 µg /µl) - no zone; 2. NLITBO (0.5 µg /ml) - 0.3±0.1 mm.; 3. NLITBO (1 µg /µl) - 0.7±0.2 mm ; 4. Negative Control - no zone; 5; Chlorhexidine gluconate 2% - 1.2 ± 0.3 mm

Table legends

Table 1 – List of chemicals, reagents and media used in the study.

Table 2~~1~~ – Composition and percentage yield, loading and efficiency of three formulations

References

1. Marcenes W, Kassebaum NJ, Bernabé E, Flaxman A, Naghavi M, Lopez A, et al. Global burden of oral conditions in 1990-2010: a systematic analysis. J Dent Res. 2013 Jul;92(7):592–7.

2. Lynch DJ, Michalek SM, Zhu M, Drake D, Qian F, Banas JA. Cariogenicity of Streptococcus mutans Glucan-Binding Protein Deletion Mutants. Oral Health Dent Manag [Internet]. 2013 Dec [cited 2024 Apr 22];12(4):191–9. Available from: https://www.ncbi.nlm.nih.gov/pmc/articles/PMC4437697/

3. Chávez de Paz LE, Resin A, Howard KA, Sutherland DS, Wejse PL. Antimicrobial effect of chitosan nanoparticles on streptococcus mutans biofilms. Appl Environ Microbiol. 2011 Jun;77(11):3892–5.

4. Gupta TT, Karki SB, Fournier R, Ayan H. Mathematical Modelling of the Effects of Plasma Treatment on the Diffusivity of Biofilm. Appl Sci [Internet]. 2018 Oct [cited 2024 Apr 17];8(10):1729. Available from: https://www.mdpi.com/2076-3417/8/10/1729

5. Oliveira M, Brugnera D, Piccoli R. Biofilms in the dairy industries: general aspects and the use of essential oils as a new control alternative. Rev Inst Laticínios Cândido Tostes. 2013 Feb 1;68:65–73.

6. Gilbert P, Allison DG, McBain AJ. Biofilms in vitro and in vivo: do singular mechanisms imply cross-resistance? J Appl Microbiol. 2002;92 Suppl:98S-110S.

7. Albayaty YN, Thomas N, Hasan S, Prestidge CA. Penetration of topically used antimicrobials through *Staphylococcus aureus* biofilms: A comparative study using different models. J Drug Deliv Sci Technol [Internet]. 2018 Dec 1 [cited 2024 Apr 17];48:429–36. Available from: https://www.sciencedirect.com/science/article/pii/S1773224718308256

8. Smith AW. Biofilms and antibiotic therapy: is there a role for combating bacterial resistance by the use of novel drug delivery systems? Adv Drug Deliv Rev. 2005 Jul 29;57(10):1539–50.

9. Neville N, Jia Z. Approaches to the Structure-Based Design of Antivirulence Drugs: Therapeutics for the Post-Antibiotic Era. Mol Basel Switz. 2019 Jan 22;24(3):378.

10. Dos Santos Ramos MA, Da Silva PB, Spósito L, De Toledo LG, Bonifácio BV, Rodero CF, et al. Nanotechnology-based drug delivery systems for control of microbial biofilms: a review. Int J Nanomedicine. 2018;13:1179–213.

11. Stewart PS, Franklin MJ. Physiological heterogeneity in biofilms. Nat Rev Microbiol. 2008 Mar;6(3):199–210.

12. Pemberton MN, Gibson J. Chlorhexidine and hypersensitivity reactions in dentistry. Br Dent J. 2012 Dec;213(11):547–50.

13. Zanatta FB, Antoniazzi RP, Rösing CK. Staining and calculus formation after 0.12% chlorhexidine rinses in plaque-free and plaque covered surfaces: a randomized trial. J Appl Oral Sci Rev FOB. 2010;18(5):515–21.

14. Soukos NS, Goodson JM. Photodynamic therapy in the control of oral biofilms. Periodontol 2000 [Internet]. 2011 [cited 2024 Apr 19];55(1):143–66. Available from: https://onlinelibrary.wiley.com/doi/abs/10.1111/j.1600-0757.2010.00346.x

15. Gursoy H, Ozcakir-Tomruk C, Tanalp J, Yilmaz S. Photodynamic therapy in dentistry: a literature review. Clin Oral Investig. 2013 May;17(4):1113–25.

16. Aragão MGB, Costa CAG de A, Lima RA, Rodrigues LKA, Duarte S, Zanin ICJ. Comparative Effect of Two Red Lights on Streptococcus mutans Biofilms and Assessment of Temperature Variances in Human Teeth During In Vitro Photodynamic Antimicrobial Chemotherapy. Photobiomodulation Photomed Laser Surg. 2019 Jan;37(1):31–7.

17. Araújo PV, Teixeira KIR, Lanza LD, Cortes ME, Poletto LTA. In vitro lethal photosensitization of S. mutans using methylene blue and toluidine blue O as photosensitizers. Acta Odontol Latinoam AOL. 2009;22(2):93–7.

18. Misba L, Zaidi S, Khan AU. Efficacy of photodynamic therapy against Streptococcus mutans biofilm: Role of singlet oxygen. J Photochem Photobiol B. 2018 Jun;183:16–21.

19. Misba L, Kulshrestha S, Khan AU. Antibiofilm action of a toluidine blue O-silver nanoparticle conjugate on Streptococcus mutans: a mechanism of type I photodynamic therapy. Biofouling. 2016;32(3):313–28.

20. Zanin ICJ, Lobo MM, Rodrigues LKA, Pimenta LAF, Höfling JF, Gonçalves RB. Photosensitization of in vitro biofilms by toluidine blue O combined with a light-emitting diode. Eur J Oral Sci. 2006 Feb;114(1):64–9.

21. Panda S. SYNTHESIS OF TOLUIDINE BLUE O AND A KIT FOR MUCOSAL APPLICATION. 543908, 2024.

22. Le NTT, Cao VD, Nguyen TNQ, Le TTH, Tran TT, Hoang Thi TT. Soy Lecithin-Derived Liposomal Delivery Systems: Surface Modification and Current Applications. Int J Mol Sci. 2019 Sep 23;20(19):4706.

23. van Hoogevest P, Wendel A. The use of natural and synthetic phospholipids as pharmaceutical excipients. Eur J Lipid Sci Technol EJLST. 2014 Sep;116(9):1088–107.

24. Briuglia ML, Rotella C, McFarlane A, Lamprou DA. Influence of cholesterol on liposome stability and on in vitro drug release. Drug Deliv Transl Res. 2015 Jun;5(3):231–42.

25. TAMADDON L, MOHAMADI N, BAVARSAD N. Preparation and Characterization of Mucoadhesive Loratadine Nanoliposomes for Intranasal Administration. Turk J Pharm Sci. 2021 Aug;18(4):492–7.

26. Mohanty M, Govind S, Rath S. Prevalence of salivary microbial load and lactic acid presence in diabetic and non-diabetic individuals with different dental caries stages. Restor Dent Endod. 2024 Feb;49(1):e4.

27. Barnes V L, Heithoff DM, Mahan SP, House JK, Mahan MJ. Antimicrobial susceptibility testing to evaluate minimum inhibitory concentration values of clinically relevant antibiotics. STAR Protoc [Internet]. 2023 Aug 10 [cited 2024 May 14];4(3):102512. Available from: https://www.ncbi.nlm.nih.gov/pmc/articles/PMC10448204/

28. Cui H, Li W, Li C, Vittayapadung S, Lin L. Liposome containing cinnamon oil with antibacterial activity against methicillin-resistant Staphylococcus aureus biofilm. Biofouling. 2016;32(2):215–25.

29. Salah S, Awad GEA, Makhlouf AIA. Improved vaginal retention and enhanced antifungal activity of miconazole microsponges gel: Formulation development and in vivo therapeutic efficacy in rats. Eur J Pharm Sci Off J Eur Fed Pharm Sci. 2018 Mar 1;114:255–66.

30. Satapathy BS, Biswal B, Pattnaik S, Parida R, Sahoo RN. A mucoadhesive nanolipo gel containing Aegle marmelos gum to enhance transdermal effectiveness of linezolid for vaginal infection: In vitro evaluation, in vitro-ex vivo correlation, pharmacokinetic studies. Int J Pharm. 2023 Dec 15;648:123542.

31. Satapathy BS, Kumar LA, Pattnaik G, Barik B. Lomustine Incorporated Lipid Nanostructures Demonstrated Preferential Anticancer Properties in C6 Glioma Cell Lines with Enhanced Pharmacokinetic Profile in Mice. Acta Chim Slov. 2021 Dec 15;68(4):970–82.

32. Maiti S, Sen KK, Maiti S, Sen KK. Introductory Chapter: Drug Delivery Concepts. In: Advanced Technology for Delivering Therapeutics [Internet]. IntechOpen; 2017 [cited 2024 Aug 17]. Available from: https://www.intechopen.com/chapters/54825

33. Shen J, Liang Q, Su G, Zhang Y, Wang Z, Baudouin C, et al. In Vitro Effect of Toluidine Blue Antimicrobial Photodynamic Chemotherapy on Staphylococcus epidermidis and Staphylococcus aureus Isolated from Ocular Surface Infection. Transl Vis Sci Technol [Internet]. 2019 Jun 21 [cited 2024 May 14];8(3):45. Available from: https://www.ncbi.nlm.nih.gov/pmc/articles/PMC6590089/

34. Nastri L, Donnarumma G, Porzio C, De Gregorio V, Tufano MA, Caruso F, et al. Effects of Toluidine Blue-Mediated Photodynamic Therapy on Periopathogens and Periodontal Biofilm: In Vitro Evaluation. Int J Immunopathol Pharmacol [Internet]. 2010 Oct 1 [cited 2024 May 14];23(4):1125–32. Available from: https://doi.org/10.1177/039463201002300416

35. Drulis-Kawa Z, Dorotkiewicz-Jach A, Gubernator J, Gula G, Bocer T, Doroszkiewicz W. The interaction between Pseudomonas aeruginosa cells and cationic PC:Chol:DOTAP liposomal vesicles versus outer-membrane structure and envelope properties of bacterial cell. Int J Pharm. 2009 Feb 9;367(1–2):211–9.

36. Beaulac C, Sachetelli S, Lagace J. In-vitro bactericidal efficacy of sub-MIC concentrations of liposome-encapsulated antibiotic against gram-negative and gram-positive bacteria. J Antimicrob Chemother. 1998 Jan;41(1):35–41.

37. Sachetelli S, Khalil H, Chen T, Beaulac C, Sénéchal S, Lagacé J. Demonstration of a fusion mechanism between a fluid bactericidal liposomal formulation and bacterial cells. Biochim Biophys Acta. 2000 Feb 15;1463(2):254–66.

38. Mugabe C, Halwani M, Azghani AO, Lafrenie RM, Omri A. Mechanism of Enhanced Activity of Liposome-Entrapped Aminoglycosides against Resistant Strains of Pseudomonas aeruginosa. Antimicrob Agents Chemother [Internet]. 2006 Jun [cited 2024 Apr 9];50(6):2016–22. Available from: https://www.ncbi.nlm.nih.gov/pmc/articles/PMC1479138/

39. Najm M, Pourhajibagher M, Badirzadeh A, Razmjou E, Alipour M, Khoshmirsafa M, et al. Photodynamic Therapy Using Toluidine Blue O (TBO) Dye as a Photosensitizer against Leishmania major. Iran J Public Health. 2021 Oct;50(10):2111–20.

40. Oleinick NL, Evans HH. The photobiology of photodynamic therapy: cellular targets and mechanisms. Radiat Res. 1998 Nov;150(5 Suppl):S146-156.

41. Vahabi S, Fekrazad R, Ayremlou S, Taheri S, Zangeneh N. The effect of antimicrobial photodynamic therapy with radachlorin and toluidine blue on streptococcus mutans: an in vitro study. J Dent Tehran Iran. 2011;8(2):48–54.

42. Fekrazad R, Seraj B, Chiniforush N, Rokouei M, Mousavi N, Ghadimi S. Effect of antimicrobial photodynamic therapy on the counts of salivary Streptococcus mutans in children with severe early childhood caries. Photodiagnosis Photodyn Ther. 2017 Jun;18:319–22.

43. Hakimiha N, Khoei F, Bahador A, Fekrazad R. The susceptibility of Streptococcus mutans to antibacterial photodynamic therapy: a comparison of two different photosensitizers and light sources. J Appl Oral Sci Rev FOB. 2014 Apr;22(2):80–4.

44. de Sousa Farias SS, Nemezio MA, Corona S a. M, Aires CP, Borsatto MC. Effects of low-level laser therapy combined with toluidine blue on polysaccharides and biofilm of Streptococcus mutans. Lasers Med Sci. 2016 Jul;31(5):1011–6.

45. Balhaddad AA, AlQranei MS, Ibrahim MS, Weir MD, Martinho FC, Xu HHK, et al. Light Energy Dose and Photosensitizer Concentration Are Determinants of Effective Photo-Killing against Caries-Related Biofilms. Int J Mol Sci. 2020 Oct 15;21(20):7612.

46. Teixeira AH, Pereira ES, Rodrigues LKA, Saxena D, Duarte S, Zanin ICJ. Effect of photodynamic antimicrobial chemotherapy on in vitro and in situ biofilms. Caries Res. 2012;46(6):549–54.

47. de Oliveira FS, Cruvinel T, Cusicanqui Méndez DA, Dionísio EJ, Rios D, Machado MAAM. The *in vitro* effect of Antimicrobial Photodynamic Therapy on dental microcosm biofilms from partially erupted permanent molars: A pilot study. Photodiagnosis Photodyn Ther [Internet]. 2018 Mar 1 [cited 2024 Apr 10];21:163–7. Available from: https://www.sciencedirect.com/science/article/pii/S1572100017304076

48. Shirato M, Nakamura K, Kanno T, Lingström P, Niwano Y, Örtengren U. Time-kill kinetic analysis of antimicrobial chemotherapy based on hydrogen peroxide photolysis against Streptococcus mutans biofilm. J Photochem Photobiol B. 2017 Aug;173:434–40.

49. Zanin ICJ, Gonçalves RB, Junior AB, Hope CK, Pratten J. Susceptibility of Streptococcus mutans biofilms to photodynamic therapy: an in vitro study. J Antimicrob Chemother. 2005 Aug;56(2):324–30.

50. Fekrazad R, Khoei F, Hakimiha N, Bahador A. Photoelimination of Streptococcus mutans with two methods of photodynamic and photothermal therapy. Photodiagnosis Photodyn Ther. 2013 Dec;10(4):626–31.

51. Fatfat Z, Karam M, Maatouk B, Fahs D, Gali-Muhtasib H. Chapter 7 - Nanoliposomes as safe and efficient drug delivery nanovesicles. In: Nayak AK, Hasnain MS, Laha B, Maiti S, editors. Advanced and Modern Approaches for Drug Delivery [Internet]. Academic Press; 2023 [cited 2024 Apr 8]. p. 159–97. Available from: https://www.sciencedirect.com/science/article/pii/B9780323916684000022

52. Afrasiabi S, Partoazar A, Chiniforush N. In vitro study of nanoliposomes containing curcumin and doxycycline for enhanced antimicrobial photodynamic therapy against Aggregatibacter actinomycetemcomitans. Sci Rep [Internet]. 2023 Jul 18 [cited 2024 Apr 8];13(1):11552. Available from: https://www.nature.com/articles/s41598-023-38812-4

53. Kishen A, Upadya M, Tegos GP, Hamblin MR. Efflux pump inhibitor potentiates antimicrobial photodynamic inactivation of Enterococcus faecalis biofilm. Photochem Photobiol. 2010;86(6):1343–9.

54. Mohanta YK, Chakrabartty I, Mishra AK, Chopra H, Mahanta S, Avula SK, et al. Nanotechnology in combating biofilm: A smart and promising therapeutic strategy. Front Microbiol. 2022;13:1028086.

55. Wang Y. Liposome as a delivery system for the treatment of biofilm-mediated infections. J Appl Microbiol. 2021;131(6):2626–39.

56. Shkodenko L, Kassirov I, Koshel E. Metal Oxide Nanoparticles Against Bacterial Biofilms: Perspectives and Limitations. Microorganisms. 2020 Oct;8(10):1545.

57. Lindhe J, Karring T, Lang NP. Clinical periodontology and implant dentistry. 4th ed. Oxford, UK: Blackwell; 2003.

Reviewer's Comments to the Author

Reviewer # 1

Topic is innovative. Manuscript is well written. Statistical test is mentioned. All references from reference list are depicted in the manuscript. Conclusion is in conformity with the objectives of the study.

Author response– Thank you for your encouraging words.

Reviewer #2:

Reviewer comment 1: The manuscript is quite a preliminary study. Without proper data, its not ready to be published.in Biofilms, there is always different bacterial species, so targeting alone, S. mutans is not the cure of the disease.

Author Response - While we acknowledge the complexity of biofilm communities and the presence of multiple bacterial species, this study was designed as a feasibility study to explore the efficacy of NLITBO against the primary contributor of the cariogenic biofilm which is *Streptococcus mutans*. Future research will involve expanding the scope to include other bacterial species commonly found in the biofilm, thereby providing a more comprehensive understanding of the potential of NLITBO in targeting polymicrobial biofilms. Our findings lay the groundwork for such studies by demonstrating the photosensitizing potential of NLITBO against a critical cariogenic species.

Reviewer comment 2 The treatment of planktonic suspension will not address the real problem. Biofilm existence comprising of S mutans and many others in case of dental caries.

Author Response – Yes, We agree with the reviewer and acknowledge that the treatment of planktonic suspensions will not address the real problem. However, our study aims to establish a foundational understanding of the antibacterial efficacy of NLITBO against *S. mutans* in planktonic form as a preliminary step. The promising result of this study may guide the next logical step to evaluate its effectiveness on dental biofilms.

Reviewer comment 3 Why both SEM and TEM. One was sufficient?

Author Response: We studied the surface morphology of NLITBO by SEM and internal structure of NLITBO by TEM so that both surface and internal properties are evaluated

Reviewer comment 4 Please prepare a biofilm of dental bacteria in vitro and then test the formulation. In planktonic mode against one bacteria. Of no use

Author response: Thank you for the insight. We agree that testing NLITBO in dental biofilm would provide a clinically relevant evaluation of its antibacterial efficacy. However, due to the time constraint and complexity of establishing a reliable in vitro dental biofilm model, it may not be feasible to conduct such experiment. Planktonic solutions are often used as a first line evaluation of

any novel antibacterial agent's efficacy and the result guides further research. We appreciate your concern and looking forward to explore this avenue in further studies.

Reviewer comment 5 :ATCC or accession number of S mutans

Author response: The S mutans strain was isolated clinically. The methods has been described in page 8 line 228-229

Reviewer comment 6 Statistical analysis?

Author response: Thank you for your valuable suggestions. We have elaborated more on the statistical analysis on page 10, line 278-280

Reviewer #3:

In this article, the authors prepared NLITBO, characterized its features, and evaluated the antibacterial activity against S.mutans. This would provide a new approach for preventing and treating dental caries.

Author response– Thank you for your encouraging words.

Reviewer comment 1

Introduction

-Line # 87-91: Add the latest reference; https://doi.org/10.1016/j.jtemb.2024.127448

Author response: We have added the reference as suggested.

Reviewer comment 2

-Line # 93-103: Add more detail about the biofilm and the effect of antibacterial agent on the biofilm. Compare with the other antibacterial agents and also add how the antimicrobial agent used in study are helpful in pharmaceutical industries.

Author response: Thank you for the insightful comments. We have added the further details of biofilm including effect of antibacterial agents on the biofilm. We also compared with other antibacterial agents and added the implication of this antimicrobial agent, i.e NLITBO in pharmaceutical industry.

Reviewer comment 3

Methodology

Add new separate heading with detail of reagents/chemicals, instrumentation, media etc. used in present study.

Author response: We have done the modification as suggested.

Reviewer comment 4

-Line # 136-138: Add the reference.

Author response: We have added the reference as suggested

Reviewer comment 5

-Line # 139: Reference

Author response: We have added the reference as suggested

Reviewer comment 6

-Add the references in each section of Methodology.

Author response: We have added the reference as suggested

Reviewer comment 7

Results

-Make sure bacterial species name must be written in correct form in all text.

Author response: We have done the modification as suggested.

Reviewer comment 8

Discussion

-Line #: 282, 283: This is the part of result section

Author response: We have done the modification as suggested.

Reviewer comment 9

-Line # 286-289: Why ZOI produced by NLITBO were higher than those of chlorhexidine, give strong reason.

Author response: Mean ZOI produced by NLITBO is not higher but not significantly different from mean ZOI produced by Chlorhexidine. As per your valuable suggestions I have substantiated with possible reasons in the discussion.

Reviewer comment 10

-Line # 292: Add reference.

Author response: Those empty parentheses is to write the values as found in this study. We have added the details.

Reviewer comment 11

-Line # 296, 297: Cite the proper reference.

Author response: We have added the reference as suggested

Reviewer comment 12

-Line # 302: Reference?

Author response: We have added the reference as suggested

Reviewer comment 13

-Line # 320: Reference?

Author response: We have added the reference as suggested

Reviewer comment 14

-S. mutans, correct it with same pattern in all text.

Author response: We have done the modification as suggested.

Reviewer comment 15

-Add the explanation, how the NPs affect on the biofilm producing bacterial structure?

Author response: We have added the explanation as suggested

Reviewer comment 16

-Lines # 345-348: Unnecessary statement. Is this related to this study?

Author response :Thank you for raising the concern. We believe the sentences are not explanatory enough for which we have edited these lines again. These sentences are a critical analysis of the studies which were conducted to extablish the effectiveness of TBO as a photosensitizer. Only one study has used the clinical strain similar to our study. In general, clinical strains exhibit more genetic diversity and the findings on clinical strains are applicable in clinical settings. Therefore, we have highlighted the broader applicability of our results. Similarly contradicting our establishment of antibacterial efficacy of NLITBO, two studies could not prove clinical effectiveness of TBO in early childhood caries, which may be attributed to the use of laboratory grade TBO, lack of appropriate delivery method and/or lack of optimum light parameters. In this light we have discussed the importance of nanoliposome as a drug delivery system.
