# Supplementary material for: Exploring the photosensitizing potential of Nanoliposome Loaded Improved Toluidine Blue O (NLITBO) Against Streptococcus mutans: An in-vitro feasibility study
Source: PLoS One. 2024 Oct 30;19(10):e0312521. doi: 10.1371/journal.pone.0312521 (PMC11524459; doi:10.1371/journal.pone.0312521)
Supplement: Attachment — Submitted filename: Response to reviewer document.docx [file pone.0312521.s002.docx]

Reviewer’s Comments to the Author

Reviewer # 1

Topic is innovative. Manuscript is well written. Statistical test is mentioned. All references from reference list are depicted in the manuscript. Conclusion is in conformity with the objectives of the study.

Author response– Thank you for your encouraging words.

Reviewer #2:

Reviewer comment 1: The manuscript is quite a preliminary study. Without proper data, its not ready to be published.in Biofilms, there is always different bacterial species, so targeting alone, S. mutans is not the cure of the disease.

Author Response - While we acknowledge the complexity of biofilm communities and the presence of multiple bacterial species, this study was designed as a feasibility study to explore the efficacy of NLITBO against the primary contributor of the cariogenic biofilm which is *Streptococcus mutans*. Future research will involve expanding the scope to include other bacterial species commonly found in the biofilm, thereby providing a more comprehensive understanding of the potential of NLITBO in targeting polymicrobial biofilms. Our findings lay the groundwork for such studies by demonstrating the photosensitizing potential of NLITBO against a critical cariogenic species.

Reviewer comment 2 The treatment of planktonic suspension will not address the real problem. Biofilm existence comprising of S mutans and many others in case of dental caries.

Author Response – Yes, We agree with the reviewer and acknowledge that the treatment of planktonic suspensions will not address the real problem. However, our study aims to establish a foundational understanding of the antibacterial efficacy of NLITBO against *S. mutans* in planktonic form as a preliminary step. The promising result of this study may guide the next logical step to evaluate its effectiveness on dental biofilms.

Reviewer comment 3 Why both SEM and TEM. One was sufficient?

Author Response: We studied the surface morphology of NLITBO by SEM and internal structure of NLITBO by TEM so that both surface and internal properties are evaluated

Reviewer comment 4 Please prepare a biofilm of dental bacteria in vitro and then test the formulation. In planktonic mode against one bacteria. Of no use

Author response: Thank you for the insight. We agree that testing NLITBO in dental biofilm would provide a clinically relevant evaluation of its antibacterial efficacy. However, due to the time constraint and complexity of establishing a reliable in vitro dental biofilm model, it may not be feasible to conduct such experiment. Planktonic solutions are often used as a first line evaluation of any novel antibacterial agent’s efficacy and the result guides further research. We appreciate your concern and looking forward to explore this avenue in further studies.

Reviewer comment 5 :ATCC or accession number of S mutans

Author response: The S mutans strain was isolated clinically. The methods has been described in page 8 line 228-229

Reviewer comment 6 Statistical analysis?

Author response: Thank you for your valuable suggestions. We have elaborated more on the statistical analysis on page 10, line 278-280

Reviewer #3:

In this article, the authors prepared NLITBO, characterized its features, and evaluated the antibacterial activity against S.mutans. This would provide a new approach for preventing and treating dental caries.

Author response– Thank you for your encouraging words.

Reviewer comment 1

Introduction

-Line # 87-91: Add the latest reference; <https://doi.org/10.1016/j.jtemb.2024.127448>

Author response: We have added the reference as suggested.

Reviewer comment 2

-Line # 93-103: Add more detail about the biofilm and the effect of antibacterial agent on the biofilm. Compare with the other antibacterial agents and also add how the antimicrobial agent used in study are helpful in pharmaceutical industries.

Author response: Thank you for the insightful comments. We have added the further details of biofilm including effect of antibacterial agents on the biofilm. We also compared with other antibacterial agents and added the implication of this antimicrobial agent, i.e NLITBO in pharmaceutical industry.

Reviewer comment 3

Methodology

Add new separate heading with detail of reagents/chemicals, instrumentation, media etc. used in present study.

Author response: We have done the modification as suggested.

Reviewer comment 4

-Line # 136-138: Add the reference.

Author response: We have added the reference as suggested

Reviewer comment 5

-Line # 139: Reference

Author response: We have added the reference as suggested

Reviewer comment 6

-Add the references in each section of Methodology.

Author response: We have added the reference as suggested

Reviewer comment 7

Results

-Make sure bacterial species name must be written in correct form in all text.

Author response: We have done the modification as suggested.

Reviewer comment 8

Discussion

-Line #: 282, 283: This is the part of result section

Author response: We have done the modification as suggested.

Reviewer comment 9

-Line # 286-289: Why ZOI produced by NLITBO were higher than those of chlorhexidine, give strong reason.

Author response: Mean ZOI produced by NLITBO is not higher but not significantly different from mean ZOI produced by Chlorhexidine. As per your valuable suggestions I have substantiated with possible reasons in the discussion.

Reviewer comment 10

-Line # 292: Add reference.

Author response: Those empty parentheses is to write the values as found in this study. We have added the details.

Reviewer comment 11

-Line # 296, 297: Cite the proper reference.

Author response: We have added the reference as suggested

Reviewer comment 12

-Line # 302: Reference?

Author response: We have added the reference as suggested

Reviewer comment 13

-Line # 320: Reference?

Author response: We have added the reference as suggested

Reviewer comment 14

-S. mutans, correct it with same pattern in all text.

Author response: We have done the modification as suggested.

Reviewer comment 15

-Add the explanation, how the NPs affect on the biofilm producing bacterial structure?

Author response: We have added the explanation as suggested

Reviewer comment 16

-Lines # 345-348: Unnecessary statement. Is this related to this study?

Author response :Thank you for raising the concern. We believe the sentences are not explanatory enough for which we have edited these lines again. These sentences are a critical analysis of the studies which were conducted to extablish the effectiveness of TBO as a photosensitizer. Only one study has used the clinical strain similar to our study. In general, clinical strains exhibit more genetic diversity and the findings on clinical strains are applicable in clinical settings. Therefore, we have highlighted the broader applicability of our results. Similarly contradicting our establishment of antibacterial efficacy of NLITBO, two studies could not prove clinical effectiveness of TBO in early childhood caries, which may be attributed to the use of laboratory grade TBO, lack of appropriate delivery method and/or lack of optimum light parameters. In this light we have discussed the importance of nanoliposome as a drug delivery system.
