# Supplementary material for: Exploring the photosensitizing potential of Nanoliposome Loaded Improved Toluidine Blue O (NLITBO) Against Streptococcus mutans: An in-vitro feasibility study
Source: PLoS One. 2024 Oct 30;19(10):e0312521. doi: 10.1371/journal.pone.0312521 (PMC11524459; doi:10.1371/journal.pone.0312521)
Supplement: Attachment — Submitted filename: Response to reviewer comments.docx [file pone.0312521.s004.docx]

Reviewer's Responses to Questions

1. Authors must know how to cite

Answer – Thank you so much. We have changed the parenthesis to square brackets as per the journal requirements.

2. This is not the appropriate way to write for methodology.(materials and instrumentations)

Answer – Thanks for raising the concern. We did this in the first revision as it was asked by one of the reviewers during the first revision. However, we revised it again as per your kind suggestion.

3. Too many typo mistakes in methodology

Answer –Thank you for rightfully pointing out the typo mistakes. We have corrected those mistakes.

4. As mentioned earlier, though strains were isolated earlier, they must have accession or ATCC numbers.pls mention here.

Answer –Thank you for the insightful comment. We have not procured this strain from any commercial source, for which we are unable to provide th ATCC number. We have also not submitted the sequencing data to any database for which we do not have accession number either. However, we have identified the bacteria based upon colony morphology, Gram's stain and sugar fermentation tests.

5. Pls do perform an in vitro antibiofilm assay before drawing final conclusion.

Answer –Our study was designed as a preliminary investigation to explore the bactericidal efficacy of novel photosensitiser, i.e NLITBO against *Streptococcus mutans* in a planktonic state. As planktonic cultures provide a controlled environment to evaluate the direct impact of the photosensitizer, this approach allows for a clear understanding of the fundamental antibacterial activity before extending the research to more complex biofilm models and is a common practice in antimicrobial research. This approach allows for fine-tuning the photosensitizer’s parameters under simpler conditions, which is crucial before addressing the added complexities of biofilms. Evaluating the efficacy in biofilm would require further optimization of photosensitizer parameters such as light exposure, penetration, and dosage, which can be the extension of this work. In fact, there are several published studies which have followed a similar approach, starting with the evaluation of novel photosensitizers in planktonic cultures of *Streptococcus mutans* before progressing to biofilm studies, which are summarised below.

| Author/ year | DOI | Photosensitiser |
| --- | --- | --- |
| Marco Aurelio Paschoal, 2014 | <https://doi.org/10.1089%2Fpho.2013.3656> | Curcumin Vs Toluidine blue |
| Anna Carolina Borges Pereira a Costa, 2010 | <https://doi.org/10.1590/S1806-83242010000400007> | Erythrosine Vs Rose bengal |
| Juliana P.M.L. Rolim, 2011 | <https://doi.org/10.1016/j.jphotobiol.2011.10.001> | Methylene blue ,Toluidine blue ortho ,Malachite green ,Eosin, Erythrosine and Rose bengal |
| Maria Ângela Lacerda Rangel Esper,2020 | <https://doi.org/10.14295/bds.2020.v23i2.1857> | Erythrosine |
| Caroline C Tonon,2015 | <https://doi.org/10.5005/jp-journals-10024-1626> | Curcumin |
| Marco Aurelio Paschoal,2013 | <https://doi.org/10.1016/j.pdpdt.2013.02.002> | Curcumin |

6. SEM and TEM images are not clear

Answer –Thank you for the suggestion. We have shot another photomicrograph with better resolution and Figure 2 has been updated.

7. Drug release graphs are not showing the control curve

Answer –Thank you very much. We ran the experiment along with the control and duly uploaded another graph (new Figure 3)
